# Aging‐Induced Ductile‐Brittle‐Ductile Transition in High‐Entropy Alloys and its Implications

**DOI:** 10.1002/advs.202510808

**Published:** 2025-10-27

**Authors:** Qianning Dai, Chenzhi Xing, Bijun Xie, Ming‐Hsien Lee, Bin Xu, Shaofei Ren, Yujie Song, Chunyang Wang, Mingyue Sun, Dianzhong Li

**Affiliations:** ^1^ Key Laboratory of Nuclear Materials and Safety Assessment Institute of Metal Research Chinese Academy of Sciences Shenyang 110016 China; ^2^ School of Materials Science and Engineering University of Science and Technology of China Shenyang 110016 China; ^3^ Shenyang National Laboratory for Materials Science Institute of Metal Research Chinese Academy of Sciences Shenyang 110016 China; ^4^ Department of Mechanical and Aerospace Engineering University of California Irvine Irvine CA 92697 USA; ^5^ Department of Physics Tamkang University Tamsui New Taipei Taiwan 25137 China

**Keywords:** chemical order, ductile‐brittle‐ductile transition, high‐entropy alloy, phase boundary, strain localization

## Abstract

Temper embrittlement, characterized by a dramatic loss of ductility within a narrow temperature window, is ubiquitous in conventional alloys but is not reported in compositionally complex or high‐entropy alloy systems. Here, an unexpected ductile–brittle–ductile transition is discovered in a multiphase high‐entropy alloy (HEA) aged in an intermediate temperature regime. Unlike the classical thermal embrittlement driven by grain boundary effects, this transition originates from the dynamic evolution of local chemical order (LCO) and phase boundary (PB) configurations in HEAs. Aging within the embrittlement‐prone regime enhances chemical ordering, increases the density of ordered domains, and induces jagged PBs, collectively triggering plastic instability and the ductile−to−brittle transition. In contrast, aging outside this regime suppresses excessive ordering and promotes the formation of ductile interphase transition zones, facilitating a brittle−to−ductile recovery. The findings offer new insights into the thermal behavior of HEAs and challenge the established paradigm of thermal embrittlement. These insights provide valuable guidance for the design and processing of high‐performance HEAs, thereby unlocking their potential as advanced high‐temperature structural materials.

## Graphical abstract

We discover a ductilebrittleductile transition in a multiphase high‐entropy alloy during intermediate‐temperature aging. The loss and recovery of ductility are controlled by dynamic evolution of local chemical order and phase boundary structure. Excessive ordering and jagged boundaries trigger localization and fracture, whereas moderated order and CRPs‐free PBs restore plastic stability, defining a processing window for strong, damage‐tolerant HEAs.

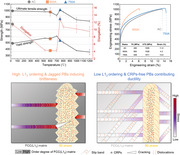

## 1 Introduction

The classical “temper embrittlement” phenomenon^[^ ^1^, ^2^, ^3^, ^4^, ^5^, ^6^, ^7^ ^]^—documented for over a century in conventional steels and other alloys—occurs upon exposure to specific temperature ranges, sharply reducing toughness and endangering engineering safety in critical applications such as aerospace, shipping, and petrochemical systems.^[^ ^8^, ^9^, ^10^ ^]^ Temper embrittlement degradation is primarily attributed to the segregation of embrittling elements or the precipitation of brittle phases along grain boundaries (GBs), which act as stress concentrators and crack initiation sites, ultimately inducing brittle fracture under stress.^[^ ^1^, ^2^, ^3^, ^4^, ^5^, ^7^, ^11^, ^12^, ^13^, ^14^ ^]^ This well‐established mechanism highlights the central role of brittle phase precipitation and element segregation at GBs in governing embrittlement in conventional alloys.

In contrast, emerging high‐entropy alloys (HEAs),^[^ ^15^, ^16^, ^17^ ^]^ which consist of multiple principal elements, introduce new thermodynamic and kinetic complexities that challenge conventional thermal exposure paradigms. Their high configurational entropy and sluggish diffusion kinetics^[^ ^17^, ^18^, ^19^, ^20^, ^21^, ^22^, ^23^ ^]^ suppress rapid grain coarsening and the formation of deleterious intermetallic phases compared to traditional alloys,^[^ ^17^, ^23^, ^24^ ^]^ endowing HEAs with exceptional strength‐ductility combinations, resistance to high‐temperature softening and low‐temperature embrittlement, and excellent wear resistance, making them attractive for demanding engineering applications.^[^ ^17^, ^25^, ^26^, ^27^ ^]^ Nonetheless, even HEAs designed as single‐phase solid solutions can develop subtle local chemical orders (LCOs)^[^ ^28^, ^29^, ^30^ ^]^ and even partition into multiple phases as they equilibrate,^[^ ^31^ ^]^ which seems inevitable due to the presence of elements with varying atomic sizes, electronegativities, and thermodynamic enthalpies of mixing. Recently, these unique LCOs have been successfully tailored to improve the strength‐ductility synergy of HEAs,^[^ ^32^, ^33^, ^34^ ^]^ which provides a new strategy for designing high‐performance HEAs. However, the thermodynamic and kinetic behaviors of these LCOs have still not been deeply investigated, especially the evolution of these LCOs under thermal exposure, which could be complicated due to the intrinsic compositional complexity. It is unknown whether these evolved LCOs will degrade the mechanical properties of HEAs, which is crucial for the engineering application of these high‐performance HEAs strengthened by LCOs. Furthermore, it remains unresolved whether HEAs undergo the tempering‐like embrittlement, and if so, how their unique multi‐element chemistry and microstructure influence such thermal brittleness.

Here we address this open question by delving into the potential of thermal embrittlement of a typical AlCoCrFeNi_2.1_ eutectic HEA (EHEA) with face‐centered cubic (FCC) and ordered body‐centered cubic (B2) phases.^[^ ^35^ ^]^ Compared with conventional alloys and other HEAs (Figure S1, Supporting Information), AlCoCrFeNi_2.1_ EHEA offers a balanced combination of high strength and superior ductility with the superior castability of eutectic alloys, allowing for direct casting of complex components and reducing manufacturing costs. Moreover, this alloy also exhibits exceptional high‐temperature strength and corrosion resistance, making it a promising candidate for high‐temperature structural and marine engineering applications.^[^ ^35^, ^36^, ^37^, ^38^, ^39^ ^]^ A key microstructural feature contributing to these properties is the presence of local chemical/ordering fluctuations, which significantly affect the mechanical behavior by altering the local stacking fault energy and dislocation dynamics.^[^ ^29^, ^30^, ^32^, ^34^, ^40^, ^41^, ^42^, ^43^, ^44^ ^]^ In the FCC phase, such fluctuations often drive the formation of locally ordered L1_2_ domains.^[^ ^45^, ^46^, ^47^ ^]^ Conversely, within the Ni and Al‐rich B2 phase, the limited solubility of Cr gives rise to local fluctuations that result in disordered regions, often manifesting as locally Cr‐rich BCC precipitates.^[^ ^48^, ^49^ ^]^ Notably, the local chemical/structural fluctuations across both phases evolve via thermal exposure, which might influence the susceptibility of thermal stability. The other feature is the intricate phase boundaries (PBs), with their complex atomic structures and unique interfacial configurations, which also play a critical role in determining the properties of alloys.^[^ ^50^, ^51^, ^52^ ^]^ In conventional alloy steels, embrittling phases and elements tend to segregate at grain or phase boundaries, thereby prompting further inquiry into whether similar mechanisms might induce thermal embrittlement in HEAs.

Employing thermal aging treatments from intermediate to high temperatures, we discover an unusual ductile−brittle−ductile transition in the AlCoCrFeNi_2.1_ HEA. Our findings reveal that the embrittlement transition is driven by the thermal‐induced evolution of LCOs and PBs' configurations. While generally beneficial under certain conditions, these features may act as precursors to embrittlement if altered unfavorably during aging. Specifically, aging‐enhanced L1_2_ domains combined with jagged PBs deteriorate the alloy's ductility. Intriguingly, aging‐induced ductile transition zones along the PBs and a reduced degree of order in L1_2_ domains at non‐embrittlement temperature ranges contribute to the regain of ductility. These findings challenge established theories and offer new insights into the thermal characteristics of compositionally complex alloy systems under different aging conditions.

## 2 Results

### 2.1 Ductile−Brittle−Ductile Transition in the HEA

**Figure 1:**
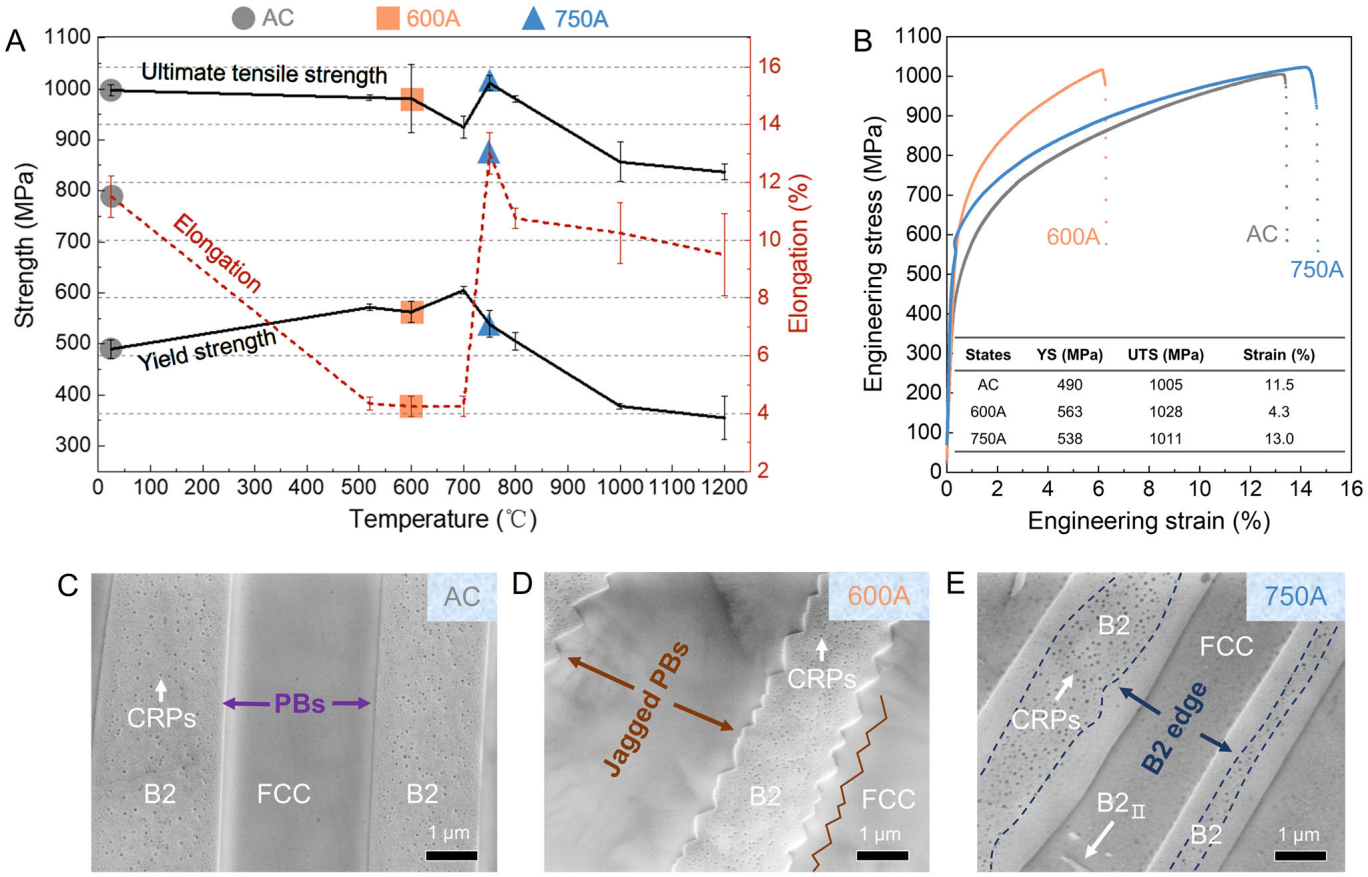
Mechanical properties and microstructures of high‐entropy alloys (HEAs) aged at different temperatures. A) Mechanical properties of the aged alloys at various aging temperatures. B) Engineering stress‐strain curves and corresponding performances of the AC, 600A, and 750A alloys. The inset table in (B) exhibits the yield strength (YS), ultimate tensile strength (UTS), and fracture strain of the AC, 600A, and 750A alloys. C−E) Morphologies of the FCC/B2 phase boundaries (PBs) in the AC, 600A, and 750A alloys, respectively. The unique jag‐shaped FCC/B2 PBs in the 600A alloy are highlighted in (D). The Cr‐rich particles (CRPs) are absent within the B2 edge region in the 750A alloy, as shown in (E).

**Figure** **1**A presents the tensile properties of AlCoCrFeNi_2.1_ HEA subjected to various aging treatments at different temperatures. For the as‐cast (AC) alloy or those aged at low temperatures, the ultimate tensile strength (UTS), yield strength (YS), and high fracture elongation maintain a relatively stable level, indicating ductile deformation behavior. As the aging temperature increases from ≈500 to 700 °C, fracture elongation deteriorates significantly, demonstrating a transition toward brittleness. This brittleness is accompanied by a reduction in UTS and a slight increase in YS at 700 °C. Intriguingly, at 750 °C, the UTS and YS recover to levels comparable to the AC alloy, and fracture elongation rises dramatically, thereby restoring ductility. Even after aging at an extreme temperature of 1200 °C, the UTS remains substantial at 837 MPa, and the YS holds at 355 MPa. Although fracture elongation declines at temperatures above 750 °C, the alloy aged at 1200 °C still retains a notable elongation of 9.5%. Overall, the UTS, YS, and fracture elongation exhibit high thermal stability at elevated temperatures. However, an unusual ductile−brittle−ductile transition is observed across 550 to 750 °C. Figure 1B further provides engineering tensile stress‐strain curves for the alloys of as‐cast state (AC), aged at 600 °C (600A), and aged at 750 °C (750A), demonstrating that the 750A alloy exhibits the optimal balance of ductility and strength, whereas the 600A alloy shows the poorest ductility. Moreover, this ductile−brittle−ductile transition is also confirmed by the impact toughness measurements, where the 600A alloy shows the lowest impact energy absorption (Figure S2, Supporting Information).

### 2.2 Microstructural Evolution of the AC, 600A, and 750A Alloys

To understand the unique ductile−brittle−ductile transition at intermediate temperatures, we first characterized the microstructures and phase distribution of the AC, 600A, and 750A alloys. X‐ray diffraction (XRD) results (Figure S3A, Supporting Information) confirm that these three alloys contain only FCC and B2 phases after aging treatments. The AC alloy shows a typical duplex‐phase microstructure (Figure 1C). It consists of an FCC matrix, which is enriched in Fe, Co, and Cr elements and occupies ≈69.2% of the volume, and a dispersed B2 phase that is rich in Al and Ni (Figure S3, Supporting Information). Within the B2 phase, numerous nanometer‐sized Cr‐rich particles (CRPs) are identified, previously characterized as having a BCC structure.^[^ ^53^, ^54^ ^]^ In the AC alloy, the CRPs contain a small amount of Fe, Co, Ni, and Al elements and gradually decrease in size as they approach the PB (Figure S3J, Supporting Information).^[^ ^55^ ^]^ The 600A alloy retains the same duplex‐phase microstructure as the AC alloy (Figure 1D) without significant phase and grain coarsening (Figure S3, Supporting Information). Notably, most PBs in the 600A alloy display a jagged morphology with dimensions in hundreds of nanometers (Figure 1D). However, the CRPs embedded in the B2 phase of 600A alloy exhibit a higher Cr content than those in the AC alloy (Figure S4, Supporting Information). Similarly, the 750A alloy also preserves the duplex‐phase microstructure and show no obvious coarsening (Figure 1E; Figure S3, Supporting Information). The CRPs in the 750A alloy have compositional features similar to those in the AC alloy (Figure S4, Supporting Information). However, these CRPs disappear within a region adjacent to the PBs, which is about a few hundred nanometers in scale (Figure 1E). This clean area at the edge of the B2 phase is notably different from that observed in both AC and 600A alloys. Moreover, the 750A alloy contains numerous fine precipitates enriched in Al and Ni and depleted in Fe, Co, and Cr elements (Figure S3, Supporting Information). These precipitates have a B2 structure and maintain a similar Kurdjumov‐Sachs (K‐S) relationship with the FCC matrix.^[^ ^53^ ^]^ To distinguish these fine B2 precipitates from the primary B2 phase formed via eutectic reaction during solidification, we denote them as B2_II_ phases.

### 2.3 LCOs evolution in the AC, 600A, and 750A Alloys

**Figure 2:**
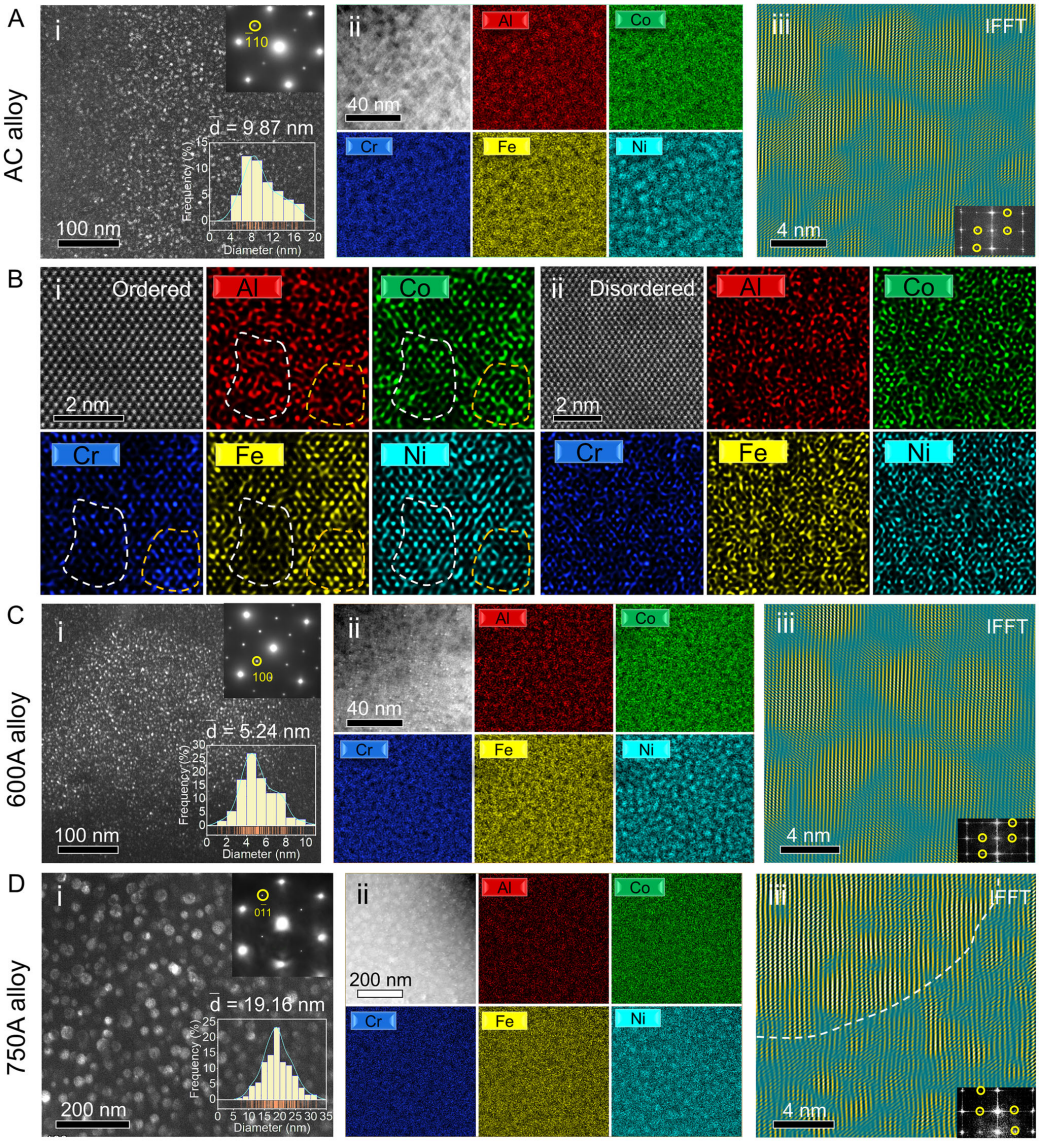
Atomic‐scale characterization of L1_2_ domains in the AC, 600A, and 750A alloys. A(i), C(i), and D(i) DF‐TEM images of L1_2_ domains in the AC, 600A, and 750A alloys, respectively. These images are obtained using the superlattice spots marked by the yellow circles in the SAED patterns at the top right corner. The size distribution histograms of the L1_2_ domains are shown in the bottom right corner. A(ii), C(ii), and D(ii) STEM‐EDS mappings of L1_2_ domains in FCC(L1_2_) matrix in the AC, 600A, and 750A alloys, respectively. A(iii), C(iii), and D(iii) IFFT images obtained from two series of L1_2_ superlattice spots marked by the yellow circles in the FFT pattern under the [112] zone axis. The brightness in these IFFT images represents the degree of ordering, i.e., the brighter the color, the higher the degree of ordering. The region enclosed by the white dotted line in D(iii) indicates part of a single L1_2_ domain. B(i−ii) Atomic‐scale STEM‐EDS mappings of ordered and disordered regions, showing the local chemical fluctuations in the FCC(L1_2_) matrix.

Given that the soft FCC matrix accommodates the majority of plastic strain during deformation in dual‐phase alloy,^[^ ^56^ ^]^ we explored its structural and chemical features (**Figure** **2**). Selected‐area electron diffraction (SAED) patterns of the FCC matrix display the ordered L1_2_ superlattice spots (Figure 2), indicating the existence of L1_2_ ordering within the FCC matrix. For clarity, this matrix is denoted as the FCC(L1_2_) matrix in subsequent discussions. In the AC alloy, the dark field TEM (DF‐TEM) image (Figure 2A(i)) reveals abundant nanometer‐sized spherical L1_2_‐ordered domains embedded in the FCC(L1_2_) matrix. The size distribution histogram in the lower right corner shows that these domains have an average diameter of ≈9.87 nm and constitute ≈23% of the volume. Scanning transmission electron microscopy‐energy dispersive X‐ray spectroscopy (STEM‐EDS) mapping confirms that these nanometer‐sized L1_2_ domains are enriched in Al and Ni and depleted in Fe, Co, and Cr (Figure 2A(ii)). High‐resolution high‐angle annular dark‐field (HAADF) image reveals that these regions maintain a fully coherent relationship with the FCC phase (Figure S5, Supporting Information). Further structural details of L1_2_ domains are elucidated by the inverse fast Fourier transform (IFFT) image acquired from two series of superlattice spots under [112] zone axis in the FFT pattern (Figure 2A(iii)). In the AC alloy, L1_2_ domains display imperfectly spherical shapes and interconnect through areas of weaker ordering. Notably, centers of L1_2_ domains display stronger ordering than their periphery, suggesting local fluctuations in ordering. Atomic‐scale STEM‐EDS mapping (Figure S6, Supporting Information) indicates that the Al and Ni‐enriched regions coincide with ordered areas at atomic scale, demonstrating a coupled local chemistry and L1_2_‐ordering fluctuation,^[^ ^44^ ^]^ which reveals that chemical variations contribute to differences in ordering. Comparison of atomic‐scale STEM‐EDS maps between nanometer‐sized ordered and disordered regions reveals that in ordered regions, Al‐Ni and Fe‐Co‐Cr groups form with significant local fluctuations (Figure 2B(i)), whereas disordered regions display a more random element distribution (Figure 2B(ii)).

Aging treatments significantly impact the L1_2_ domains. DF‐TEM images of the 600A and 750A alloys (Figure 2C,D(i)) show that the L1_2_ domains maintain a spherical feature similar to that observed in the AC alloy. However, their average diameters differ, measuring ≈5.24 nm after aging at 600 °C and increasing to an average of ≈19.16 nm after aging at 750 °C. The volume fractions of the L1_2_ domains are ≈30% in the 600A alloy and 27% in the 750A alloy. STEM‐EDS mappings (Figure 2C,D(ii)) indicate that, although the compositional characteristics of the L1_2_ domains remain similar to those in the AC alloy, the 600A alloy displays a more pronounced compositional interface between the L1_2_ domain and FCC phase compared to both AC and 750A alloys. Similar to the AC alloy, the L1_2_ domains in the aged alloys maintain a coherent relationship with their surrounding FCC matrix (Figure S5, Supporting Information). IFFT images confirm that local ordering fluctuation within L1_2_ domains is retained in both 600A and 750A alloys (Figure 2C,D(iii)). Atomic strain maps of the FCC(L1_2_) matrix obtained via geometrical phase analysis (GPA)^[^ ^57^ ^]^ reveal distinct strain features among the three alloys, potentially influencing dislocation movement and strain hardening behavior of the alloys (Figure S7, Supporting Information).^[^ ^58^ ^]^

### 2.4 Elevated Order Degree of L1_2_ Domains in the 600A Alloy

**Figure 3:**
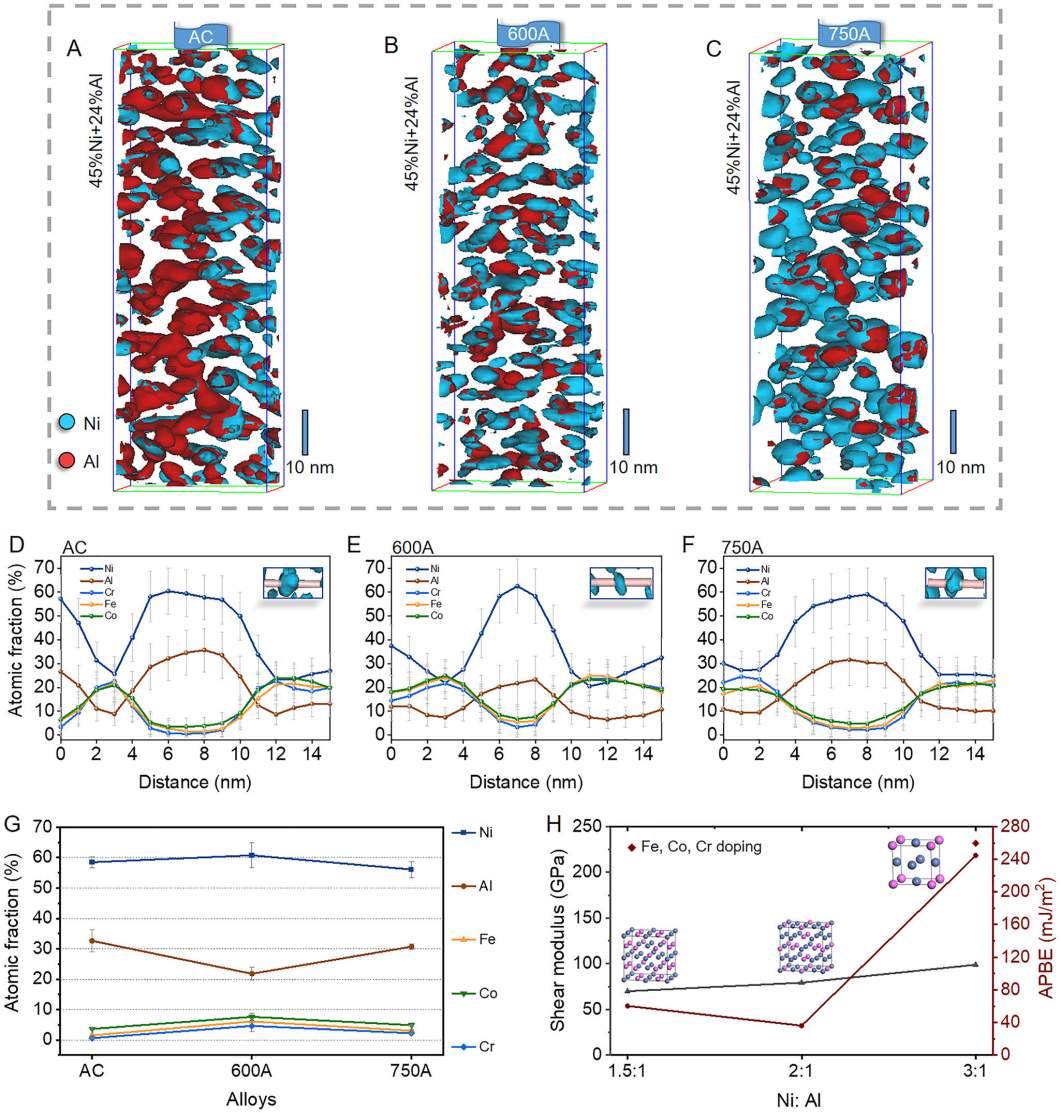
Chemical evolution of the L1_2_ domains in three alloys characterized by APT. A−C) Isoconcentration surface maps (45% Ni+24% Al) of the AC, 600A, and 750A alloys, respectively. The Ni and Al elements are displayed using blue and red colors. D−F) 1D concentration profiles of L1_2_ domains in the AC, 600A, and 750A alloys, respectively. The image in the upper right corner represents the measured L1_2_ domain. (E) shows a decrease in Al content alongside increases in Ni, Fe, Co, and Cr concentrations within the L1_2_ domains of the 600A alloy. (D) and (F) show that the L1_2_ domains in AC and 750A alloys have similar compositions. G) Elemental variations within L1_2_ domains across all three alloys, indicating increases in Ni, Fe, Co, and Cr levels and a decrease in Al content in the 600A alloy. These data represent the average statistical results of the chemical compositions of fifteen distinct L1_2_ domains. H) DFT results of the influence of composition on the shear modulus and APBE of L1_2_ domains. The inset crystalline structures are the calculated models.

Atom probe tomography (APT) was employed to further elucidate the chemical characteristics of the L1_2_ domains in the AC, 600A, and 750A alloys (**Figure** **3**). Consistent with STEM‐EDS results, all three alloys present high‐density Ni and Al‐rich domains (Figure S8, Supporting Information). The isoconcentration surface maps of the FCC(L1_2_) matrix (Figure 3A−C) reveal that the 600A alloy contains a particularly high density of L1_2_ domains with the smallest size. 1D concentration profiles (Figure 3D−F) show that, while the L1_2_ domains in both AC and 750A alloys share similar compositional characteristics, those in the 600A alloy differ markedly. Specifically, the L1_2_ domains in the 600A alloy display a significantly lower Al content, and the atomic ratio of Ni/Al is ≈3:1, which keeps the Ni/Al ratio of a perfectly L1_2_‐ordered Ni_3_Al structure (Figure 3G; Table S1, Supporting Information). In contrast, the Ni/Al ratio is ≈2:1 in the AC and 750A alloys, indicating that L1_2_ domains in these two alloys are off‐stoichiometric L1_2_ with decreased order degrees. Moreover, slightly higher amounts of Fe, Co, and Cr are observed in the L1_2_ domains in the 600A alloy compared to their counterparts in the AC and 750A alloys. Nevertheless, the total amount of these three elements is low in the L1_2_ domains in these three alloys.

Density functional theory (DFT) calculations were conducted to clarify the impact of these compositional changes on the shear modulus and antiphase boundary energy (APBE) of L1_2_ domains (Figure S9 and Table S2, Supporting Information). The DFT results in Figure 3H reveal that a reduction in Al content in the L1_2_ domains leads to a significant increase in shear modulus. More significantly, as the atomic ratio of Ni: Al changes from nearly 2:1 to 3:1, the APBE also increases substantially. Moreover, DFT calculations reveal that minor doping of Fe, Co, and Cr can slightly lead to an increase in APBE, as indicated by the red rhombus in Figure 3H. Collectively, these findings suggest that the L1_2_ domains in the 600A alloy exhibit a higher APBE and an expanded shear modulus, compared to those in the AC and 750A alloys.

### 2.5 Deformation and Cracking Behaviors of the AC, 600A, and 750A Alloys

**Figure 4:**
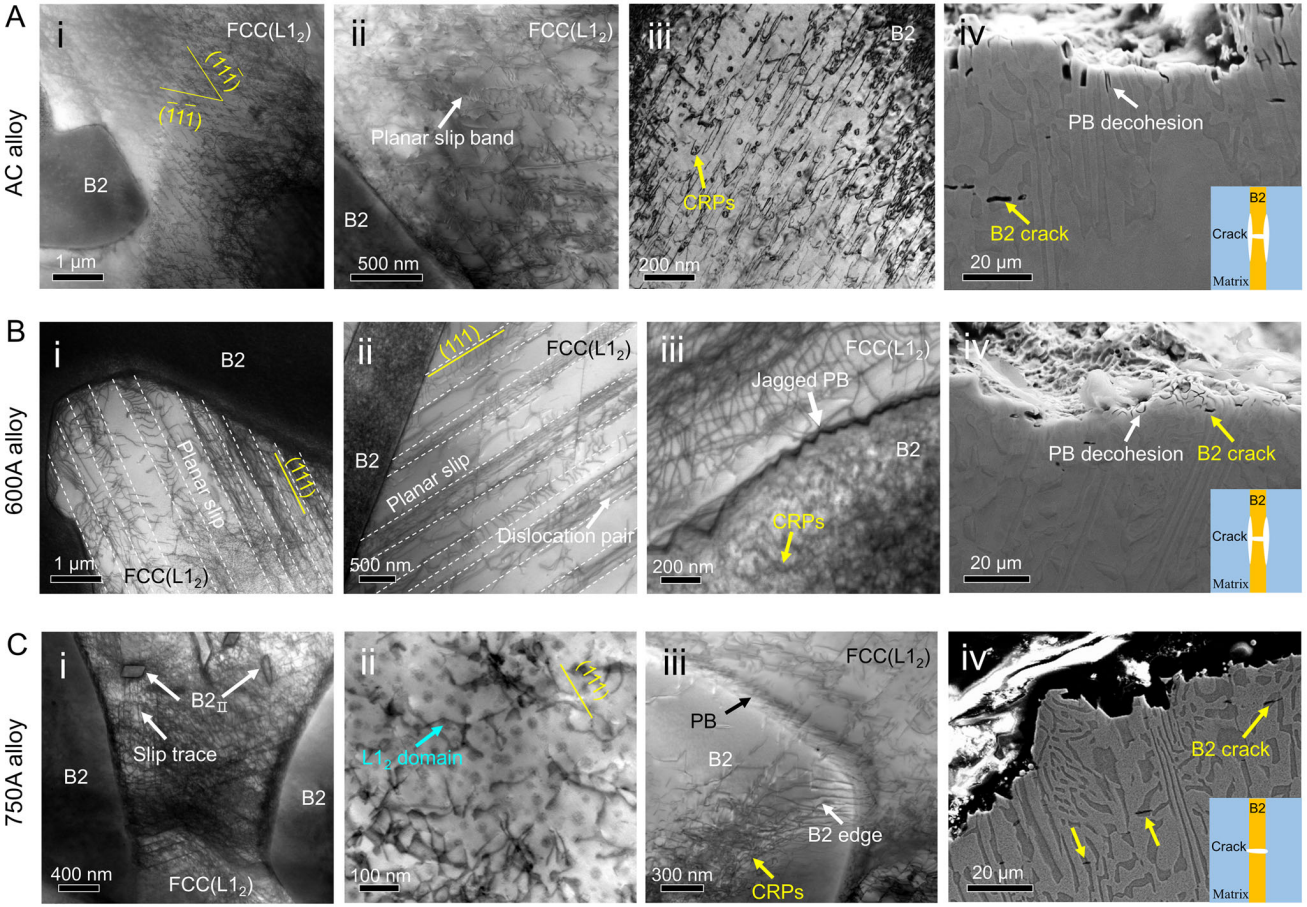
Different dislocation patterns and fracture characteristics of the deformed AC, 600A, and 750A alloys. A(i), B(i), and C(i) Bright field‐STEM (BF‐STEM) images of slip traces along the {111} slip planes in FCC(L1_2_) matrix of the AC, 600A, and 750A alloys, respectively. A(ii) Multiple dense slip bands in the FCC(L1_2_) matrix in the AC alloy. A(iii) Deformation microstructure of the B2 phase in the AC alloy, showing the strong pinning effect of CRPs on dislocation movement. B(ii) Heavily localized dislocation slip within the planar slip bands and long dislocation pairs in the 600A alloy. B(iii) Deformation behaviors of B2 phase and jagged PB in the 600A alloy. C(ii) Interaction of nanometer‐sized L1_2_ domains and dislocations in the 750A alloy. C(iii) Deformation characteristics of the B2 phase and PB in the 750A alloy. A(iv), B(iv), and C(iv) Fracture cross‐section images of the AC, 600A, and 750A alloys, respectively.

To elucidate the underlying mechanisms governing the mechanical response of the alloys with distinct microstructures, we investigated their deformation and fracture features (**Figure** **4**). In the AC alloy, the FCC(L1_2_) matrix exhibits dense slip traces aligned with various {111} slip planes (Figure 4A(i)), indicating activation of multiple slip systems. Slightly planar slip bands indicated by white arrows in Figure 4A(ii) suggest that dislocations predominantly operate through a planar slip mechanism in the FCC(L1_2_) matrix, which can be attributed to L1_2_ ordering.^[^ ^59^ ^]^ Within the B2 phase, numerous arrow‐shaped dislocations are observed around the CRPs (yellow arrows in Figure 4A(iii)), indicating that these CRPs impose a strong pinning force to hinder dislocation movement. The deformation features of the 600A alloy are markedly different. The FCC(L1_2_) matrix here shows more remarkable planar slip bands along only one of the {111} planes (Figure 4B(i),(ii). Regions between these planar slip bands experience minimal deformation, while dislocations densely accumulate and form significant pile‐ups at the B2/FCC(L1_2_) PBs (Figure S10A, Supporting Information). The high frequency of these single‐orientation planar slip bands makes the FCC(L1_2_) matrix more susceptible to local strain concentration. Moreover, we identified many unique long dislocation pairs characterized by leading and trailing dislocations (white arrow in Figure 4B(ii)). These pairs display a curved morphology and are conspicuously entangled and accumulated (Figure 4B(i); Figure S10B, Supporting Information). This suggests that the dislocation mobility is strongly hindered, subsequently impacting the overall ductility of the 600A alloy. The B2 phase also demonstrates a CRPs‐induced pinning effect on dislocation motion as seen in the AC alloy (yellow arrow in Figure 4B(iii)). High‐density dislocations are observed to accumulate at the jagged PBs, which indicates high stress concentration (Figure 4B(iii)). In contrast, the FCC(L1_2_) matrix in the 750A alloy exhibits complex dislocation networks (Figure 4C(i)) that suggest the activation of the majority of {111}<110> slip systems, resulting in a more uniform plastic deformation. Moreover, multiple slip traces are obstructed by B2_II_ particles within the FCC(L1_2_) matrix (Figure 4C(i)). Meanwhile, the L1_2_ domains are observed to deform via a cutting‐through mechanism^[^ ^60^ ^]^ (see cyan arrow in Figure 4C(ii)). Notably, we observed that dislocations glide easily across the regions lacking CRPs around the B2/FCC(L1_2_) PBs (indicated by the white arrow in Figure 4C(iii)), while these gliding dislocations become pinned and entangled upon encountering the CRPs in the B2 phase. This results in a shell‐core configuration of dislocation distribution emanating from the PBs into the B2 interior (Figure S10D, Supporting Information), effectively mitigating stress concentration at the interface. This contrasts sharply with severe dislocation pile‐ups observed around PBs within both AC and 600A alloys (Figure 4A‐C(iii)). These observations suggest that this CRPs‐free zone located at the B2 edge region may confer superior ductility by alleviating stress concentration and suppressing pre‐cracking at phase boundaries.

Figure 4A‐C(iv) illustrate the fracture cross‐sections of the AC, 600A, and 750A alloys, respectively. In all three alloys, microcracks initiate within the B2 phase (Figure 4A‐C(iv)). Notably, the AC and 600A alloys exhibit significant tearing decohesion cracking along the PBs, as indicated by white arrows in Figure 4A(iv) and 4B(iv). In contrast, this phenomenon is absent in the 750A alloy (Figure 4C(iv)), suggesting enhanced interfacial strength and reduced stress concentration at the B2/FCC(L1_2_) PBs. Furthermore, the B2 phase across all three alloys displays a typical brittle fracture characteristic, evidenced by a river‐like fracture morphology, while the FCC(L1_2_) matrix exhibits a typical dimpled fracture morphology (Figure S11, Supporting Information). In the case of the 750A alloy, numerous small dimples are observed, which originate from the B2_II_ phases (Figure S11, Supporting Information), indicating an increased ductility in this alloy.

## 3 Discussion

### 3.1 Ductile−Brittle Transition

Our results demonstrate that aging the AlCoCrFeNi_2.1_ alloy at 600 °C induces a ductile−brittle transition, which is different from the general degradation mechanisms—such as phase transformation, grain coarsening, or GBs embrittlement—commonly observed in conventional alloys when suffering heat treatment.^[^ ^61^, ^62^, ^63^ ^]^ Instead, the transition originates from the coupled evolutions of LCOs and the B2/FCC(L1_2_) PBs.

**Figure 5:**
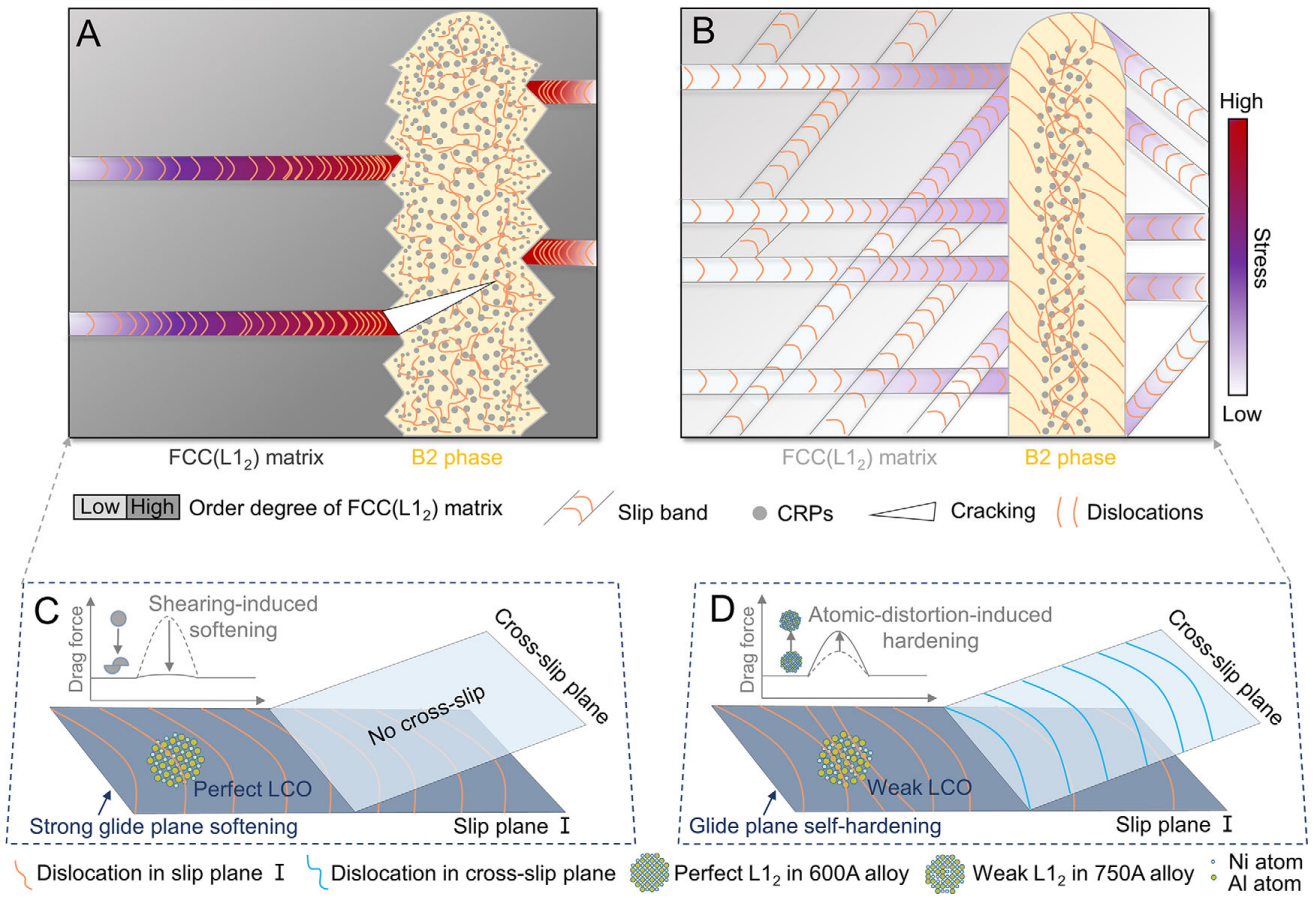
Schematic comparison of deformation mechanisms in the 600A and 750A alloys. A) Synergistic embrittlement in the 600A alloy. Strong LCO promotes planar dislocation slip and induces severe strain localization. Concurrently, jagged PBs act as sites for high‐stress concentration. B) Synergistic toughening in the 750A alloy. Moderate LCO facilitates the activation of multiple slip systems, leading to more homogeneous deformation. This effect is complemented by ductile PBs that accommodate plastic strain and mitigate local stress concentrations. C) In the 600A alloy, strong LCO causes glide‐plane softening, which confines dislocation activity to a single slip plane. The inset illustrates the drag force on a dislocation before and after it shears the ordered domains. D) In the 750A alloy, the weaker LCO lowers the barrier for dislocation cross‐slip, promoting distributed plasticity. The inset shows the corresponding drag‐force profile, indicating an increased resistance to dislocation motion.

In the 600A alloy, the higher‐density and smaller‐sized L1_2_ domains present perfectly ordered Ni_3_Al structure, exhibiting elevated APBE and elastic modulus (Figure 3), which raises the critical shear stress for dislocation motion.^[^ ^64^ ^]^ Although these ordered domains notably harden the FCC(L1_2_) matrix via both ordering and modulus strengthening (Figure S12 and Note S1, Supporting Information), they simultaneously enhance the brittleness by impeding dislocation motion and concentrating stress.^[^ ^65^, ^66^, ^67^ ^]^ Moreover, the ordered L1_2_ generally promotes planar dislocation slip through a “glide plane softening” effect (**Figure** **5**C), whereby dislocations successively cut through and degrade the ordered regions, reducing the glide resistance for subsequent dislocations and leading to the aggregation of dislocations (Figure S13, Supporting Information).^[^ ^68^ ^]^ Aging at 600 °C induces enhanced ordering with higher APBE, which intensifies this planarity, leading to pronounced strain localization and unsustainable working hardening (Figure S14A, Supporting Information). Concurrently, the superlattice intrinsic stacking fault energy (SISFE) is greatly affected by chemical substitutions; a lower SISFE decreases the likelihood of dislocation cross‐slip.^[^ ^69^, ^70^ ^]^ As demonstrated by Kumar et al.^[^ ^71^ ^]^ for L1_2_‐ordered Ni_3_Al, higher Al content increases SISFE; therefore, the Ni_3_Al‐like L1_2_ domains with lower Al content in the 600A alloy contribute to restricted dislocation cross‐slip and accentuate localized slip.

Parallel to these effects in the FCC(L1_2_) matrix, we observed a high density of jagged B2/FCC(L1_2_) PBs in the 600A alloy, which stands in sharp contrast to the smooth interfaces in the as‐cast state that promote continuous dislocation slip and excellent ductility.^[^ ^72^ ^]^ The jagged PBs consist of zig‐zag interface I and interface II with different orientations as indicated in Figure S17 (Supporting Information). Interface II, indexed as ($\bar{2}\bar{1}1$)_FCC_//($\bar{2}1\bar{3}$)_B2_, corresponds to the initial coherent and smooth segments preserved from the as‐cast state. In contrast, Interface I, indexed as ($\bar{7}5\bar{5}$)_FCC_//($23\bar{1}$)_B2_, emerges through the structural transformation of initially unstable smooth PBs during aging (Note S3, Supporting Information) and exhibits a more complex and defective structure with lower coherency and higher interfacial energy. Such jagged PBs destroy the preferred interfacial structures, reduce the slip‐transfer efficiency (Note S4, Supporting Information), compromise dislocation transmission, and foster local stress concentration that accelerates crack initiation and propagation, as revealed by transmission Kikuchi diffraction (TKD) analysis (Figure S14, Supporting Information).

The synergistic embrittlement resulting from L1_2_‐induced planar slip and serrated, low‐coherency PBs is illustrated in Figure 5A,C. First, enhanced LCOs ordering drives significantly localized‐planar slip bands in the FCC(L1_2_) matrix, which serve as the primary source of plastic instability. When these slip bands impinge upon the jagged PBs, especially the high‐resistance Interface I segments, dislocation pile‐ups concentrate shear stresses at the serration tips (Figure S14, Supporting Information). Simultaneously, aging‐induced hardening of the B2 phase, driven by the evolving CRPs (Figure S12, Supporting Information), diminishes its ability to accommodate the imposed strain. The mismatch in slip systems and stress‐relaxation capacity between the phases causes local stresses to rise rapidly at the sharp tips of the jagged PB. Once it exceeds the local yield or decohesion threshold, microcracks nucleate and propagate along these stress concentrators, producing brittle failure.

This embrittlement pathway is fundamentally distinct from classical thermal embrittlement in conventional alloys—such as temper embrittlement, intergranular‐corrosion and aging embrittlement—where fracture is typically intergranular due to GB cohesion loss from impurity segregation (e.g., S, Bi, P, As) or brittle intergranular precipitation (Table S3, Supporting Information). In contrast, the present eutectic HEA shows no analogous GB segregation, phase decomposition or intergranular precipitation. Here, embrittlement does not simply arise from boundary weakening, but from the microstructure's instability to dissipate plastic strain globally. Planar slip is funneled into narrow channels that terminate at serrated, low‐transmissivity PBs, leading to stress localization and crack formation. Detailed literature survey and experimental investigation (Table S4 and Note S2, Supporting Information) indicate that this coupled LCOs‐PBs embrittlement mechanism may be broadly applicable to typical EHEAs. The multi‐principal‐element chemistry of HEAs creates distinct thermodynamic and kinetic landscapes that favor strong partitioning, pronounced ordering fluctuations, and PB reconstructions not readily accessible in conventional alloys. These intrinsic attributes render eutectic HEAs particularly susceptible to this unique embrittlement mode.

### 3.2 Brittle−Ductile Transition

Aging at 750 °C leads to a regain of mechanical properties of the alloy, which exhibits an increased YS and a ductility nearly three times that of the 600A alloy. This brittle−ductile transition is primarily attributed to the decreased order degree of L1_2_ domains and the formation of ductile B2/FCC(L1_2_) PBs. In the 750A alloy, the size of L1_2_ domains increases to ≈19.16 nm, which is anticipated to amplify both the modulus hardening and ordering hardening of the FCC(L1_2_) matrix (Note S1, Supporting Information), resulting in a hardness that surpasses that of the AC alloy (Figure S12, Supporting Information). Crucially, these L1_2_ domains are off‐stoichiometric and only weakly ordered, which significantly alters the deformation behavior of the FCC(L1_2_) matrix. During deformation, dislocations glide through the weakly ordered L1_2_ regions, progressively randomizing Ni and Al atoms and generating substantial lattice distortion within the planar‐slip band. Unlike the abrupt breakdown of perfectly ordered L1_2_ domains in the 600A alloy, the gradual disordering in the 750A alloy produces increasing lattice strain that impedes dislocation motion (Figure 4C(ii)), resulting in a glide plane self‐hardening effect (Figure 5D).^[^ ^34^ ^]^ Consequently, planar‐slip behavior shifts to enable cross‐slip and the activation of multiple slip systems (Figure 5B), contributing to uniform plastic flow throughout the sample rather than the confined planar‐slip bands observed in the 600A alloy. This promotes the sustainable strain hardening and thus contributes to the high ductility in the 750A alloy (Figure S14A, Supporting Information).

The other key feature in the 750A alloy is the unique B2 edge region near B2/FCC(L1_2_) PBs, which notably lacks CRPs, namely the CRPs‐free zone. Its formation mechanism (Note S5, Supporting Information) is supported by thermodynamic/kinetic calculations and experiments. Thermodynamically, the CRPs are unstable at 750 °C, which tend to dissolve into the B2 matrix, increasing the Cr content in the B2 phase as indicated by phase diagram analysis (Figure S18A−C, Supporting Information). This leads to a higher chemical potential of Cr in the B2 phase compared to that in the FCC(L1_2_) matrix (Figure S18D, Supporting Information), establishing a strong driving force for Cr diffusion from the B2 phase into the FCC(L1_2_) matrix. Moreover, kinetic calculations (Figure S18E, Supporting Information) reveal that the Cr diffusion coefficient at 750 °C is sufficiently high to enable the Cr diffusion from B2 to FCC(L1_2_) matrix, which lowers the local equilibrium Cr concentration on the B2 side. To maintain chemical equilibrium, smaller CRPs adjacent to the PB—particularly those with high interfacial curvature—dissolve first, forming a CRP‐depleted zone along the B2 edge (Figure S18G, Supporting Information). This dissolution process ceases once the Cr concentration in the B2 matrix reaches the equilibrium solubility at this temperature, thereby promoting and stabilizing the CRP‐free zone within B2 (Figure S18H−I, Supporting Information). Nanoindentation tests (Figure S12, Supporting Information) reveal that this CRPs‐free zone has hardness similar to the FCC(L1_2_) matrix and lower than the B2 inner area. This is due to the absence of CRPs strengthening in the B2 edge areas, allowing deformation dislocation to transfer through PBs more easily, which enhances deformation compatibility and reduces stress concentration around the PBs. TKD analyses of deformed PBs in the 750A alloy (Figure S15, Supporting Information) support these findings. A lower density of geometrically necessary dislocations (GNDs) in the B2 edge area is observed compared to that in the B2 inner area, which indicates enhanced uniform plastic deformation and superior compatibility near the PBs.^[^ ^73^ ^]^ Additionally, a lower kernel average misorientation (KAM) in the B2 edge area than in the inner area suggests a reduced stress concentration at the PBs. Consequently, the B2/FCC(L1_2_) PBs in the 750A alloy no longer serve as rapid pathways for crack initiation due to the existence of this ductile transition zone, which is evidenced by the intact PBs in the tensile fracture cross‐section (Figure 4C(iv)). This contrasts with the AC and 600A alloys, which exhibit severe tearing and decohesion cracking at PBs. In addition to the excellent tensile performance, this kind of ductile PBs might improve the creep and fatigue properties of the aged alloy compared to the AC alloy.

This study delineates the temperature window over which the AlCoCrFeNi_2.1_ HEA undergoes a ductile−brittle−ductile transition, providing critical guidance for optimizing its heat‐treatment regime to achieve optimal combinations of strength and ductility for application. To establish a complete aging framework, we also performed aging treatment with varied dwell times across representative temperatures (Note S6, Supporting Information), further clarifying the kinetic sensitivities of both microstructure and mechanical properties and refining the operative time–temperature processing window. By elucidating the atomic origins—the evolution of LCOs and PBs—that drive embrittlement and subsequent recovery, we offer a roadmap for the design and microstructural customization of high‐performance multiphase HEAs through controlled LCOs and interface topology. Importantly, the ductile−brittle transition exhibits thermally reversible behavior, suggesting that this alloy system holds promise for recyclability and sustainable material applications. By applying appropriate heat treatments, the embrittled alloy can fully recover its high strength and ductility, enabling repeated reuse without performance degradation. Moreover, leveraging the well‐defined embrittlement window, one can engineer HEA rupture discs, shear pins, or over‐temperature disconnects that fail safely and predictably at fire‐relevant temperatures (≈500–600 °C).^[^ ^74^, ^75^ ^]^ Such “temperature fuses” would automatically relieve pressure or isolate circuits under thermal runaway, enhancing the intrinsic safety of boilers, pressure vessels, and chemical process lines. Future efforts will extend these paradigms to other HEAs to evaluate the universality and guide their safe high‐temperature application. We also plan to probe the coupled thermal‐mechanical‐chemical effects on LCOs and PBs evolution, with the goal of establishing thermomechanical processing strategies to tailor the mechanical performance of the HEA components.

## 4 Conclusion

In summary, our study deciphers an unusual ductile−brittle−ductile transition in a eutectic HEA, driven by the dynamic evolution of LCOs and PBs configurations. Enhanced LCOs and the formation of jagged PBs lead to brittleness, while controlling the order degree of LCOs and the formation of a ductile transition zone along the PBs recovers the ductility. This discovery highlights the critical role of nanoscale chemical heterogeneities and PBs in determining material behavior, challenging and expanding the traditional paradigm of thermal embrittlement. Although LCOs are generally beneficial for strength‐ductility synergy in HEAs, our findings reveal their dual role: excessive ordering induces brittleness, whereas controlled LCOs restore ductility, which further indicates the necessity of meticulous control of LCOs for better performance. We believe that the uncover of thermal evolution behavior of HEA will advance the high‐temperature application of the HEA family.

## 5 Experimental Section

### Sample Preparation and Aging Treatments

A raw ingot with a nominal composition of AlCoCrFeNi_2.1_ (at%) EHEA was prepared via vacuum induction levitation melting using pure elements (purity higher than 99.9%) under an argon atmosphere. To ensure compositional homogeneity, the ingot was flipped and remelted at least five times. Cylindrical specimens (6.5 mm diameter × 45 mm length) were extracted from the ingot for aging treatments. Each specimen was sealed in an evacuated quartz tube and subjected to isothermal aging at selected temperatures (520, 600, 700, 750, 800, 1000, and 1200 °C). The furnace was heated at a controlled rate of 300 °C/h to the target temperature. To ensure thermal stability and uniformity of the furnace chamber, the temperature was held constant for 3 h before introducing the specimens. The specimens were then isothermally aged at these temperatures for 20 h. To investigate time‐dependent aging effects, additional specimens were aged at 600 and 750 °C for varied durations of 5, 12, and 72 h. Following the aging treatment, all specimens were rapidly removed from the furnace, and the quartz tubes were broken in water to achieve rapid quenching.

### Tensile Tests

Tensile specimens with a gauge diameter of 3 mm and length of 20 mm were tested using an Instron 5982 testing machine at a constant strain rate of 1 × 10^−3^ s^−1^. Tensile tests follow the ISO 6892‐1:2016 standard. Three specimens per condition were tested to ensure reproducibility.

### Nanoindentation Tests

Nanoindentation tests were performed on both FCC(L1_2_) and B2 phases using a Bruker Hysitron PI 89 system with a maximum load of 5 mN and a 5 s holding time. At least six indentations were made per phase, spaced ≈3 µm apart (15× the indentation depth) to avoid interaction.

### APT Characterization

APT analysis of the FCC(L1_2_) matrix was conducted using a CAMECA LEAP 4000X HR. Needle‐shaped specimens were prepared via SEM lift‐out with a dual‐beam FIB (FEI Helios 600i). Three APT tips yielded datasets exceeding 30 million atoms each. Data acquisition parameters included a 50 K specimen temperature, 200 kHz pulse frequency, 60 pJ laser energy, and 1% evaporation rate. Reconstructions and analyses were performed using IVAS 3.6.8 software.

### Microstructures Characterization

Phase identification of as‐cast and aged samples was performed by X‐ray diffraction (XRD; Rigaku SmartLab 9 kW) with Cu‐Kα radiation (45 kV, 45 mA), scanning 2θ = 20–100° at 5°/min. Zeiss Supra 35 scanning electron microscope (SEM) was used to characterize the microstructures and fracture morphologies of all alloys. Talos F300X TEM equipped with EDS and an aberration‐corrected FEI Titan Themis TEM equipped with a Super‐X EDS were used to reveal the elaborate microstructures and deformation behaviors. Samples for aberration‐corrected TEM were cut and thinned to less than 60 nm by using a dual‐beam focused ion beam (FEI Helios 600i) via SEM lift‐out procedures. Samples for Talos F300X TEM analysis were ground to 50 µm by SiC paper and subsequently perforated by twin‐jet electro‐polishing using a solution of 10 vol% nitric acid alcohol solution at −20 °C. Transmission Kikuchi diffraction (TKD) and electron backscatter diffraction (EBSD) were conducted using the Zess Gemini 460 SEM equipped with EDS and EBSD. The specimens for TKD were the same TEM specimens used to characterize the deformation behaviors of alloys, and the scanning step size for all alloys was the same (15 nm). The specimens for EBSD were prepared by mechanical grinding and subsequently electrochemical polishing using a 10 vol% nitric acid alcohol solution at −25 °C. Both TKD and EBSD data analysis were conducted using AZtec Crystal software.

### DFT Calculations

Density functional theory (DFT) calculations were performed using the CASTEP^[^ ^76^ ^]^ to determine the elastic constants and APBEs. To investigate the influence of Al content on the properties of L1_2_ domains, supercell models (2 × 2 × 2) of Ni_1.5_Al and Ni_2_Al were developed (Figure S9, Supporting Information). Additionally, the doping effects of Fe, Co, and Cr on the L1_2_ domain were examined using the Virtual Crystal Approximation (VCA) method. Specifically, doping configurations were designed based on the chemical compositions of the L1_2_ domains in the AC and 600A alloys.

The APBEs were calculated using crystal cells generated via both VCA and supercell approaches. An eight‐layer model was created by cleaving the (111) plane, with a 15 Å vacuum layer to eliminate the effect of periodic boundary. The slip direction was defined as 1/2<110>. To minimize the influences of surface relaxation and reconstruction, the top and bottom atomic layers were fixed, while the remaining atoms were fully relaxed during geometric optimization. Spin polarization was incorporated in all calculations to account for magnetic contributions. The Cr pseudopotential was specified as Cr_01.recpot or Cr_00.recpot, while OTFG ultrasoft pseudopotentials were used for other elements to accurately describe electronic behavior. Exchange‐correlation interactions were treated using the Perdew‐Burke‐Ernzerhof (PBE) functional within the Generalized Gradient Approximation (GGA). The self‐consistent field (SCF) convergence threshold was set to 10^−5^ eV/atom, with force and stress convergence tolerances of 0.03 eV Å^−1^ and 0.05 GPa, respectively. The calculated APBE of Ni_3_Al agrees well with prior computational^[^ ^77^ ^]^ and experimental results.^[^ ^78^, ^79^ ^]^

To ensure the reliability of elastic constant calculations, k‐point sampling and pseudopotential selection were rigorously optimized. Therefore, a high‐density k‐point grid (separation < 0.02 Å^−1^) was adopted to enhance the resolution of electronic states near the Fermi level, which critically influences elastic responses.

## Conflict of Interest

The authors declare no conflict of interest.

## Author Contributions

Q.N.D. and B.J.X. wrote the original draft. M.Y.S., B.J.X., and Q.N.D. performed conceptualization. S.F.R., Y.J.S., and B.X. performed investigation. Q.N.D., B.J.X., M.Y.S., and C.Y.W. wrote, review and edited the final manuscript. Q.N.D., B.J.X., C.Z.X., and M.‐H.L. performed methodology. B.J.X., M.Y.S., and D.Z.L. performed supervision. M.Y.S. and B.J.X. performed funding acquisition. Q.N.D. and B.J.X. performed validation. Q.N.D. and B.J.X. performed formal analysis. Q.N.D. and B.J.X. performed Visualization. M.‐H.L. performed Software.

## Supporting information



Supporting Information

## Data Availability

The data that support the findings of this study are available from the corresponding author upon reasonable request.
