# Supplementary material for: Aging‐Induced Ductile‐Brittle‐Ductile Transition in High‐Entropy Alloys and its Implications
Source: Adv Sci (Weinh). 2025 Oct 27;13(3):e10808. doi: 10.1002/advs.202510808 (PMC12806431; doi:10.1002/advs.202510808)
Supplement: Supplementary file 1 — Supporting Information [file ADVS-13-e10808-s001.docx]

**Supporting Information for**

**Aging-induced ductile-brittle-ductile transition in high-entropy alloys and its implications**

Qianning Dai^1, 2, 3^, Chenzhi Xing^2^, Bijun Xie^1, 3, 4^*, Ming-Hsien Lee^5^, Bin Xu^1, 3^, Shaofei Ren^1, 2, 3^, Yujie Song^2, 3^, Chunyang Wang^3^, Mingyue Sun^1, 3^*, Dianzhong Li^3^

^1^ Key Laboratory of Nuclear Materials and Safety Assessment, Institute of Metal Research, Chinese Academy of Sciences, Shenyang 110016, China

^2^ School of Materials Science and Engineering, University of Science and Technology of China, Shenyang 110016, China

^3^ Shenyang National Laboratory for Materials Science, Institute of Metal Research, Chinese Academy of Sciences, Shenyang 110016, China

^4^ Department of Mechanical and Aerospace Engineering, University of California, Irvine, CA 92697, USA

^5^ Department of Physics, Tamkang University, Tamsui, New Taipei 25137, Taiwan

^*^Correspondence should be addressed to mysun@imr.ac.cn and bijunx@uci.edu

**Content:**

Figures S1−S20

Tables S1−S7

Notes 1−6

References


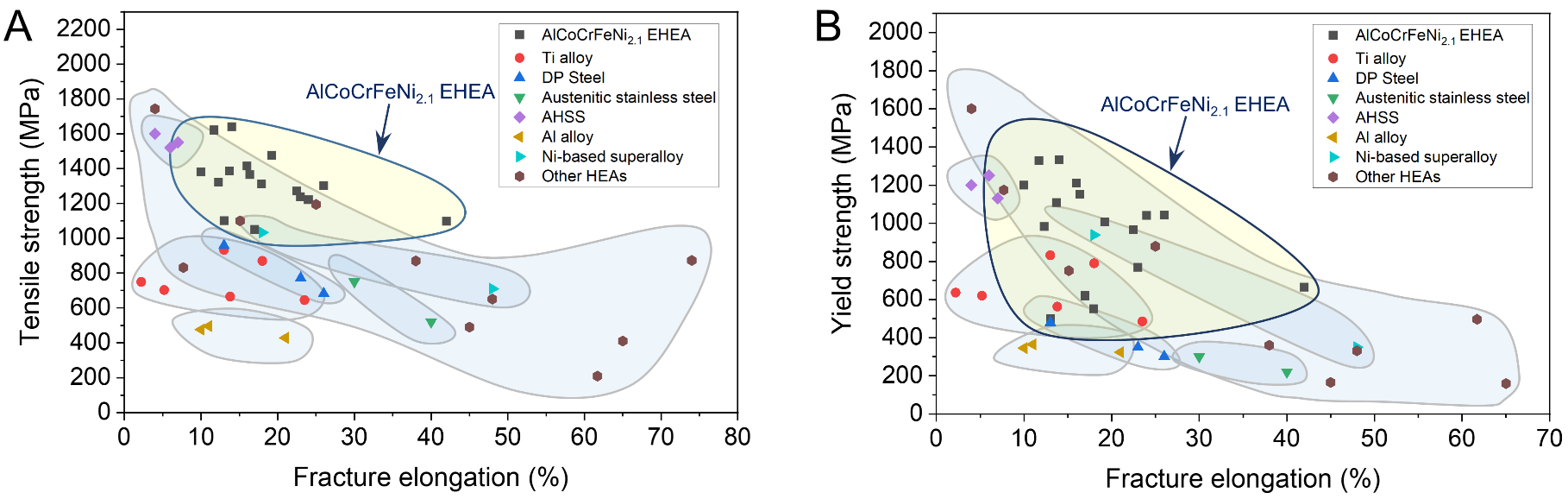


**Figure S1.** **Mechanical property comparison of the AlCoCrFeNi_2.1_ eutectic high-entropy alloy (EHEA) with other advanced engineering alloys.** Performance of the as-cast, additively manufactured, and thermomechanically processed AlCoCrFeNi_2.1_ EHEA ^[1-13]^ is benchmarked against representative Ti alloys ^[14-20]^, advanced high-strength steels (AHSS) ^[21, 22]^, dual-phase (DP) steels ^[22-25]^, Al alloys ^[26, 27]^, Ni-based superalloys ^[28, 29]^, and other HEAs ^[30-40]^. (**A**) Ultimate tensile strength versus fracture elongation. (**B**) Yield strength versus fracture elongation.

**Figure S2.** **Charpy V-notch impact energy of the AC, 600A, and 750A alloys.** All tests were performed on standard V-notch specimens with dimensions of 10 mm × 10 mm × 55 mm. The plotted values represent the average of three replicate specimens for each condition, with the error bars indicating the standard deviation.


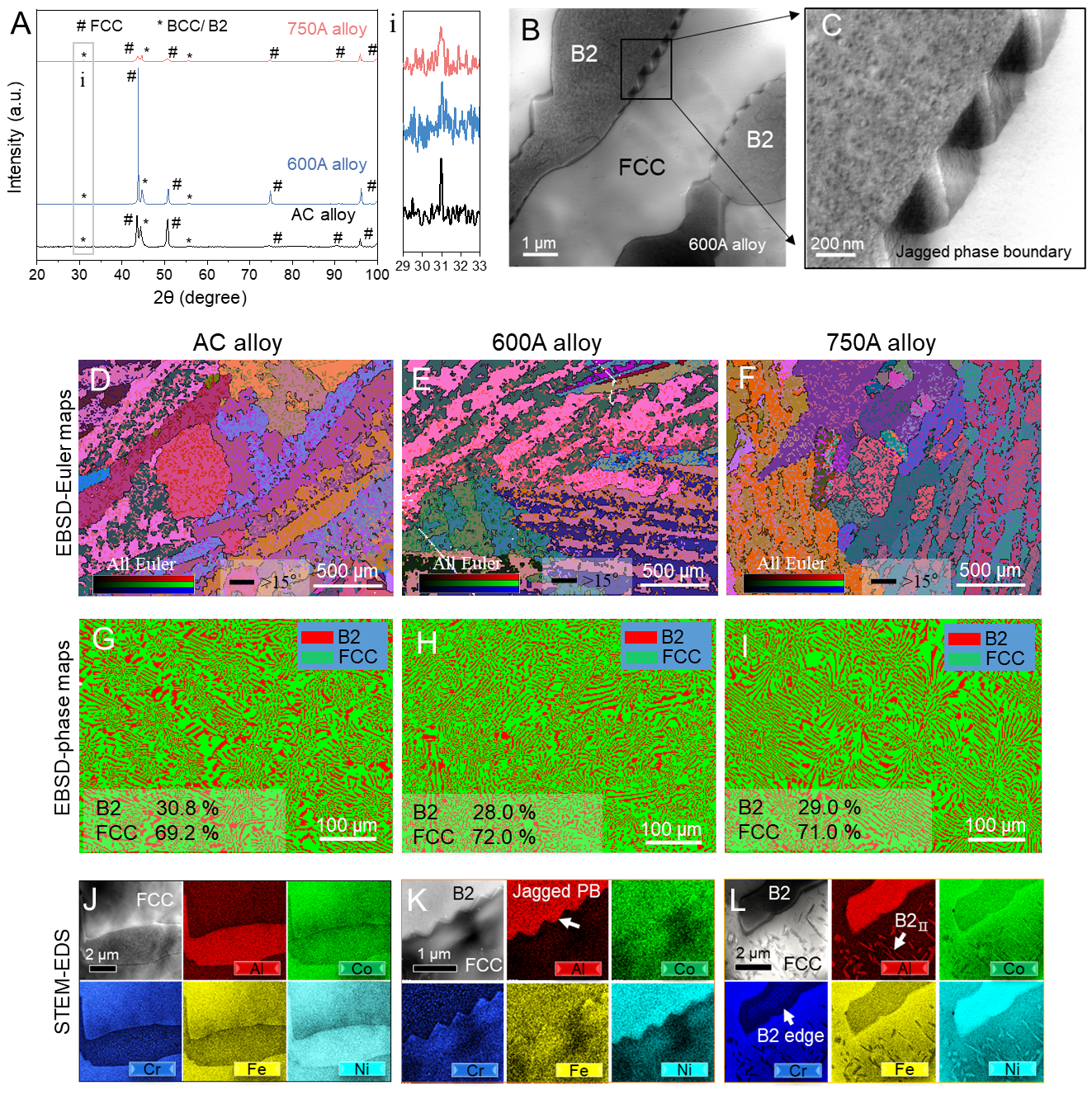


**Figure S3. Comprehensive microstructure and phase analysis of the as-cast (AC) and aged (600A, 750A) alloys.** (**A**) X-ray diffraction (XRD) patterns for the three alloys. The panel (i) highlights the (001) superlattice peak of the B2 phase. (**B**, **C**) Morphology of the jagged FCC/B2 phase boundary in the 600A alloy. (**D**–**F**) Electron backscatter diffraction (EBSD) Euler maps of the AC, 600A, and 750A alloys, respectively (step size: 10 μm). (**G**–**I**) EBSD phase distribution maps for the AC, 600A, and 750A alloys, respectively (step size: 1.81 μm). (**J**–**L**) Scanning transmission electron microscopy (STEM) energy-dispersive X-ray spectroscopy (EDS) elemental mappings for the three alloys.


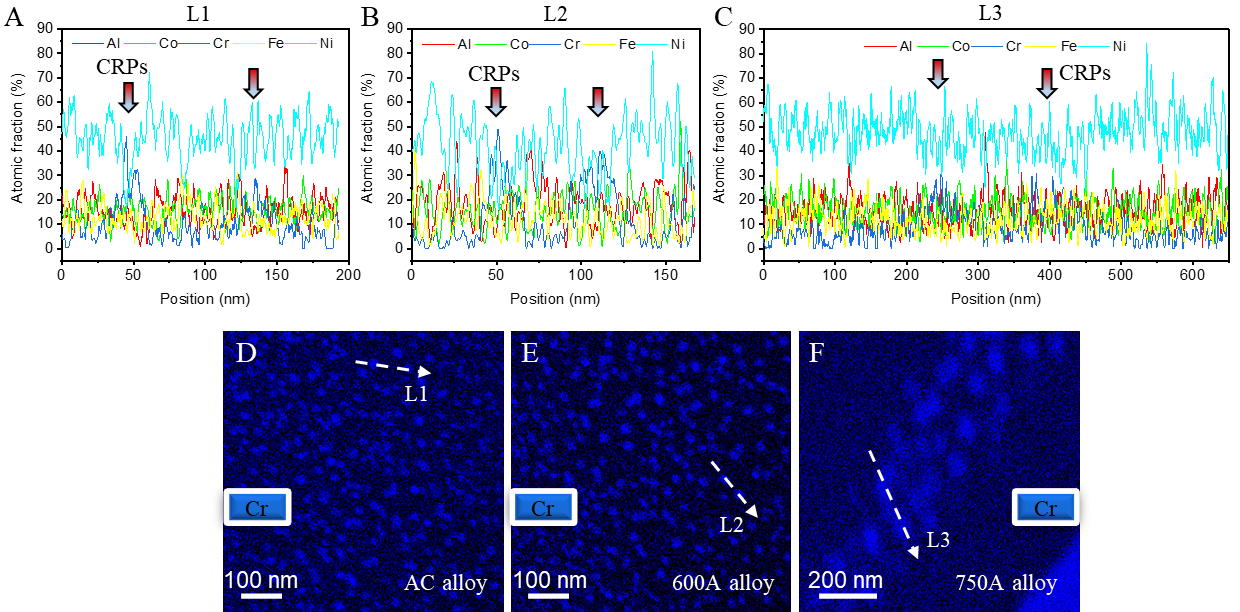


**Figure S4. STEM-EDS analysis of the CRPs within the B2 phase in the AC, 600A, and 750A alloys. (A−C)** Element line profiles of CRPs in the AC, 600A, and 750A alloys. **(D−F)** STEM-EDS mappings of Cr distribution within the B2 phase across these three alloys. The CRPs shown in (D) and (E) are taken from regions adjacent to the B2 phase border, and thus appear smaller in size compared to those in the 750A alloy.


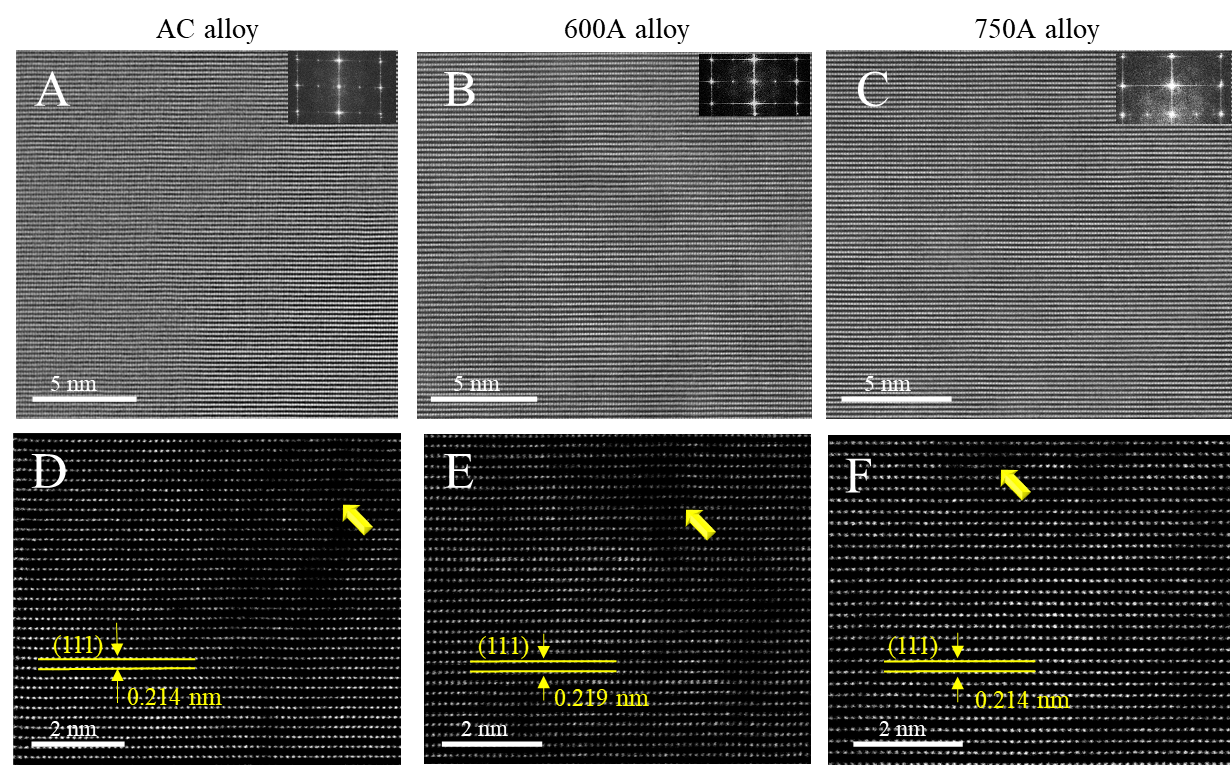


**Figure S5.** **High-resolution HAADF images of FCC and L1_2_ domains in the AC, 600A, and 750A alloys under the <112> zone axis.** (**A**) and (**D**) Atomic images of FCC and L1_2_ domains in the AC alloy. (**B**) and (**E**) Atomic images of FCC and L1_2_ domains in the 600A alloy. (**C**) and (**F**) Atomic images of FCC and L1_2_ domains in the 750A alloy.


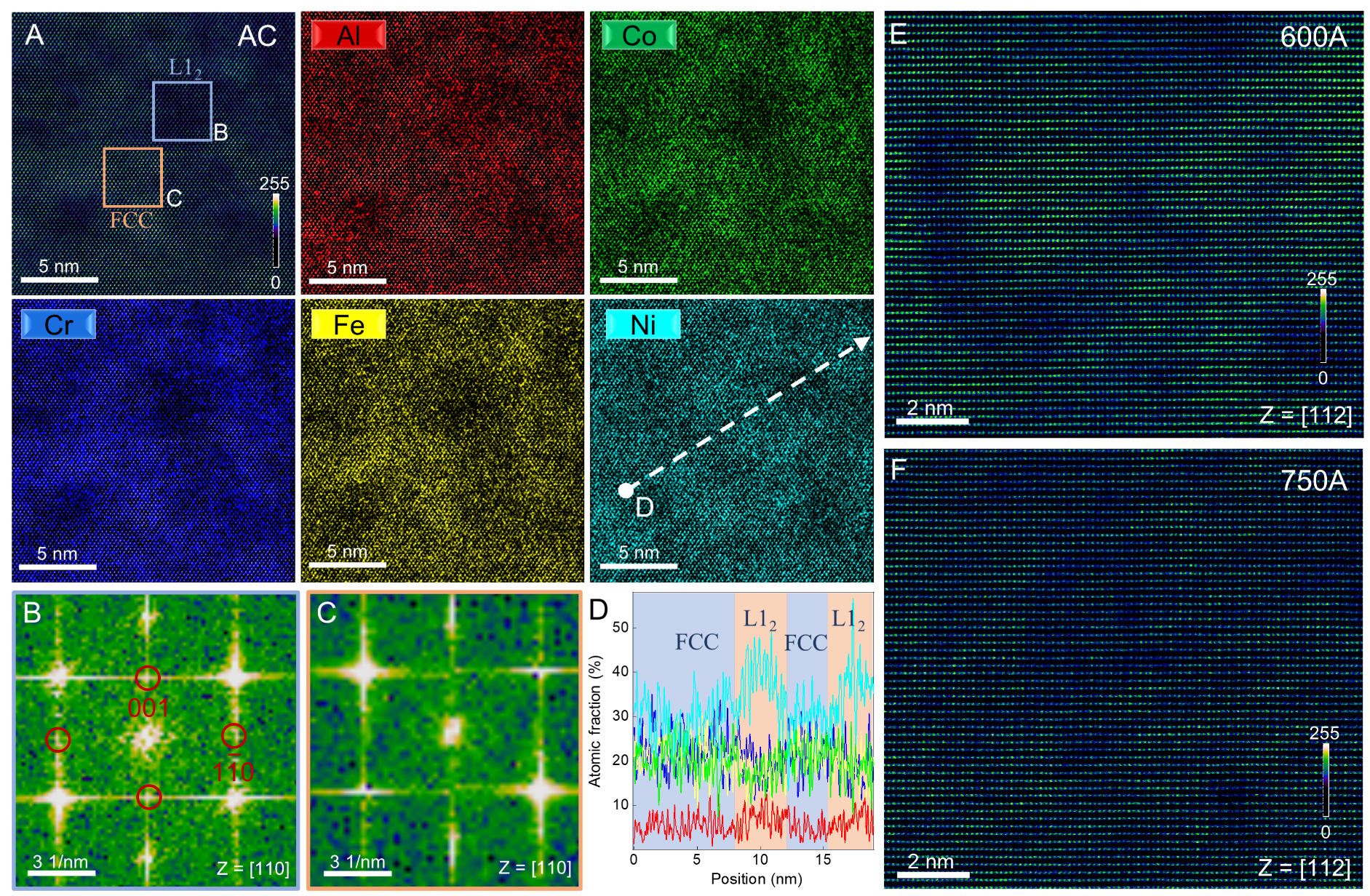


**Figure S6. High-resolution HAADF images of the L1_2_-ordered areas and FCC phase in the AC, 600A, and 750A alloys.** (**A**) Atomic-scale STEM-EDS mappings of L1_2_ domains and FCC phase in the AC alloy. The HAADF image is taken under the [110] zone axis. (**B**) FFT pattern of the region marked by the blue rectangular box in (A), showing the L1_2_ ordering feature. (**C**) FFT pattern of the region marked by the orange rectangular box in (A), showing the FCC disordering feature. (**D**) Elemental line profile of FCC and L1_2_ areas. (**E**−**F**) High-resolution HAADF images of FCC(L1_2_) matrix in the 600A and 750A alloys, showing the significant elemental heterogeneity at the atomic scale. It indicates the significant local chemical order. Nevertheless, it is important to note that these compositional and ordering distributions exhibit significant local fluctuations.


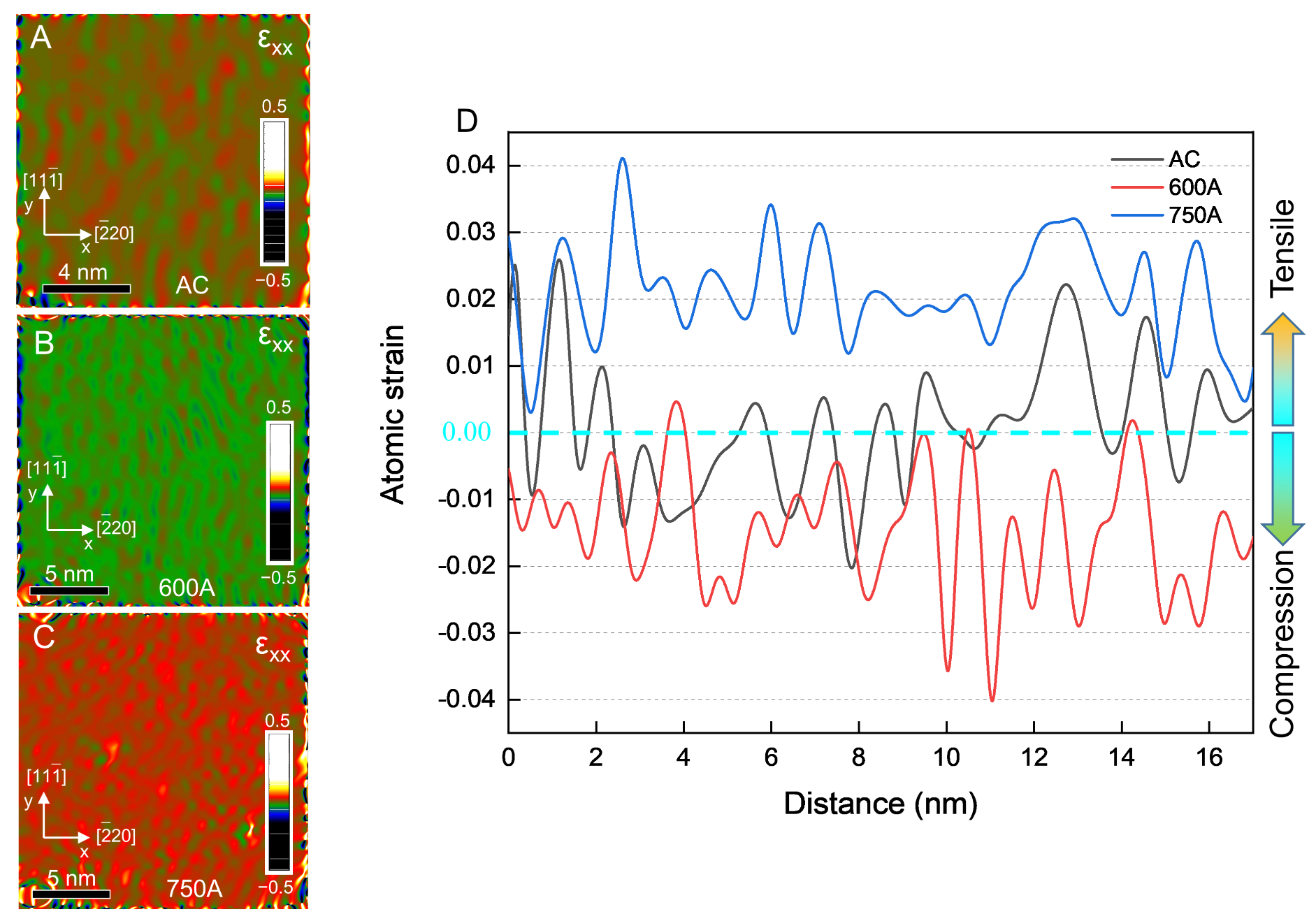


**Figure S7. Atomic elastic strain of FCC(L1_2_) matrix in the AC, 600A, and 750A alloys**. (**A**−**C**) Atomic elastic strain mappings of FCC(L1_2_) matrix in the AC, 600A, and 750A alloys, respectively. (**D**) Atomic elastic strain distribution curves of the FCC(L1_2_) matrix in these three alloys. The FCC(L1_2_) matrix in the 600A alloy exhibits a compressive atomic elastic strain, whereas that in the AC and 750A alloys displays fluctuating tensile-compression and tensile elastic strain, respectively. These findings suggest that the FCC(L1_2_) matrices in these three alloys experience varying atomic elastic strains, which will impact the Peierls-Nabarro stress in the lattice and subsequently influence the slip behavior of dislocations.


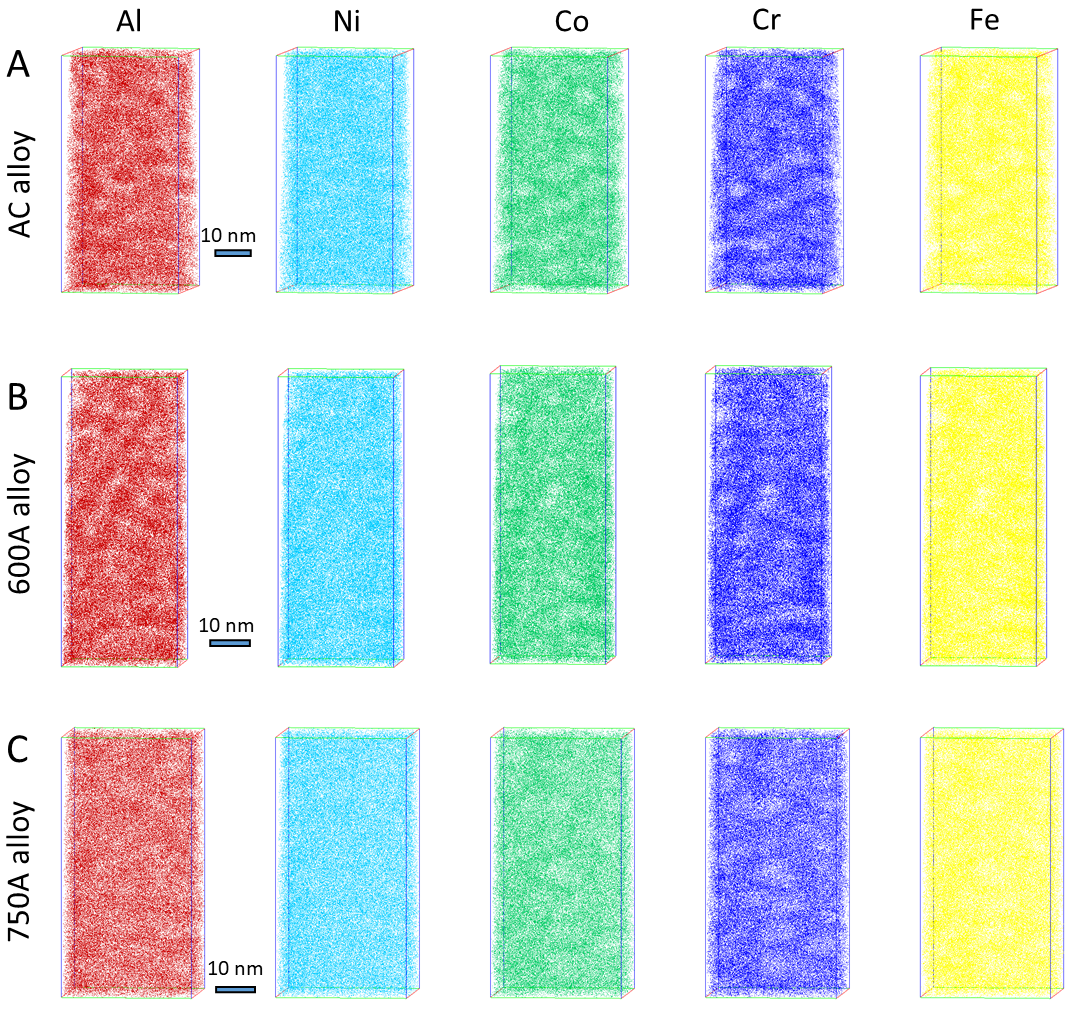


**Figure S8.** Elaborated atomic mappings of L1_2_ domains in three alloys characterized by atom probe tomography (APT), showing evident Fe-Co-Cr and Ni-Al enriched regions in three alloys.


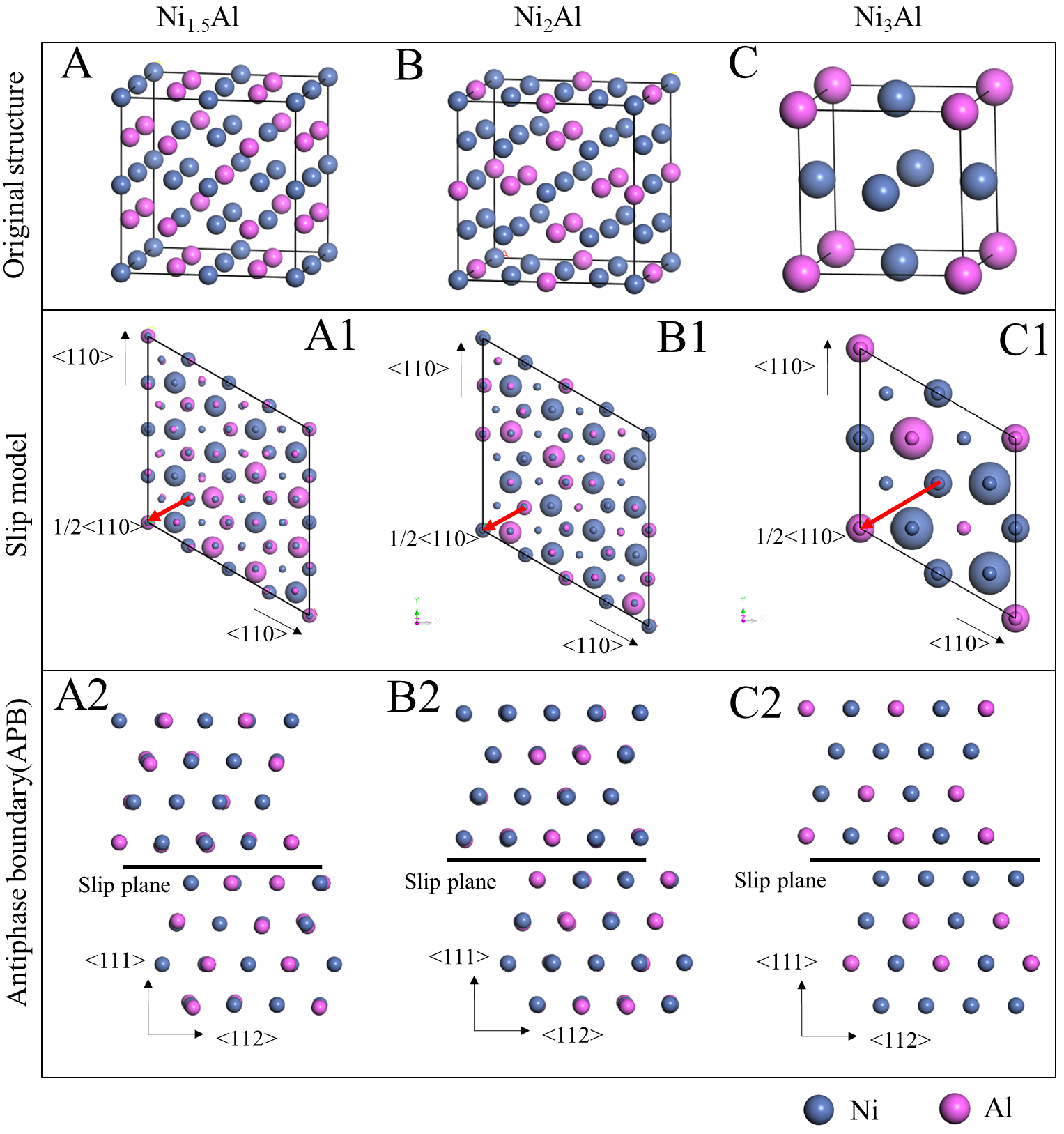


**Figure S9. DFT calculation of shear modulus and APBEs for the Ni_1.5_Al, Ni_2_Al, and Ni_3_Al alloys.** (**A**−**A2**) The constructed structure, slip model, and APB for the Ni_1.5_Al alloy, respectively. (**B**−**B2**) The constructed structure, slip model, and APB for the Ni_2_Al alloy, respectively. (**C**−**C2**) The constructed structure, slip model, and APB for the Ni_3_Al alloy, respectively. In the study of the Ni_1.5_Al and Ni_2_Al structures, we employ 2 × 2 × 2 supercells, each containing 32 atoms. The APB is generated by upper and lower parts sliding by a 1/2<110> slip vector, as shown in (A1), (B1), and (C1).


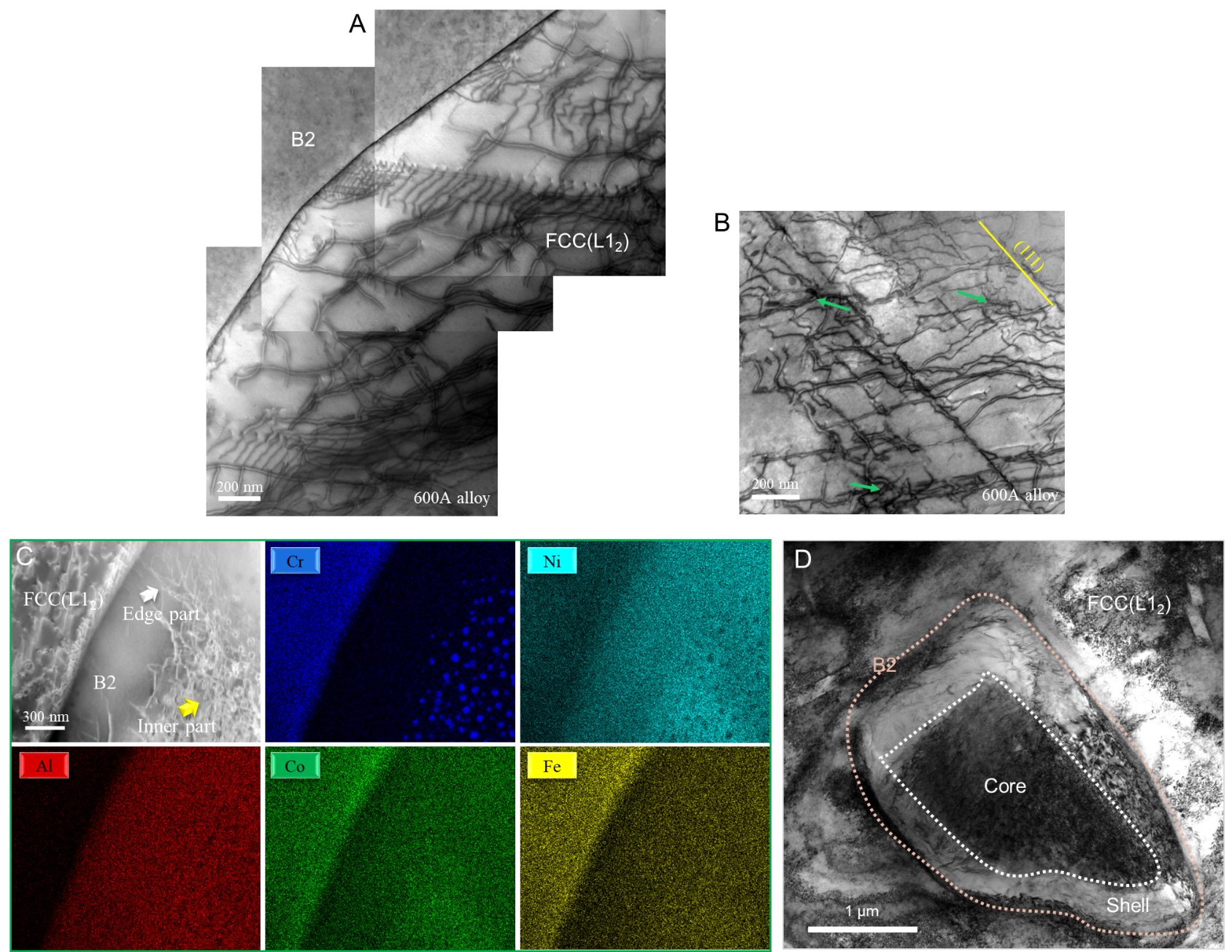


**Figure S10.** **Dislocation analysis and deformation mechanisms in the 600A and 750A alloys. 600A Alloy:** (**A**, **B**) Bright-field transmission electron microscopy (BF-TEM) images showing complex and entangled dislocation networks within the FCC(L1_2_) matrix.

**750A Alloy:** (**C**) BF-STEM image and corresponding STEM-EDS mappings of the B2 phase edge. The white arrow indicates the dislocations traversing the Cr-rich particle (CRP)-free zone, while the yellow arrow highlights the significant dislocation tangle within the phase interior. (**D**) BF-TEM image revealing a shell-core dislocation configuration within the B2 phase.


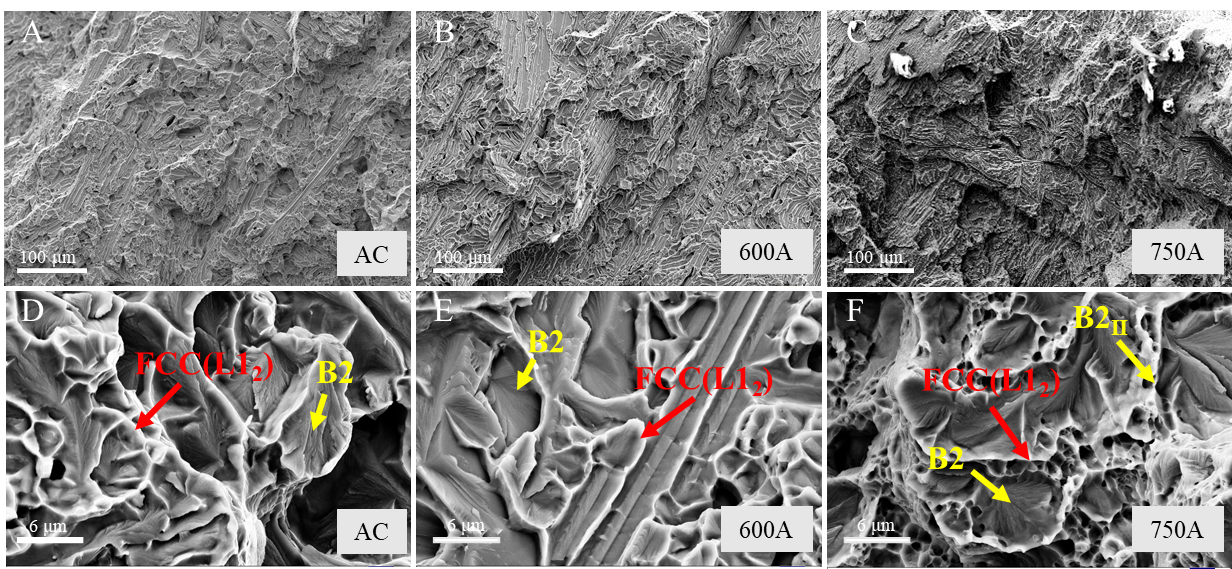


**Figure S11. Fracture surface analysis of the as-cast (AC), 600A, and 750A alloys.** (**A**–**C**) Overall fracture morphologies of the AC, 600A, and 750A alloys, respectively. (**D**–**F**) Corresponding higher magnification images showing detailed fracture features for each alloy.


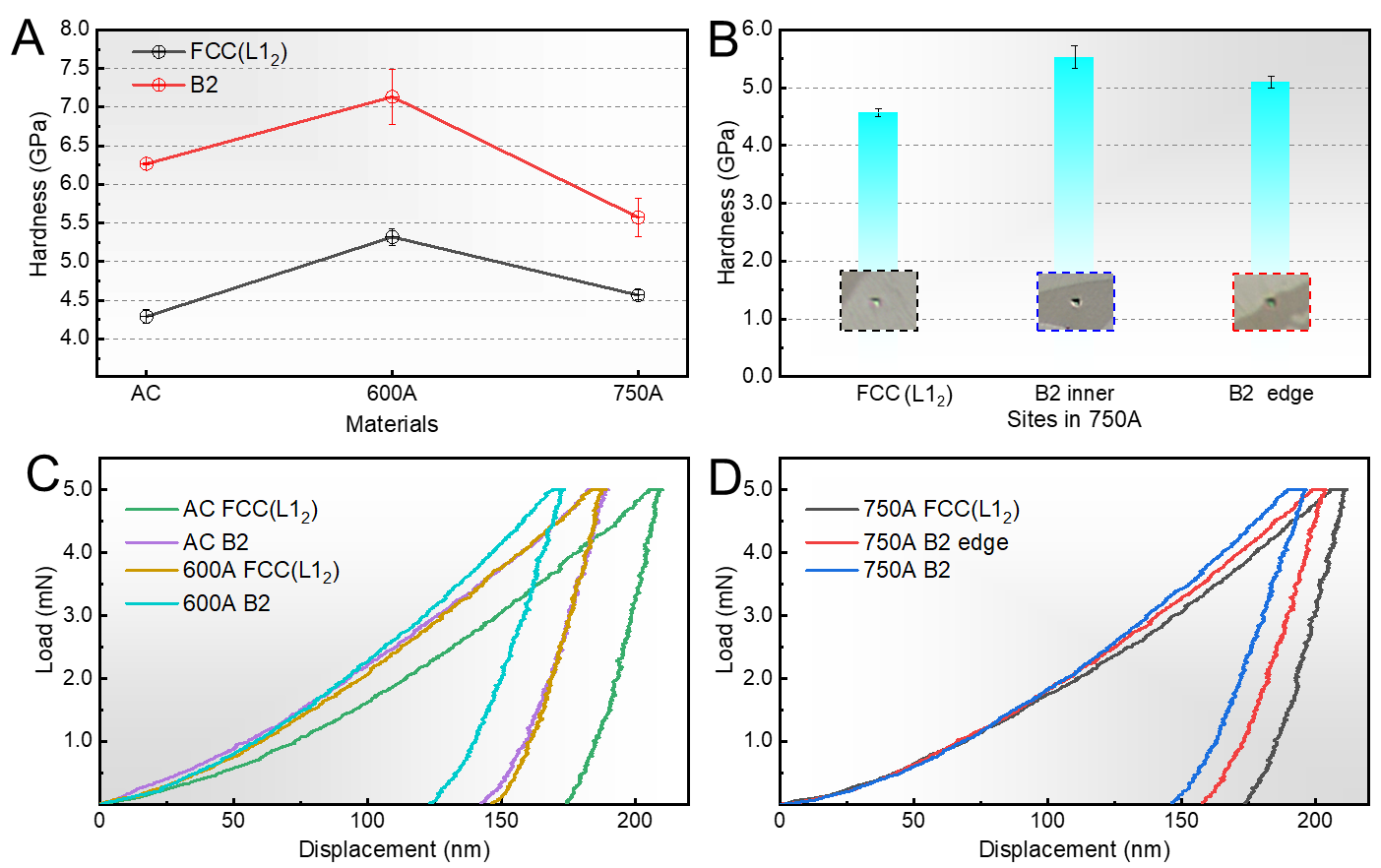


**Figure S12. Nanoindentation results for the as-cast (AC), 600A, and 750A alloys.** (**A**) Hardness of the FCC(L1_2_) matrix and B2 phase in the three alloys. (**B**) Hardness comparison of the FCC(L1_2_) matrix, B2 phase interior (B2 inner), and B2 phase edge in the 750A alloy. The insets are SEM images showing the corresponding indentation locations. (**C**) Representative load-displacement curves for the FCC(L1_2_) and B2 phases in the AC and 600A alloys. (**D**) Representative load-displacement curves for the FCC(L1_2_) matrix, B2 phase interior, and B2 phase edge in the 750A alloy.


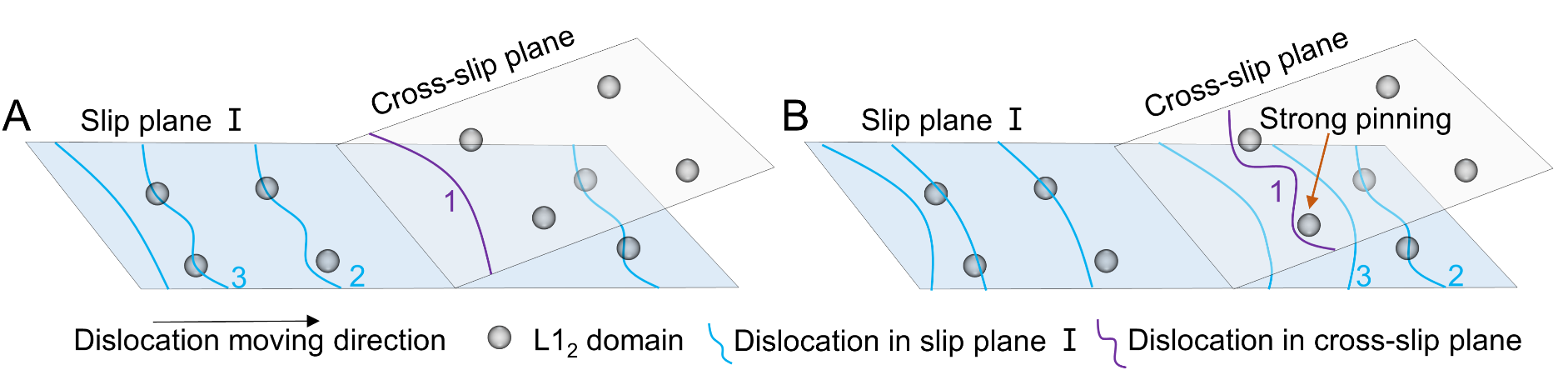


**Figure S13. Schematic diagram of the influence of ‘glide plane softening’ effect on the movement of dislocation in FCC(L1_2_) matrix in the 600A alloy.** The deformation localization observed in the 600A alloy can be attributed to a ‘glide plane softening’ effect within the FCC(L1_2_) matrix ^[41]^. This phenomenon arises because the passage of the first dislocation through the ordered L1_2_ domains disrupts the local chemical order. Consequently, the slip resistance for this leading dislocation is substantially higher than for subsequent dislocations moving on the same, now-disordered plane. When a dislocation source is activated, the pile-up of subsequent dislocations exerts a forward pressure on the leading dislocation, aiding it in overcoming the initial high slip resistance. This cooperative process promotes the rapid movement of a large sequence of dislocations on a single slip plane, resulting in highly localized planar slip. This deformation localization is intensified when the degree of L1_2_ ordering increases, primarily due to the strong inhibition of cross-slip, as schematically illustrated in Figure S13. A higher degree of ordering corresponds to a higher antiphase boundary energy (APBE), which raises the energy barrier for dislocations to shear through the L1_2_ domains. This has two main consequences. First, the activation of new slip systems becomes more difficult. Second, and more critically, it suppresses dislocation cross-slip. As depicted in the model (Figure S13A), dislocations are initially activated on the primary slip plane I, which experiences the highest resolved shear stress. As stress increases, cross-slip onto a secondary plane becomes theoretically possible. However, shearing the highly-ordered L1_2_ domains presents a significant energy barrier, effectively pinning the dislocation and hindering this mechanism. Unable to escape via cross-slip, subsequent dislocations are confined to the original, softened plane I. This confinement leads to an intensification of planar slip and more severe strain localization (Figure S13B). Therefore, a higher APBE in the L1_2_ domains exacerbates planar slip and promotes more pronounced deformation localization.


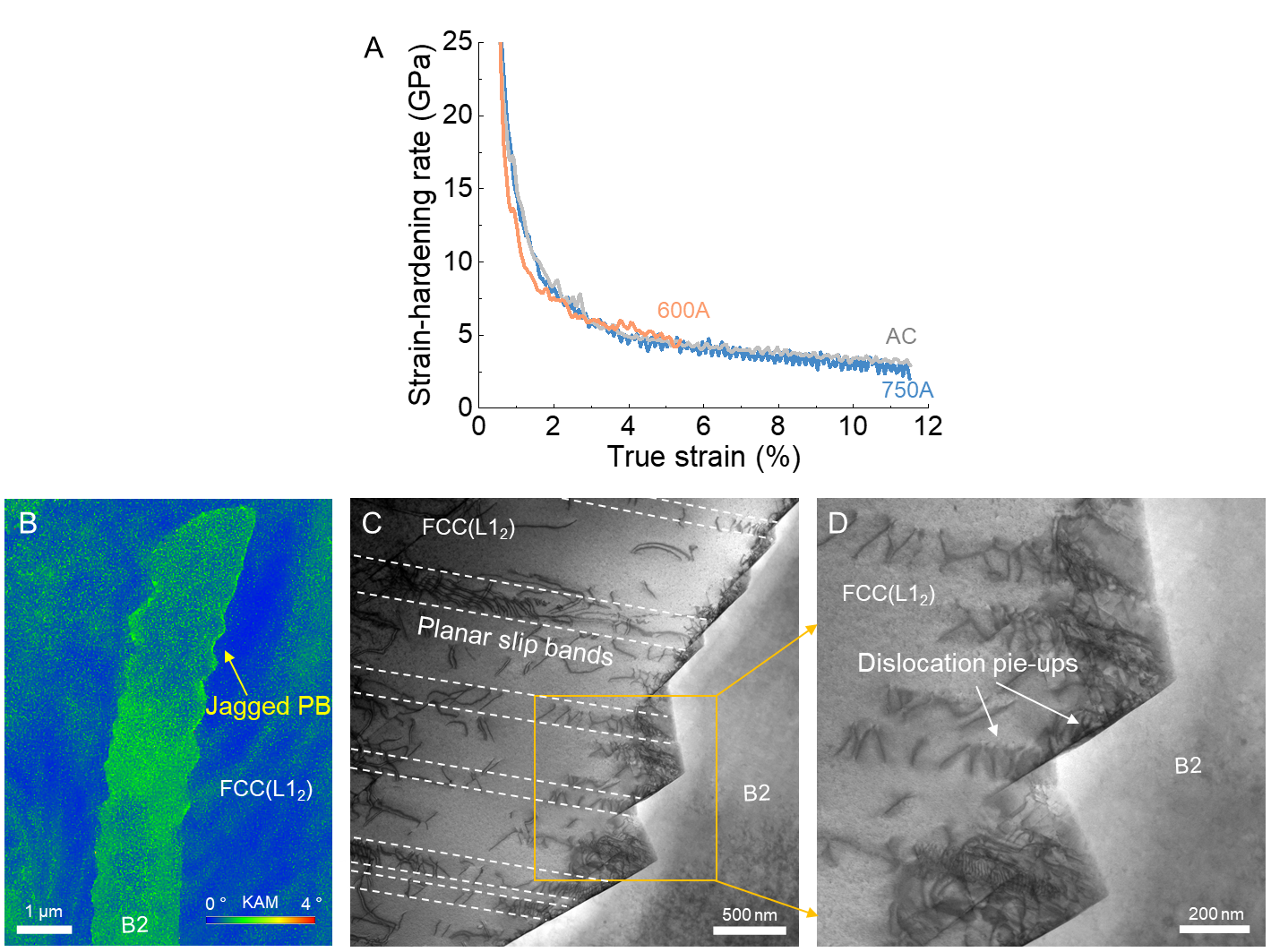


**Figure S14. Strain-hardening behavior and associated deformation mechanisms.** (**A**) Strain-hardening rate versus true strain curves for the AC, 600A, and 750A alloys. (**B**−**D**) Analysis of the deformation behavior at the jagged B2/FCC(L1_2_) phase boundaries (PBs) in the 600A alloy: (B) transmission Kikuchi diffraction (TKD) Kernel average misorientation (KAM) map showing local misorientation. (C) Bright-field scanning transmission electron microscopy (BF-STEM) image of dislocation pile-ups at a jagged PB, and (D) a corresponding higher magnification view.


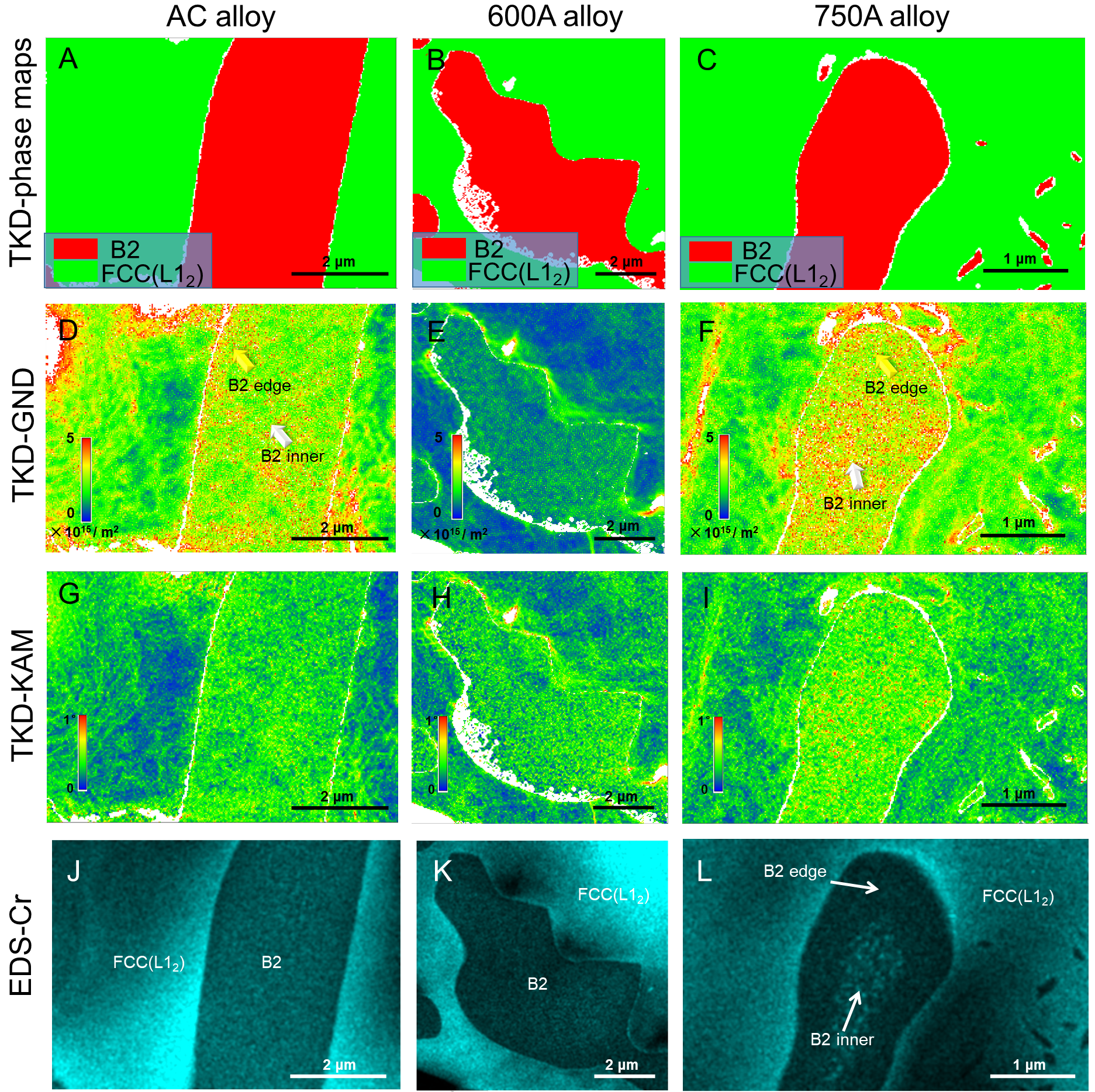


**Figure S15. Transmission Kikuchi diffraction (TKD) analysis of deformation mechanisms in the as-cast (AC), 600A, and 750A alloys.** (**A**–**C**) Phase distribution maps. (**D**–**F**) Geometrically necessary dislocation (GND) density maps. (**G**–**I**) Kernel average misorientation (KAM) maps showing local strain distribution. (**J**–**L**) Corresponding Cr elemental distribution maps for the regions shown in (A–C). The TKD step sizes are 0.013 μm (AC), 0.035 μm (600A), and 0.015 μm (750A). The white regions in the TKD results represent unindexed areas. These are attributed to the imperfect Kikuchi diffraction signal arising from the uneven thickness transition across the phase boundary (PB) between the two phases. Given their small size and localized nature, these unindexed regions do not impact the overall microstructural trends or compromise the validity of our conclusions.

**Tables**

**Table S1.** Composition (at%) of L1_2_ domains in the AC, 600A, and 750A alloys obtained by APT.

|  | Ni | Al | Fe | Co | Cr |
| --- | --- | --- | --- | --- | --- |
| AC | 59.5±1.8 | 33.6±3.6 | 2.0±0.1 | 4.3±0.2 | 0.6±0.2 |
| 600A | 60.9±4.2 | 21.8±2.1 | 5.7±0.9 | 7.7±1.2 | 3.9±1.9 |
| 750A | 58.4±2.63 | 31.2±0.9 | 3.3±0.2 | 4.9±0.1 | 2.2±0.1 |

**Table S2. Shear modulus and APBEs calculated by DFT.** Doping 1 and 2 are conducted by substituting Ni with Fe, Co, and Cr, where doping 1 replaces parts of Ni with the Fe, Co, and Cr components of AC alloy, and doping 2 replaces parts of Ni with the Fe, Co, and Cr components of 600A alloy. It can be observed that a higher Fe, Co, and Cr doping (doping 2) leads to a slight increase in APBE.

| Alloys | Shear modulus (GPa) | APBE (mJ/m^2^) |
| --- | --- | --- |
| Ni_1.5_Al | 70.02 | 60.31 |
| Ni_2_Al | 79.26 | 38.29 |
| Ni_3_Al | 99.06 | 244.65 |
| Ni_3_Al (doping 1) | 101.40 | 222.46 |
| Ni_3_Al (doping 2) | 98.81 | 266.54 |

**Table S3.** A collection of reported thermal-induced embrittlement in typical conventional alloys.

| **Alloys** | **Embrittlement Type** | **Embrittlement Mechanism** | **Embrittling Temperature** | **Fracture Model** |
| --- | --- | --- | --- | --- |
| Ni and Ni-based alloys ^[42-49]^ | ITE | DSA; IP; DGP;  GBS (S, Bi) | 600−900 °C | IGF` |
| Low-alloy steels  ^[50, 51]^ | TE | GBS (P, S, Sb, Sn, and As) | 300−600 °C | IGF |
| Austenitic stainless steels ^[50]^ | ICE | IP; GBS (P, S) | 450−900 °C | IGF |
| Fe-Cr duplex phase steels ^[52, 53]^ | Aging embrittlement | Phase decomposition and the G-phase precipitation | 280−550 °C | Cleavage fracture |
| Al-Li alloys ^[54]^ | Aging embrittlement | IP; PFZ adjacent to GB;  GBS (Li, Na) | Natural aging and artificial aging | IGF |

ITE: intermediate temperature embrittlement; ICE: intergranular corrosion embrittlement; TE: temper embrittlement; DSA: dynamic strain aging; IP: intergranular precipitates; GB: grain boundary; GBS: grain boundary segregation; DGP: decohesion of glide plane; IGF: intergranular fracture; PFZ: precipitate-free zone. In Ni-based superalloys, intermediate-temperature embrittlement primarily arises from grain boundary (GB) segregation of S and Bi elements and the formation of brittle precipitates along GBs. Low-alloy steels exhibit temper embrittlement due to impurity segregation (notably P, S, Sb) at GBs during intermediate-temperature tempering. Austenitic stainless steels experience embrittlement due to large precipitate formation at GBs during sensitization, often accompanied by GB oxidation, which is exacerbated by S and P. Aging embrittlement in Fe-Cr duplex phase steels results from phase decomposition and interfacial precipitation of brittle G-phase. In Al-Li alloys, aging brittleness is predominantly caused by GB segregation of Li and Na, precipitation of intergranular brittle phases, and the formation of precipitate-free zones (PFZ) adjacent to GBs.

**Table S4.** A collection of loading methods, mechanical properties, and possible aging treatments and corresponding influence of reported eutectic HEAs (N/A-not available).

| **Eutectic HEAs** | **Loading** | **Fracture Strength(σ_b_) and Strain(ε), Yield Strength(σ_0.2_)** | **Aging Treatment** | **Effect of Aging on Properties** |
| --- | --- | --- | --- | --- |
| AlCoCrFeNi_2.1_ ^[2, 55-60]^ | Tensile | σ_0.2_ = 480−500 MPa  σ_b_ =1000−1105 MPa  ε= 13%−17% | This work | |
|  |  | σ_b_ = 906 MPa,  ε = 30.7%  (Test at 600 °C) |  |  |
|  |  | σ_b_= 538 MPa,  ε = 22.9%  (Test at 700 °C) |  |  |
| Al_17_Co_14.3_Cr_14.3_Fe_14.3_Ni_40.1_ ^[61]^ | Tensile | σ_0.2_ = 479 MPa,  σ_b_ = 1067 MPa,  ε = 14% | N/A | N/A |
| Al_20.45_Co_10_Cr_10_Ni_59.55_ ^[62]^ | Tensile | σ_0.2_ = 710 MPa,  σ_b_ = 718 MPa,  ε = 1.85% | N/A | N/A |
| Al_19.3_Co_15_Cr_15_Ni_50.7_ ^[62]^ | Tensile | σ_0.2_ = 699 MPa,  σ_b_ = 1227MPa,  ε = 10.3% | N/A | N/A |
| Fe_28.2_Ni_18.8_Mn_32.9_Al_14.1_Cr_6_ ^[63]^ | Tensile | σ_0.2_ = 679 MPa,  σ_b_ = 931 MPa,  ε = 17.8% | N/A | N/A |
| Al_0.8_CoCr_0.6_Fe_0.7_Ni_1.5_ ^[64]^ | Tensile | σ_0.2_ = 490 MPa,  σ_b_ = 980 MPa,  ε = 14.8% | N/A | N/A |
| CrFeNi_2.2_Al_0.8_ ^[65]^ | Tensile | σ_0.2_ = 479 MPa,  σ_b_ = 956 MPa,  ε = 12.7% | N/A | N/A |
| Al_0.7_CoCrFeNi_2.4_ ^[66]^ | Tensile | σ_0.2_ = 957 MPa,  σ_b_ = 1102 MPa,  ε = 17.9% | Aging from 630 °C to 910 °C | **Aging decreases ductility/plasticity** |
| CoCrFeNi_2.1_Nb_0.4_ ^[67]^ | Tensile | σ_0.2_ = 628 MPa,  σ_b_ = 814 MPa,  ε = 6% | Aging at 800 ℃ for 24h |  |
| AlCrFeNiMo_0.2_ ^[68, 69]^ | Compression | σ_0.2_ = 1487 MPa,  σ_b_ = 3222 MPa,  ε = 28.7% | Annealing from 750 to 950 °C for 6h |  |
| CoFeNi_2_V_0.5_Nb_0.75_ ^[70, 71]^ | Compression | σ_0.2_ = 2073 MPa,  σ_b_ = 2232 MPa,  ε = 3.4% | Aging from 500 to 1000 °C for 6h |  |
| Fe_35_Ni_25_Cr_25_Mo_15_ ^[72, 73]^ | Compression | σ_0.2_ = 1065 MPa,  σ_b_ = 1875 MPa,  ε = 3.7% | Aging at 600 °C for 24h |  |
| CoCrFeNiTa_0.4_ ^[74]^ | Compression | σ_0.2_ = 1316 MPa,  σ_b_ = 2293 MPa,  ε = 22.6% | N/A | N/A |
| Co_2_Mo_0.8_Ni_2_VW_0.8_ ^[75]^ | Compression | σ_0.2_ = 1431 MPa,  σ_b_ = 2364 MPa,  ε = 14.4% | N/A | N/A |
| (CoFe_2_NiV_0.5_Mo_0.2_ )_91_Nb_9_ ^[76]^ | Compression | σ_0.2_= 1449 MPa,  σ_b_ = 2191.7 MPa,  ε = 19.4% | N/A | N/A |
| CoCrFeNiMo_0.8_ ^[77]^ | Compression | σ_0.2_ = 2260 MPa,  σ_b_ = 2219 MPa,  ε = 6.8% | N/A | N/A |
| AlCoCrFeNiNb_x_ ^[78]^ | Compression | σ_0.2_ = 2473 MPa,  σ_b_ = 3170 MPa,  ε = 4.1% | N/A | N/A |
| AlCoCrCuFeNiMo_0.4_ ^[79]^ | Compression | σ_0.2_ = 1690 MPa,  σ_b_ = 2660 MPa,  ε= 1.5% | N/A | N/A |
| AlCrFeNiTi_0.25_ ^[80]^ | Compression | σ_0.2_ = 1445 MPa,  σ_b_ = 1829.1 MPa,  ε = 7.2% | N/A | N/A |

**Notes**

**Note 1: Modulus and ordering strengthening of L1_2_ domains on the FCC(L1_2_) matrix.** For modulus hardening, the stress increment can be described by the Knowles–Kelly equation ^[81]^

$\Delta\sigma_{modulus}=\frac{M\Delta G}{4\pi^{2}}(\frac{3\Delta G}{Gb})^{\frac{1}{2}}[0.8-0.143ln(\frac{r}{b})]^{2/3}r^{\frac{1}{2}}f^{\frac{1}{2}}$ (1)

Where M is the Taylor factor, b is the Burgers vector of the dislocation, r is the mean radius of the L1_2_ domains, and G is the modulus of the matrix. ΔG is the difference in the shear modulus between the matrix and the L1_2_ domain. f is the volume fraction of the L1_2_ domain, where n is the number density of the L1_2_ domains.

For ordering hardening, the stress increment can be estimated by ^[82]^

$\Delta\sigma_{ordering}=\frac{M\gamma_{apb}^{\frac{2}{3}}}{b}(\frac{4r_{s}f}{\pi T_{l}})^{\frac{1}{2}}$ (2) Where r_s_=(2/3)^1/2^r is the average radius of the sheared particles in the gliding plane, γ_apb_ is the average value of the antiphase boundary energy (APBE) for L1_2_ domains, and T_l_ is the dislocation line tension. Due to the complex phase constituent of this multiphase HEA, the strength contribution of modulus and ordering strengthening from L1_2_ domains is hard to quantifiably calculate. However, it is helpful to understand their contributions qualitatively.

Therefore, the increased modulus and APBE of L1_2_ domains in the 600A alloy enhance the modulus and ordering strengthening. Similarly, the increased size of L1_2_ domains in the 750A alloy enhances the modulus and ordering strengthening.

**Note 2: Comparative analysis with the conventional eutectic alloy system.** To contextualize the unique mechanism observed in our high-entropy alloy (HEA), we performed a comparative study on a conventional Al-33wt% Cu eutectic alloy. Conventional eutectic systems are typically characterized by pronounced brittleness, as one of the constituent phases often provides a preferential path for crack initiation and propagation. Thermal aging normally exacerbates this fragility by coarsening the microstructure, leading to further ductility loss. A comprehensive literature survey (Table S6), combined with our direct experiments, reveals no evidence of a synergistic embrittlement mechanism analogous to the interplay between phase boundaries (PBs) and local chemical ordering (LCO) reported in our work.

Our experimental investigation of the Al-Cu system (Table S5) confirms this distinction. The as-cast alloy possesses negligible tensile ductility (<1%), and subsequent aging treatments, despite inducing the formation of Guinier-Preston (G.P.) zones, conferred no improvement in ductility. Most importantly, detailed microstructural analysis demonstrated that the eutectic lamellar interfaces remained morphologically unchanged throughout the aging process (Figure S16).

These findings provide compelling evidence that the synergistic PB-LCO embrittlement is not a feature of conventional binary eutectic alloys. We attribute this behavior to the inherent compositional complexity of HEAs. Their multi-principal-element nature establishes a unique thermodynamic and kinetic landscape that enables intricate interfacial phenomena, such as elemental partitioning and ordering, which are inaccessible in simpler binary systems.

**Table S5**．**Tensile mechanical properties of as-cast (AC) and aged Al-33wt% Cu eutectic alloys.** The alloys were aged under three conditions: 110 °C for 12 h (A110), 160 °C for 2 h (A160), and 200 °C for 0.5 h (A200), followed by air cooling. The microstructural characteristics corresponding to these heat treatments are detailed elsewhere ^[83, 84]^.

| **Alloys** | **Fracture strength (MPa)** | **Yield strength (MPa)** | **Elongation (%)** | **Microstructure** |
| --- | --- | --- | --- | --- |
| AC | 256±22 | 218±25 | 0.5±0.5 | α, θ |
| A110 | 232±18 | 224±5 | 0.7±0.3 | α, θ, G.P. Zone |
| A160 | 237±21 | 222±30 | 0.5 | α, θ, G.P. Zone, θ' |
| A200 | 241±15 | 212±6 | 0.5 | α, θ, θ" |


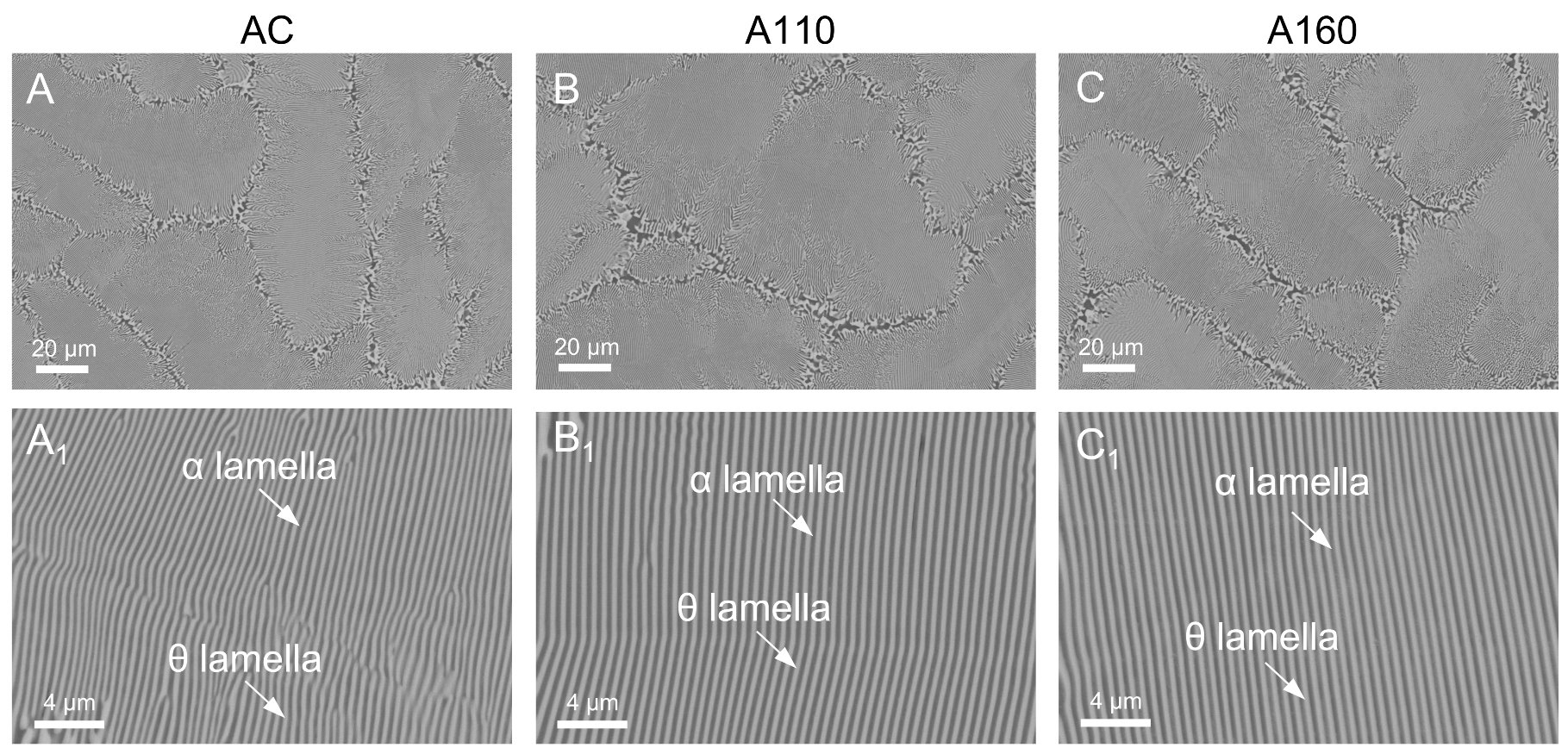


**Figure S16.** **Microstructural evolution of the Al-33wt% Cu eutectic alloy during aging.** (**A**, **A_1_**) Microstructure of the as-cast (AC) alloy. (**B**, **B_1_**) Microstructure after aging at 110 °C for 12 h. (**C**, **C_1_**) Microstructure after aging at 160 °C for 2 h. (A_1_–C_1_) are higher magnification images corresponding to images (A–C), respectively.

**Table S6.** A collection of eutectic temperature, mechanical properties, and possible aging treatment and corresponding influence of reported conventional binary eutectic alloys (N/A-not available).

| **Conventional Binary Eutectic Alloys** | **Eutectic Temperature** | **Fracture Strength(σ_b_) and Strain(ε)** | **Aging Treatment** | **Effect of Aging Treatment on Properties** |
| --- | --- | --- | --- | --- |
| Al-12wt% Si  ^[85-87]^ | ~ 577 °C | ε~5%  σ_b_~150 MPa | Not heat-treatable | Aging coarsens Si and reduces ductility |
| Al-33wt% Cu  ^[83, 84]^ | ~ 548 °C | ε~0.5%  σ_b_~228 MPa | At 110 °C, G.P. Zone forms;  At 160 °C, G.P. Zone and θ' form;  At 180 °C, θ' and θ'' form | Aging treatment is ineffective |
| Pb-62wt% Sn (Solder) ^[88]^ | ~ 183 °C | ε~40%  σ_b_~50 MPa | Natural aging and artificial aging at 100 °C | Aging treatments increase the ductility |
| Zn-5wt% Al  ^[89, 90]^ | ~ 381 °C | ε~6%  σ_b_~141 MPa | Natural and artificial aging | Aging treatments increase the ductility |
| Ti-8.5wt% Si ^[91]^ | ~ 1350 °C | ε < 1%  σ_b_~759 MPa | N/A | N/A |
| Mg-32wt% Al ^[92]^ | ~ 437 °C | ε ~ 0%  σ_b_ < 60 MPa | N/A | N/A |
| Fe-4.3wt% C | ~ 1148 °C | ε ~ 0%  Very brittle | N/A | N/A |

**Note 3: Formation mechanism of jagged B2/FCC PB in the 600A alloy.** As shown in Figure S17, the jagged B2/FCC(L1_2_) PBs consist of zig-zag interface Ⅰ and interface Ⅱ with different orientations as indicated in Figure S17A. Interface Ⅱ corresponds to the coherent, relatively smooth interfaces preserved from the as-cast state after aging. It is indexed as ($\bar{2}\bar{1}1$)_FCC_//($\bar{2}1\bar{3}$)_B2_, featuring high coherency and a large slip-transfer geometric factor that enables dislocation transfer and confers ductility ^[93]^. By contrast, Interface Ⅰ, indexed as ($\bar{7}5\bar{5}$)_FCC_//($23\bar{1}$)_B2_, emerges during aging and exhibits a more complex and defective structure, characterized by lower coherency and a higher interfacial energy. Therefore, the formation of the jagged PB arises from the structural evolution of initially straight, unstable PBs into an energetically favored configuration comprising alternating facets of interfaces I and II during aging. This process can be rationalized by considering both thermodynamic and kinetic factors:

**Thermodynamic rationale:** The initially as-cast PB is chemically and structurally heterogeneous, possessing a high interfacial energy. During aging at 600 °C, the system seeks to lower its free energy through interface reconstruction. This results in the formation of zig-zag or serrated PB morphology, consistent with Wulff-type selection ^[94]^, where interfacial segments reorient to minimize the total energy by balancing interfacial energy anisotropy and coherency strain relaxation. Although introducing interface Ⅰ locally increases interfacial energy, the net free-energy reduction from chemical partitioning and strain relaxation can favor the mixed-facet (jagged) configuration.

**Kinetic rationale.** The temperature-dependent diffusivity controls the mode of interface migration. At 600 °C, bulk diffusion is sluggish, while interfacial diffusion dominates. Under such conditions, phase boundary transition switches from a continuous to discontinuous evolution mechanism, which is analogous to the formation of faceted fronts in discontinuous precipitation reactions ^[95]^. Thus, the phase boundary itself migrates in a serial, facet-by-facet manner, through short-range interfacial diffusion, which naturally produces a serrated or scalloped interface. Furthermore, the mobility mismatch between interfaces I and II amplifies the facet alternation, stabilizing the jagged PB structure.


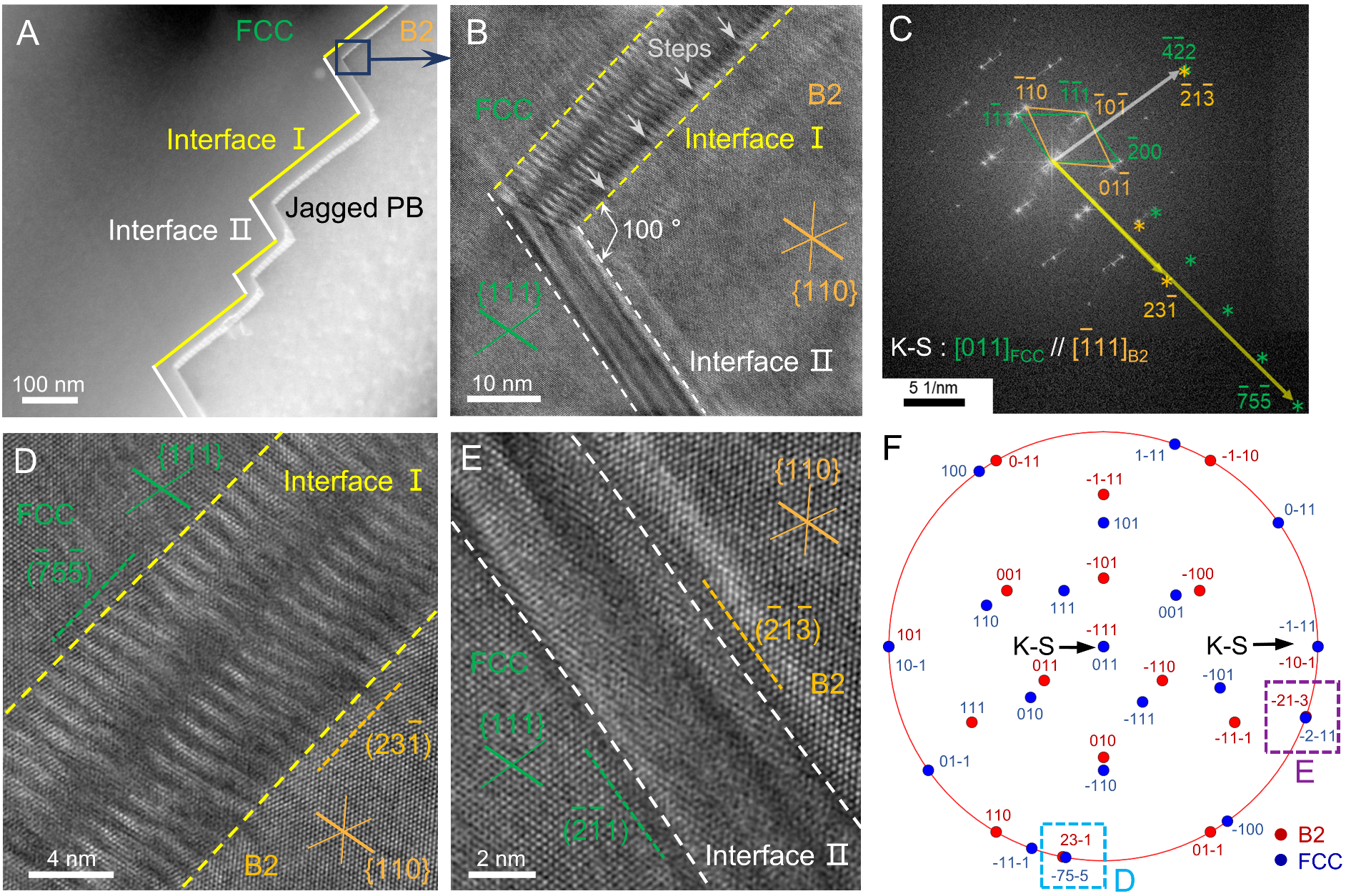


**Figure S17**. **High-resolution crystallographic characterization of the jagged FCC/B2 PB in the 600A alloy.** (**A**) High-angle annular dark-field scanning transmission electron microscopy (HAADF-STEM) image of the jagged PB. (**B**) High-resolution transmission electron microscopy (HR-TEM) image of the jagged PB. (**C**) Corresponding fast Fourier transform (FFT) pattern from the interface, showing two primary orientation relationships (ORs): OR1 as ($\bar{7}5\bar{5}$)_FCC_//($23\bar{1}$)_B2_ and OR2 as ($\bar{2}\bar{1}1$)_FCC_//($\bar{2}1\bar{3}$)_B2_. The orange and green markers indicate the extrapolated reciprocal lattices for the B2 and FCC phases, respectively. (**D**) Atomic-resolution HR-TEM image of Interface Ⅰ, corresponding to the OR1 orientation. (**E**) Atomic-resolution HR-TEM image of Interface Ⅱ, corresponding to the OR2 orientation. (**F**) Composite [$011$]_FCC_ // [$\bar{1}11$]_B2_ stereographic projection based on the experimentally determined Kurdjumov-Sachs (K-S) orientation relationship shown in (C). Interface Ⅰ is mainly characterized by high-density complex interface dislocations. Interface Ⅱ shows a highly coherent feature, well consistent with the result revealed by Xiong et. al. ^[93]^.

**Note 4: The jagged PB weakens the co-deformation between the FCC phase and the B2 phase.** To evaluate the co-deformation capability of the jagged PBs, we assessed the geometric compatibility of active slip systems between the FCC and B2 phases under the Kurdjumov-Sachs (K-S) orientation relationship. This analysis specifically considers dislocation transfer from the {111} <110> slip systems in the FCC phase to the {110}<111> slip systems in the B2 phase. The feasibility of slip transmission can be quantitatively described by the slip-transfer geometric factor ^[96]^, χ, expressed as:

$$\begin{aligned} \chi=\left\langle\left. cos\left( \frac{\pi}{2}\frac{\theta}{\theta_{c}} \right) \right\rangle\cdot\right.\left\langle\left. cos\left( \frac{\pi}{2}\frac{\kappa}{\kappa_{c}} \right) \right\rangle\right. \#(3) \end{aligned}$$

where θ is the angle between the intersection lines of the slip planes with the interface, and κ is the angle between the Burgers vectors of the incoming and outgoing dislocations. The critical angles for transmission are defined as θ_c_ = 15° and κ_c_ = 45°. The brackets, ⟨⟩, indicate that if either angle θ or κ exceeds its respective critical value, the corresponding term becomes zero, rendering slip transfer prohibitive. Consequently, a higher value of χ signifies lower interfacial resistance to dislocation motion and a more energetically favorable slip transfer process.

The slip compatibility of the newly formed Interface Ⅰ in coordinating plastic deformation between the B2 and FCC phases has been quantitatively evaluated by calculating its slip-transfer geometric factor χ ^[96]^, as presented in Table S7. Interface Ⅱ exhibits higher χ and a larger count of high-χ FCC-B2 slip system pairs. Approximately two-thirds of the twelve FCC{111}⟨110⟩ systems have transmissible counterparts across this type of PB ^[93]^. However, Interface Ⅰ shows lower χ and roughly one-third transmissible pairs because the local misfit, high-density steps, and faceting disrupt crystallographic continuity. Bright-field scanning transmission electron microscopy (BF-STEM) confirms these predictions. Figure S14 shows planar slip bands and dislocations in the FCC(L1_2_) matrix are strongly inhibited at the jagged PB, forming severe dislocation pile-ups and leading to significant stress concentration at the jagged PB.

**Table S7.** Computation results of the *θ*, *κ*, and *χ* of the slip system pairs between the B2 and FCC phases, considering the {111}<110> slip systems in the FCC phase and {110}<111> slip systems in the B2 phase. For each slip system in the FCC phase, the paired slip systems in the B2 phase with the non-zero *χ* are marked with red color. These calculations are based on the revealed K-S relationship in Figure S17.

| Interface Ⅰ: ($\bar{7}5\bar{5}$)_FCC_//($23\bar{1}$)_B2_ | | | | | |
| --- | --- | --- | --- | --- | --- |
| Slip systems in the FCC phase and intersection lines of the slip planes with the interface | Slip systems in the B2 phase | Intersection lines of the slip planes in the B2 phase with the interface | $\theta$ (º) | $\kappa$ (º) | $\chi$ |
| $\left( 111 \right)[\bar{1}01]$*_FCC_*  with the intersection line $[\bar{5}\bar{1}6]$*_FCC_* | $\left( 101 \right)\left[ \bar{1}11 \right]$*_B2_* | $\left[ \bar{1}11 \right]$*_B2_* | 63 | 60 | 0 |
|  | $\left( 101 \right)\left[ 11\bar{1} \right]$*_B2_* | $\left[ \bar{1}11 \right]$*_B2_* | 63 | 54.91 | 0 |
|  | $\left( 0\bar{1}1 \right)\left[ \bar{1}11 \right]$*_B2_* | $\left[ 1\bar{1}\bar{1} \right]$*_B2_* | 63 | 60 | 0 |
|  | $\left( 0\bar{1}1 \right)\left[ 111 \right]$*_B2_* | $\left[ 1\bar{1}\bar{1} \right]$*_B2_* | 63 | 54.91 | 0 |
|  | $\left( 110 \right)\left[ \bar{1}11 \right]$*_B2_* | $\left[ \bar{1}11 \right]$*_B2_* | 63 | 60 | 0 |
|  | $\left( 110 \right)\left[ 1\bar{1}1 \right]$*_B2_* | $\left[ \bar{1}11 \right]$*_B2_* | 63 | 49.47 | 0 |
|  | $\left( \bar{1}10 \right)\left[ 111 \right]$*_B2_* | $\left[ \bar{1}\bar{1}1 \right]$*_B2_* | 20 | 54.91 | 0 |
|  | $\left( \bar{1}10 \right)\left[ 11\bar{1} \right]$*_B2_* | $\left[ \bar{1}\bar{1}1 \right]$*_B2_* | 20 | 54.91 | 0 |
|  | $\left( 011 \right)\left[ 1\bar{1}1 \right]$*_B2_* | $\left[ \bar{2}\bar{1}1 \right]$*_B2_* | 70 | 49.47 | 0 |
|  | $\left( 011 \right)\left[ 11\bar{1} \right]$*_B2_* | $\left[ \bar{2}\bar{1}1 \right]$*_B2_* | 70 | 54.91 | 0 |
|  | $\left( \bar{1}01 \right)\left[ 1\bar{1}1 \right]$*_B2_* | $\left[ 151 \right]$*_B2_* | 82 | 49.47 | 0 |
|  | $\left( \bar{1}01 \right)\left[ 111 \right]$*_B_* | $\left[ 151 \right]$*_B2_* | 82 | 54.91 | 0 |
| $\left( 111 \right)[0\bar{1}1]$*_FCC_*  with the intersection line $[\bar{5}\bar{1}6]$*_FCC_* | $\left( 101 \right)\left[ \bar{1}11 \right]$*_B2_* | $\left[ \bar{1}11 \right]$*_B2_* | 63 | 60 | 0 |
|  | $\left( 101 \right)\left[ 11\bar{1} \right]$*_B2_* | $\left[ \bar{1}11 \right]$*_B2_* | 63 | 76.02 | 0 |
|  | $\left( 0\bar{1}1 \right)\left[ \bar{1}11 \right]$*_B2_* | $\left[ 1\bar{1}\bar{1} \right]$*_B2_* | 63 | 60 | 0 |
|  | $\left( 0\bar{1}1 \right)\left[ 111 \right]$*_B2_* | $\left[ 1\bar{1}\bar{1} \right]$*_B2_* | 63 | 76.02 | 0 |
|  | $\left( 110 \right)\left[ \bar{1}11 \right]$*_B2_* | $\left[ \bar{1}11 \right]$*_B2_* | 63 | 60 | 0 |
|  | $\left( 110 \right)\left[ 1\bar{1}1 \right]$*_B2_* | $\left[ \bar{1}11 \right]$*_B2_* | 63 | 10.53 | 0 |
|  | $\left( \bar{1}10 \right)\left[ 111 \right]$*_B2_* | $\left[ \bar{1}\bar{1}1 \right]$*_B2_* | 20 | 76.02 | 0 |
|  | $\left( \bar{1}10 \right)\left[ 11\bar{1} \right]$*_B2_* | $\left[ \bar{1}\bar{1}1 \right]$*_B2_* | 20 | 76.02 | 0 |
|  | $\left( 011 \right)\left[ 1\bar{1}1 \right]$*_B2_* | $\left[ \bar{2}\bar{1}1 \right]$*_B2_* | 70 | 10.53 | 0 |
|  | $\left( 011 \right)\left[ 11\bar{1} \right]$*_B2_* | $\left[ \bar{2}\bar{1}1 \right]$*_B2_* | 70 | 76.02 | 0 |
|  | $\left( \bar{1}01 \right)\left[ 1\bar{1}1 \right]$*_B2_* | $\left[ 151 \right]$*_B2_* | 82 | 10.53 | 0 |
|  | $\left( \bar{1}01 \right)\left[ 111 \right]$*_B_* | $\left[ 151 \right]$*_B2_* | 82 | 76.02 | 0 |
| $\left( 111 \right)[\bar{1}10]$*_FCC_*  with the intersection line $[\bar{5}\bar{1}6]$*_FCC_* | $\left( 101 \right)\left[ \bar{1}11 \right]$*_B2_* | $\left[ \bar{1}11 \right]$*_B2_* | 63 | 0 | 0 |
|  | $\left( 101 \right)\left[ 11\bar{1} \right]$*_B2_* | $\left[ \bar{1}11 \right]$*_B2_* | 63 | 70.53 | 0 |
|  | $\left( 0\bar{1}1 \right)\left[ \bar{1}11 \right]$*_B2_* | $\left[ 1\bar{1}\bar{1} \right]$*_B2_* | 63 | 0 | 0 |
|  | $\left( 0\bar{1}1 \right)\left[ 111 \right]$*_B2_* | $\left[ 1\bar{1}\bar{1} \right]$*_B2_* | 63 | 70.53 | 0 |
|  | $\left( 110 \right)\left[ \bar{1}11 \right]$*_B2_* | $\left[ \bar{1}11 \right]$*_B2_* | 63 | 0 | 0 |
|  | $\left( 110 \right)\left[ 1\bar{1}1 \right]$*_B2_* | $\left[ \bar{1}11 \right]$*_B2_* | 63 | 70.53 | 0 |
|  | $\left( \bar{1}10 \right)\left[ 111 \right]$*_B2_* | $\left[ \bar{1}\bar{1}1 \right]$*_B2_* | 20 | 70.53 | 0 |
|  | $\left( \bar{1}10 \right)\left[ 11\bar{1} \right]$*_B2_* | $\left[ \bar{1}\bar{1}1 \right]$*_B2_* | 20 | 70.53 | 0 |
|  | $\left( 011 \right)\left[ 1\bar{1}1 \right]$*_B2_* | $\left[ \bar{2}\bar{1}1 \right]$*_B2_* | 70 | 70.53 | 0 |
|  | $\left( 011 \right)\left[ 11\bar{1} \right]$*_B2_* | $\left[ \bar{2}\bar{1}1 \right]$*_B2_* | 70 | 70.53 | 0 |
|  | $\left( \bar{1}01 \right)\left[ 1\bar{1}1 \right]$*_B2_* | $\left[ 151 \right]$*_B2_* | 82 | 70.53 | 0 |
|  | $\left( \bar{1}01 \right)\left[ 111 \right]$*_B_* | $\left[ 151 \right]$*_B2_* | 82 | 70.53 | 0 |
| $\left( \bar{1}11 \right)[110]$*_FCC_*  with the intersection line  $[\bar{5}\bar{6}1]$*_FCC_* | $\left( 101 \right)\left[ \bar{1}11 \right]$*_B2_* | $\left[ \bar{1}11 \right]$*_B2_* | 63 | 90 | 0 |
|  | $\left( 101 \right)\left[ 11\bar{1} \right]$*_B2_* | $\left[ \bar{1}11 \right]$*_B2_* | 63 | 20.14 | 0 |
|  | $\left( 0\bar{1}1 \right)\left[ \bar{1}11 \right]$*_B2_* | $\left[ 1\bar{1}\bar{1} \right]$*_B2_* | 63 | 90 | 0 |
|  | $\left( 0\bar{1}1 \right)\left[ 111 \right]$*_B2_* | $\left[ 1\bar{1}\bar{1} \right]$*_B2_* | 63 | 66.77 | 0 |
|  | $\left( 110 \right)\left[ \bar{1}11 \right]$*_B2_* | $\left[ \bar{1}11 \right]$*_B2_* | 63 | 90 | 0 |
|  | $\left( 110 \right)\left[ 1\bar{1}1 \right]$*_B2_* | $\left[ \bar{1}11 \right]$*_B2_* | 63 | 57.02 | 0 |
|  | $\left( \bar{1}10 \right)\left[ 111 \right]$*_B2_* | $\left[ \bar{1}\bar{1}1 \right]$*_B2_* | 63 | 66.77 | 0 |
|  | $\left( \bar{1}10 \right)\left[ 11\bar{1} \right]$*_B2_* | $\left[ \bar{1}\bar{1}1 \right]$*_B2_* | 63 | 20.14 | 0 |
|  | $\left( 011 \right)\left[ 1\bar{1}1 \right]$*_B2_* | $\left[ \bar{2}\bar{1}1 \right]$*_B2_* | 85 | 57.02 | 0 |
|  | $\left( 011 \right)\left[ 11\bar{1} \right]$*_B2_* | $\left[ \bar{2}\bar{1}1 \right]$*_B2_* | 85 | 20.14 | 0 |
|  | $\left( \bar{1}01 \right)\left[ 1\bar{1}1 \right]$*_B2_* | $\left[ 151 \right]$*_B2_* | 68 | 57.02 | 0 |
|  | $\left( \bar{1}01 \right)\left[ 111 \right]$*_B_* | $\left[ 151 \right]$*_B2_* | 68 | 66.77 | 0 |
| $\left( \bar{1}11 \right)[101]$*_FCC_*  with the intersection line  $[\bar{5}\bar{6}1]$*_FCC_* | $\left( 101 \right)\left[ \bar{1}11 \right]$*_B2_* | $\left[ \bar{1}11 \right]$*_B2_* | 63 | 60 | 0 |
|  | $\left( 101 \right)\left[ 11\bar{1} \right]$*_B2_* | $\left[ \bar{1}11 \right]$*_B2_* | 63 | 45.79 | 0 |
|  | $\left( 0\bar{1}1 \right)\left[ \bar{1}11 \right]$*_B2_* | $\left[ 1\bar{1}\bar{1} \right]$*_B2_* | 63 | 60 | 0 |
|  | $\left( 0\bar{1}1 \right)\left[ 111 \right]$*_B2_* | $\left[ 1\bar{1}\bar{1} \right]$*_B2_* | 63 | 50.5 | 0 |
|  | $\left( 110 \right)\left[ \bar{1}11 \right]$*_B2_* | $\left[ \bar{1}11 \right]$*_B2_* | 63 | 60 | 0 |
|  | $\left( 110 \right)\left[ 1\bar{1}1 \right]$*_B2_* | $\left[ \bar{1}11 \right]$*_B2_* | 63 | 63.97 | 0 |
|  | $\left( \bar{1}10 \right)\left[ 111 \right]$*_B2_* | $\left[ \bar{1}\bar{1}1 \right]$*_B2_* | 63 | 50.5 | 0 |
|  | $\left( \bar{1}10 \right)\left[ 11\bar{1} \right]$*_B2_* | $\left[ \bar{1}\bar{1}1 \right]$*_B2_* | 63 | 45.79 | 0 |
|  | $\left( 011 \right)\left[ 1\bar{1}1 \right]$*_B2_* | $\left[ \bar{2}\bar{1}1 \right]$*_B2_* | 85 | 63.97 | 0 |
|  | $\left( 011 \right)\left[ 11\bar{1} \right]$*_B2_* | $\left[ \bar{2}\bar{1}1 \right]$*_B2_* | 85 | 45.79 | 0 |
|  | $\left( \bar{1}01 \right)\left[ 1\bar{1}1 \right]$*_B2_* | $\left[ 151 \right]$*_B2_* | 68 | 63.97 | 0 |
|  | $\left( \bar{1}01 \right)\left[ 111 \right]$*_B_* | $\left[ 151 \right]$*_B2_* | 68 | 50.5 | 0 |
| $\left( \bar{1}11 \right)[0\bar{1}1]$*_FCC_*  with the intersection line  $[\bar{5}\bar{6}1]$*_FCC_* | $\left( 101 \right)\left[ \bar{1}11 \right]$*_B2_* | $\left[ \bar{1}11 \right]$*_B2_* | 63 | 60 | 0 |
|  | $\left( 101 \right)\left[ 11\bar{1} \right]$*_B2_* | $\left[ \bar{1}11 \right]$*_B2_* | 63 | 76.02 | 0 |
|  | $\left( 0\bar{1}1 \right)\left[ \bar{1}11 \right]$*_B2_* | $\left[ 1\bar{1}\bar{1} \right]$*_B2_* | 63 | 60 | 0 |
|  | $\left( 0\bar{1}1 \right)\left[ 111 \right]$*_B2_* | $\left[ 1\bar{1}\bar{1} \right]$*_B2_* | 63 | 76.02 | 0 |
|  | $\left( 110 \right)\left[ \bar{1}11 \right]$*_B2_* | $\left[ \bar{1}11 \right]$*_B2_* | 63 | 60 | 0 |
|  | $\left( 110 \right)\left[ 1\bar{1}1 \right]$*_B2_* | $\left[ \bar{1}11 \right]$*_B2_* | 63 | 10.53 | 0 |
|  | $\left( \bar{1}10 \right)\left[ 111 \right]$*_B2_* | $\left[ \bar{1}\bar{1}1 \right]$*_B2_* | 63 | 76.02 | 0 |
|  | $\left( \bar{1}10 \right)\left[ 11\bar{1} \right]$*_B2_* | $\left[ \bar{1}\bar{1}1 \right]$*_B2_* | 63 | 76.02 | 0 |
|  | $\left( 011 \right)\left[ 1\bar{1}1 \right]$*_B2_* | $\left[ \bar{2}\bar{1}1 \right]$*_B2_* | 85 | 10.53 | 0 |
|  | $\left( 011 \right)\left[ 11\bar{1} \right]$*_B2_* | $\left[ \bar{2}\bar{1}1 \right]$*_B2_* | 85 | 76.02 | 0 |
|  | $\left( \bar{1}01 \right)\left[ 1\bar{1}1 \right]$*_B2_* | $\left[ 151 \right]$*_B2_* | 68 | 10.53 | 0 |
|  | $\left( \bar{1}01 \right)\left[ 111 \right]$*_B_* | $\left[ 151 \right]$*_B2_* | 68 | 76.02 | 0 |
| $\left( 1\bar{1}1 \right)[110]$*_FCC_*  with the intersection line  $[011]$*_FCC_* | $\left( 101 \right)\left[ \bar{1}11 \right]$*_B2_* | $\left[ \bar{1}11 \right]$*_B2_* | 0 | 90 | 0 |
|  | $\left( \mathbf{101} \right)\left[ \boldsymbol{11}\bar{\boldsymbol{1}} \right]$***_B2_*** | $\left[ \bar{\boldsymbol{1}}\mathbf{11} \right]$***_B2_*** | **0** | **20.14** | **1** |
|  | $\left( 0\bar{1}1 \right)\left[ \bar{1}11 \right]$*_B2_* | $\left[ 1\bar{1}\bar{1} \right]$*_B2_* | 0 | 90 | 0 |
|  | $\left( 0\bar{1}1 \right)\left[ 111 \right]$*_B2_* | $\left[ 1\bar{1}\bar{1} \right]$*_B2_* | 0 | 66.77 | 0 |
|  | $\left( 110 \right)\left[ \bar{1}11 \right]$*_B2_* | $\left[ \bar{1}11 \right]$*_B2_* | 0 | 90 | 0 |
|  | $\left( 110 \right)\left[ 1\bar{1}1 \right]$*_B2_* | $\left[ \bar{1}11 \right]$*_B2_* | 0 | 57.02 | 0 |
|  | $\left( \bar{1}10 \right)\left[ 111 \right]$*_B2_* | $\left[ \bar{1}\bar{1}1 \right]$*_B2_* | 70.53 | 66.77 | 0 |
|  | $\left( \bar{1}10 \right)\left[ 11\bar{1} \right]$*_B2_* | $\left[ \bar{1}\bar{1}1 \right]$*_B2_* | 70.53 | 20.14 | 0 |
|  | $\left( 011 \right)\left[ 1\bar{1}1 \right]$*_B2_* | $\left[ \bar{2}\bar{1}1 \right]$*_B2_* | 61.87 | 57.02 | 0 |
|  | $\left( 011 \right)\left[ 11\bar{1} \right]$*_B2_* | $\left[ \bar{2}\bar{1}1 \right]$*_B2_* | 61.87 | 20.14 | 0 |
|  | $\left( \bar{1}01 \right)\left[ 1\bar{1}1 \right]$*_B2_* | $\left[ 151 \right]$*_B2_* | 56.25 | 57.02 | 0 |
|  | $\left( \bar{1}01 \right)\left[ 111 \right]$*_B_* | $\left[ 151 \right]$*_B2_* | 56.25 | 66.77 | 0 |
| $\left( 1\bar{1}1 \right)[011]$*_FCC_*  with the intersection line  $[011]$*_FCC_* | $\left( 101 \right)\left[ \bar{1}11 \right]$*_B2_* | $\left[ \bar{1}11 \right]$*_B2_* | 0 | 60 | 0 |
|  | $\left( 101 \right)\left[ 11\bar{1} \right]$*_B2_* | $\left[ \bar{1}11 \right]$*_B2_* | 0 | 68.66 | 0 |
|  | $\left( 0\bar{1}1 \right)\left[ \bar{1}11 \right]$*_B2_* | $\left[ 1\bar{1}\bar{1} \right]$*_B2_* | 0 | 60 | 0 |
|  | $\left( \mathbf{0}\bar{\boldsymbol{1}}\mathbf{1} \right)\left[ \mathbf{1}\boldsymbol{11} \right]$***_B2_*** | $\left[ \boldsymbol{1}\bar{\boldsymbol{1}}\bar{\boldsymbol{1}} \right]$***_B2_*** | **0** | **14.21** | **1** |
|  | $\left( 110 \right)\left[ \bar{1}11 \right]$*_B2_* | $\left[ \bar{1}11 \right]$*_B2_* | 0 | 60 | 0 |
|  | $\left( 110 \right)\left[ 1\bar{1}1 \right]$*_B2_* | $\left[ \bar{1}11 \right]$*_B2_* | 0 | 83.94 | 0 |
|  | $\left( \bar{1}10 \right)\left[ 111 \right]$*_B2_* | $\left[ \bar{1}\bar{1}1 \right]$*_B2_* | 70.53 | 14.21 | 0 |
|  | $\left( \bar{1}10 \right)\left[ 11\bar{1} \right]$*_B2_* | $\left[ \bar{1}\bar{1}1 \right]$*_B2_* | 70.53 | 68.66 | 0 |
|  | $\left( 011 \right)\left[ 1\bar{1}1 \right]$*_B2_* | $\left[ \bar{2}\bar{1}1 \right]$*_B2_* | 61.87 | 83.94 | 0 |
|  | $\left( 011 \right)\left[ 11\bar{1} \right]$*_B2_* | $\left[ \bar{2}\bar{1}1 \right]$*_B2_* | 61.87 | 68.66 | 0 |
|  | $\left( \bar{1}01 \right)\left[ 1\bar{1}1 \right]$*_B2_* | $\left[ 151 \right]$*_B2_* | 56.25 | 83.94 | 0 |
|  | $\left( \bar{1}01 \right)\left[ 111 \right]$*_B_* | $\left[ 151 \right]$*_B2_* | 56.25 | 14.21 | 0 |
| $\left( 1\bar{1}1 \right)[\bar{1}01]$*_FCC_*  with the intersection line  $[011]$*_FCC_* | $\left( 101 \right)\left[ \bar{1}11 \right]$*_B2_* | $\left[ \bar{1}11 \right]$*_B2_* | 0 | 60 | 0 |
|  | $\left( 101 \right)\left[ 11\bar{1} \right]$*_B2_* | $\left[ \bar{1}11 \right]$*_B2_* | 0 | 54.91 | 0 |
|  | $\left( 0\bar{1}1 \right)\left[ \bar{1}11 \right]$*_B2_* | $\left[ 1\bar{1}\bar{1} \right]$*_B2_* | 0 | 60 | 0 |
|  | $\left( 0\bar{1}1 \right)\left[ 111 \right]$*_B2_* | $\left[ 1\bar{1}\bar{1} \right]$*_B2_* | 0 | 54.91 | 0 |
|  | $\left( 110 \right)\left[ \bar{1}11 \right]$*_B2_* | $\left[ \bar{1}11 \right]$*_B2_* | 0 | 60 | 0 |
|  | $\left( 110 \right)\left[ 1\bar{1}1 \right]$*_B2_* | $\left[ \bar{1}11 \right]$*_B2_* | 0 | 49.47 | 0 |
|  | $\left( \bar{1}10 \right)\left[ 111 \right]$*_B2_* | $\left[ \bar{1}\bar{1}1 \right]$*_B2_* | 70.53 | 54.91 | 0 |
|  | $\left( \bar{1}10 \right)\left[ 11\bar{1} \right]$*_B2_* | $\left[ \bar{1}\bar{1}1 \right]$*_B2_* | 70.53 | 54.91 | 0 |
|  | $\left( 011 \right)\left[ 1\bar{1}1 \right]$*_B2_* | $\left[ \bar{2}\bar{1}1 \right]$*_B2_* | 61.87 | 49.47 | 0 |
|  | $\left( 011 \right)\left[ 11\bar{1} \right]$*_B2_* | $\left[ \bar{2}\bar{1}1 \right]$*_B2_* | 61.87 | 54.91 | 0 |
|  | $\left( \bar{1}01 \right)\left[ 1\bar{1}1 \right]$*_B2_* | $\left[ 151 \right]$*_B2_* | 56.25 | 49.47 | 0 |
|  | $\left( \bar{1}01 \right)\left[ 111 \right]$*_B_* | $\left[ 151 \right]$*_B2_* | 56.25 | 54.91 | 0 |
| $\left( 11\bar{1} \right)[011]$*_FCC_*  with the intersection line  $[011]$*_FCC_* | $\left( 101 \right)\left[ \bar{1}11 \right]$*_B2_* | $\left[ \bar{1}11 \right]$*_B2_* | 0 | 60 | 0 |
|  | $\left( 101 \right)\left[ 11\bar{1} \right]$*_B2_* | $\left[ \bar{1}11 \right]$*_B2_* | 0 | 68.66 | 0 |
|  | $\left( 0\bar{1}1 \right)\left[ \bar{1}11 \right]$*_B2_* | $\left[ 1\bar{1}\bar{1} \right]$*_B2_* | 0 | 60 | 0 |
|  | $\left( \mathbf{0}\bar{\boldsymbol{1}}\mathbf{1} \right)\left[ \mathbf{1}\boldsymbol{11} \right]$***_B2_*** | $\left[ \boldsymbol{1}\bar{\boldsymbol{1}}\bar{\boldsymbol{1}} \right]$***_B2_*** | **0** | **14.21** | **1** |
|  | $\left( 110 \right)\left[ \bar{1}11 \right]$*_B2_* | $\left[ \bar{1}11 \right]$*_B2_* | 0 | 60 | 0 |
|  | $\left( 110 \right)\left[ 1\bar{1}1 \right]$*_B2_* | $\left[ \bar{1}11 \right]$*_B2_* | 0 | 83.94 | 0 |
|  | $\left( \bar{1}10 \right)\left[ 111 \right]$*_B2_* | $\left[ \bar{1}\bar{1}1 \right]$*_B2_* | 70.53 | 14.21 | 0 |
|  | $\left( \bar{1}10 \right)\left[ 11\bar{1} \right]$*_B2_* | $\left[ \bar{1}\bar{1}1 \right]$*_B2_* | 70.53 | 68.66 | 0 |
|  | $\left( 011 \right)\left[ 1\bar{1}1 \right]$*_B2_* | $\left[ \bar{2}\bar{1}1 \right]$*_B2_* | 61.87 | 83.94 | 0 |
|  | $\left( 011 \right)\left[ 11\bar{1} \right]$*_B2_* | $\left[ \bar{2}\bar{1}1 \right]$*_B2_* | 61.87 | 68.66 | 0 |
|  | $\left( \bar{1}01 \right)\left[ 1\bar{1}1 \right]$*_B2_* | $\left[ 151 \right]$*_B2_* | 56.25 | 83.94 | 0 |
|  | $\left( \bar{1}01 \right)\left[ 111 \right]$*_B_* | $\left[ 151 \right]$*_B2_* | 56.25 | 14.21 | 0 |
| $\left( 11\bar{1} \right)[101]$*_FCC_*  with the intersection line  $[011]$*_FCC_* | $\left( 101 \right)\left[ \bar{1}11 \right]$*_B2_* | $\left[ \bar{1}11 \right]$*_B2_* | 0 | 60 | 0 |
|  | $\left( 101 \right)\left[ 11\bar{1} \right]$*_B2_* | $\left[ \bar{1}11 \right]$*_B2_* | 0 | 45.79 | 0 |
|  | $\left( 0\bar{1}1 \right)\left[ \bar{1}11 \right]$*_B2_* | $\left[ 1\bar{1}\bar{1} \right]$*_B2_* | 0 | 60 | 0 |
|  | $\left( 0\bar{1}1 \right)\left[ 111 \right]$*_B2_* | $\left[ 1\bar{1}\bar{1} \right]$*_B2_* | 0 | 50.5 | 0 |
|  | $\left( 110 \right)\left[ \bar{1}11 \right]$*_B2_* | $\left[ \bar{1}11 \right]$*_B2_* | 0 | 60 | 0 |
|  | $\left( 110 \right)\left[ 1\bar{1}1 \right]$*_B2_* | $\left[ \bar{1}11 \right]$*_B2_* | 0 | 63.97 | 0 |
|  | $\left( \bar{1}10 \right)\left[ 111 \right]$*_B2_* | $\left[ \bar{1}\bar{1}1 \right]$*_B2_* | 70.53 | 50.5 | 0 |
|  | $\left( \bar{1}10 \right)\left[ 11\bar{1} \right]$*_B2_* | $\left[ \bar{1}\bar{1}1 \right]$*_B2_* | 70.53 | 45.79 | 0 |
|  | $\left( 011 \right)\left[ 1\bar{1}1 \right]$*_B2_* | $\left[ \bar{2}\bar{1}1 \right]$*_B2_* | 61.87 | 63.97 | 0 |
|  | $\left( 011 \right)\left[ 11\bar{1} \right]$*_B2_* | $\left[ \bar{2}\bar{1}1 \right]$*_B2_* | 61.87 | 45.79 | 0 |
|  | $\left( \bar{1}01 \right)\left[ 1\bar{1}1 \right]$*_B2_* | $\left[ 151 \right]$*_B2_* | 56.25 | 63.97 | 0 |
|  | $\left( \bar{1}01 \right)\left[ 111 \right]$*_B_* | $\left[ 151 \right]$*_B2_* | 56.25 | 50.5 | 0 |
| $\left( 11\bar{1} \right)[\bar{1}10]$*_FCC_*  with the intersection line  $[011]$*_FCC_* | $\left( \mathbf{101} \right)\left[ \bar{\boldsymbol{1}}\mathbf{11} \right]$***_B2_*** | $\left[ \bar{\boldsymbol{1}}\mathbf{11} \right]$***_B2_*** | **0** | **0** | **1** |
|  | $\left( 101 \right)\left[ 11\bar{1} \right]$*_B2_* | $\left[ \bar{1}11 \right]$*_B2_* | 0 | 70.53 | 0 |
|  | $\left( \mathbf{0}\bar{\boldsymbol{1}}\mathbf{1} \right)\left[ \bar{\boldsymbol{1}}\boldsymbol{11} \right]$***_B2_*** | $\left[ \boldsymbol{1}\bar{\boldsymbol{1}}\bar{\boldsymbol{1}} \right]$***_B2_*** | **0** | **0** | **1** |
|  | $\left( 0\bar{1}1 \right)\left[ 111 \right]$*_B2_* | $\left[ 1\bar{1}\bar{1} \right]$*_B2_* | 0 | 70.53 | 0 |
|  | $\left( \mathbf{110} \right)\left[ \bar{\boldsymbol{1}}\boldsymbol{11} \right]$***_B2_*** | $\left[ \bar{\boldsymbol{1}}\mathbf{11} \right]$***_B2_*** | **0** | **0** | **1** |
|  | $\left( 110 \right)\left[ 1\bar{1}1 \right]$*_B2_* | $\left[ \bar{1}11 \right]$*_B2_* | 0 | 70.53 | 0 |
|  | $\left( \bar{1}10 \right)\left[ 111 \right]$*_B2_* | $\left[ \bar{1}\bar{1}1 \right]$*_B2_* | 70.53 | 70.53 | 0 |
|  | $\left( \bar{1}10 \right)\left[ 11\bar{1} \right]$*_B2_* | $\left[ \bar{1}\bar{1}1 \right]$*_B2_* | 70.53 | 70.53 | 0 |
|  | $\left( 011 \right)\left[ 1\bar{1}1 \right]$*_B2_* | $\left[ \bar{2}\bar{1}1 \right]$*_B2_* | 61.87 | 70.53 | 0 |
|  | $\left( 011 \right)\left[ 11\bar{1} \right]$*_B2_* | $\left[ \bar{2}\bar{1}1 \right]$*_B2_* | 61.87 | 70.53 | 0 |
|  | $\left( \bar{1}01 \right)\left[ 1\bar{1}1 \right]$*_B2_* | $\left[ 151 \right]$*_B2_* | 56.25 | 70.53 | 0 |
|  | $\left( \bar{1}01 \right)\left[ 111 \right]$*_B_* | $\left[ 151 \right]$*_B2_* | 56.25 | 70.53 | 0 |

**Note 5: Formation mechanism of the CRP-free zone in the 750A alloy.** The thermodynamic calculations indicate that upon elevating the temperature to 750 °C, the solid solubility of Cr in the B2 phase increases substantially (Figure S18A). This change provides a strong driving force for existing CRPs to dissolve into the B2 matrix to re-establish equilibrium (Figure S18B), thereby reducing the overall CRPs volume fraction (Figure S18C). This increase of Cr in the B2 matrix, in turn, establishes a high chemical potential gradient between the B2 and FCC phases (Figure S18D), which provides the driving force for net Cr diffusion from the B2 phase into the FCC(L1_2_) matrix. Moreover, kinetic calculations (Figure S18E) reveal that the Cr diffusion coefficient at 750 °C is sufficiently high to achieve the Cr diffusion from B2 to FCC(L1_2_) matrix, which lowers the local equilibrium Cr concentration on the B2 side. To maintain chemical equilibrium in the B2 matrix, small CRPs adjacent to the PB—particularly those with high interfacial curvature—dissolve first ^[97-99]^, rapidly forming a CRP-depleted zone along the B2 edge. This dissolution process ceases once the Cr concentration in the B2 matrix reaches the equilibrium solubility limit at this temperature, leaving the larger, interior CRPs stable.

Therefore, we propose the following mechanism: (i) rapid formation of a CRP-free zone on the B2 edge at early stage, driven by the chemical-potential gradient and high Cr diffusivity; (ii) self-limiting growth of this zone as the local B2 composition approaches to its 750 °C solubility; and (iii) subsequent evolution dominated by Ostwald ripening of the larger, interior CRPs rather than further advance of the CRP-free zone. The time-dependent aging experiments at 750 °C (5 h→12 h→72 h) (Figures S18G−I) confirm this picture: the CRP-free zone appears early and remains nearly unchanged in width with longer holds, while interior CRPs coarsen progressively.


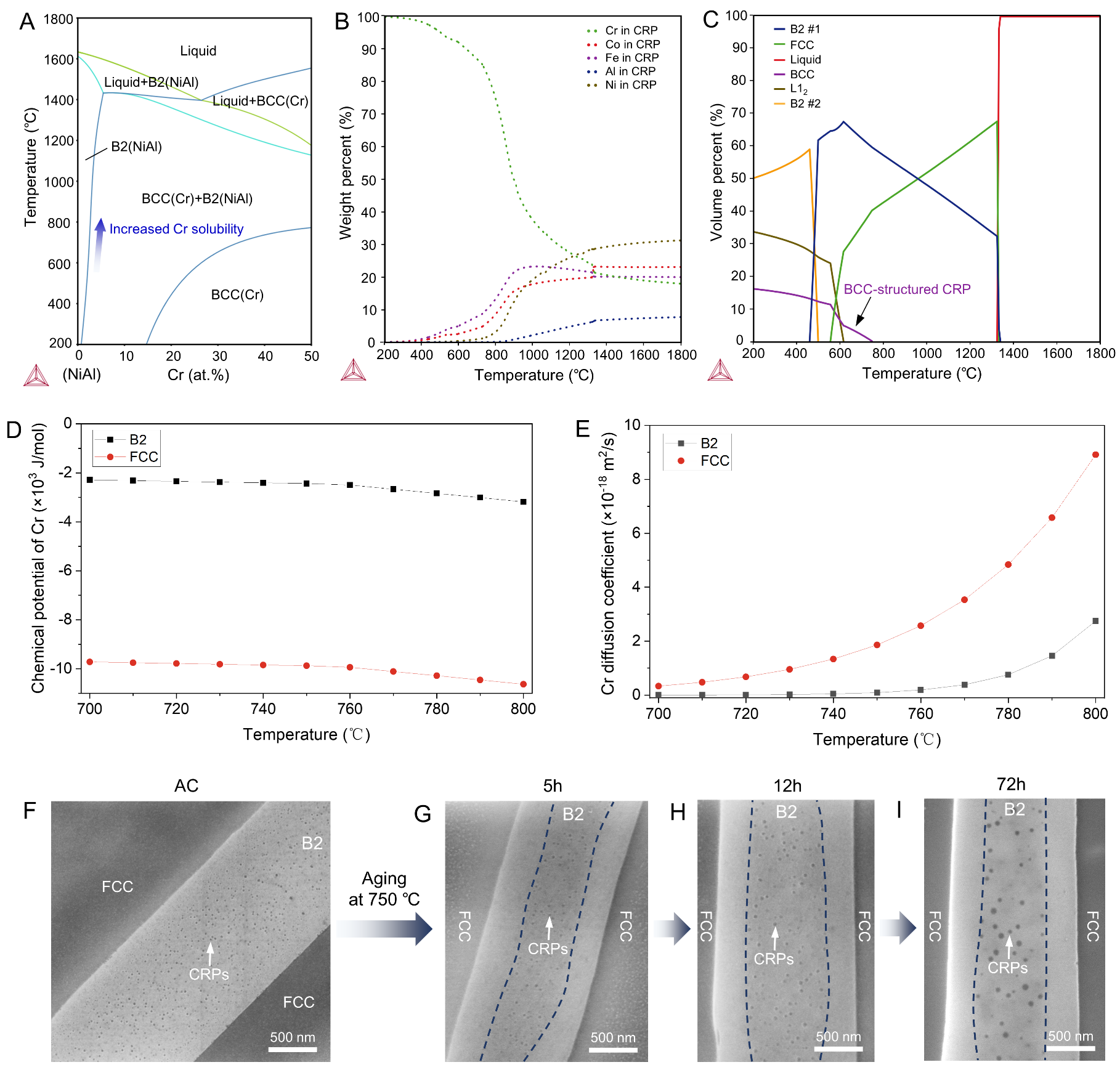


**Figure S18.** **Thermodynamic and experimental investigation of Cr-rich particles (CRPs) evolution in the AlCoCrFeNi_2.1_ EHEA.** (**A**−**E**) Thermodynamic simulations showing: (A) the (NiAl)-Cr pseudo-binary phase diagram, (B) the predicted evolution of Cr content in the BCC-structured CRPs, (C) the calculated vertical-section phase diagram, and (D, E) the chemical potential and diffusion coefficient of Cr in the FCC and B2 phases. (**F**−**I**) Experimental characterization of CRPs, showing: (F) the microstructure of the as-cast (AC) alloy, and (G−I) the subsequent evolution after aging at 750 °C for 5 h, 12 h, and 72 h, respectively. All calculations were performed using Thermo-Calc software with the TCHEA4, TTNi8, and MobNi5 databases.

**Note 6: Influences of aging time on the L1_2_ domains and the alloy's mechanical performance.** The alloy exhibits distinct microstructural and mechanical responses to aging at 600 °C and 750 °C (Figures S19 and S20). When aging at 600 °C, the L1_2_ domains exhibit sluggish growth kinetics. In the early stages, a slight decrease in domain size is observed, likely due to local atomic rearrangement and structural reconfiguration. As aging time increases, the domains grow slowly, and even after prolonged exposure, their size remains smaller than that of the as-cast alloy (Figure S20A), indicating suppressed Ostwald ripening at this temperature. In parallel, the L1_2_ evolution correlates closely with the mechanical properties. The yield strength increases progressively with aging time, while the fracture elongation remains nearly unchanged up to 12 h but drops sharply at 20 h, indicating embrittlement driven by enhanced ordering. This is followed by a plateau in ductility with further aging. The ultimate tensile strength remains relatively stable during early aging, decreases slightly at 20 h, and modestly recovers at 72 h, possibly due to partial coarsening of L1_2_ domains and increased ordering strengthening (Figures S20B–D).

In contrast, at 750 °C, the L1_2_ domains exhibit rapid coarsening due to enhanced diffusion, resulting in significantly larger domains compared to the as-cast state (Figure S19). Interestingly, the fracture elongation slightly exceeds that of the as-cast alloy and remains stable during the early aging period, which we attribute to the formation of Cr-rich particle (CRP)-free zones that enhance plastic accommodation. However, after 72 h, ductility decreases, likely due to B2_Ⅱ_ phase coarsening and its transformation into an acicular morphology, which introduces stress concentration. Both yield and ultimate tensile strength are consistently higher than in the non-aged state, but show little variation with aging time. This suggests that the primary strengthening features are formed early during aging and that further microstructural changes have a relatively minor effect on the strength.


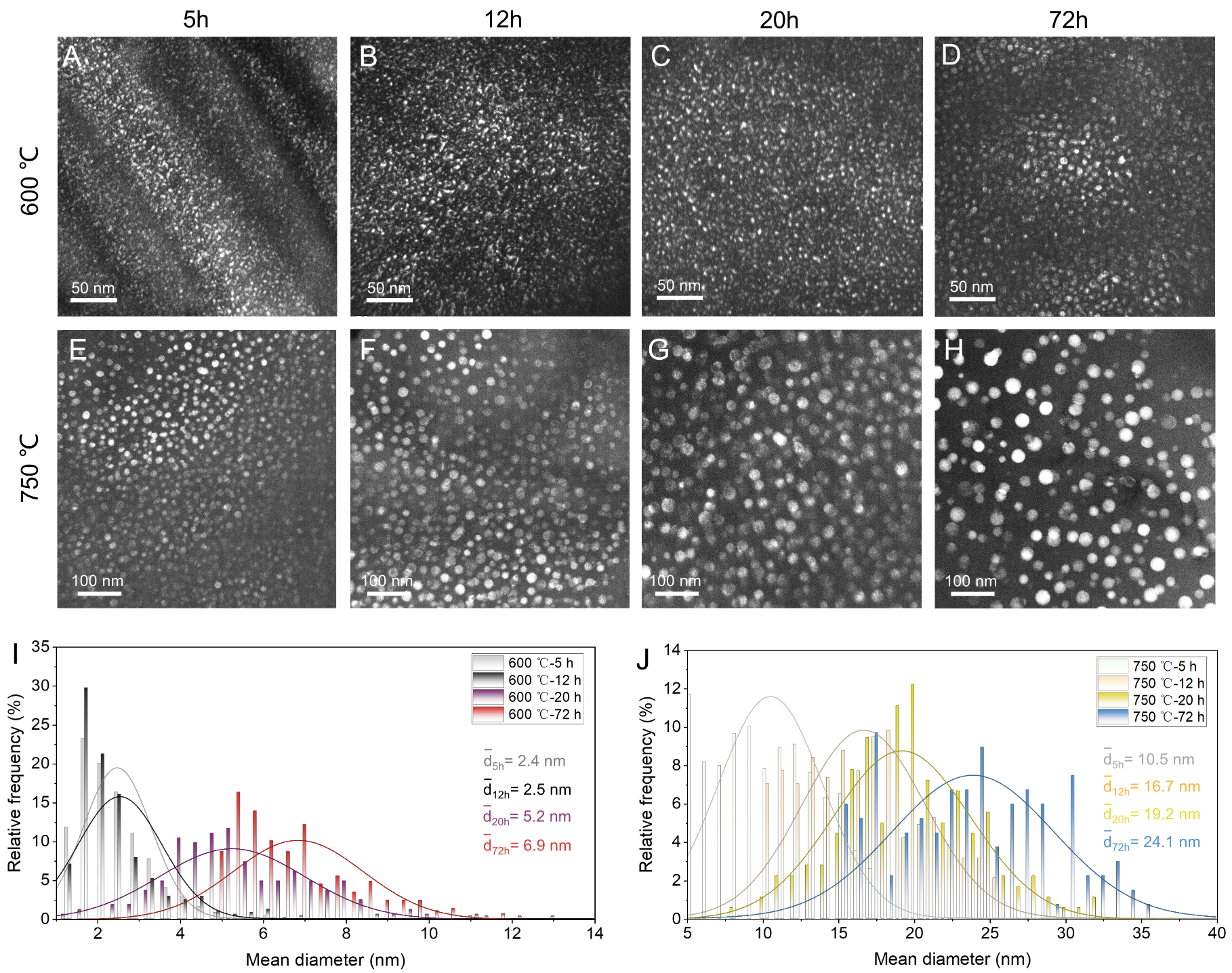


**Figure S19. Evolution of L1_2_-ordered domains during thermal aging.** (**A**–**H**) Dark-field transmission electron microscopy (DF-TEM) images showing the morphology of L1_2_ domains after aging at (A–D) 600 °C and (E–H) 750 °C for 5, 12, 20, and 72 h. (**I**, **J**) Corresponding size distribution histograms of the L1_2_ domains for the samples aged at (I) 600 °C and (J) 750 °C.


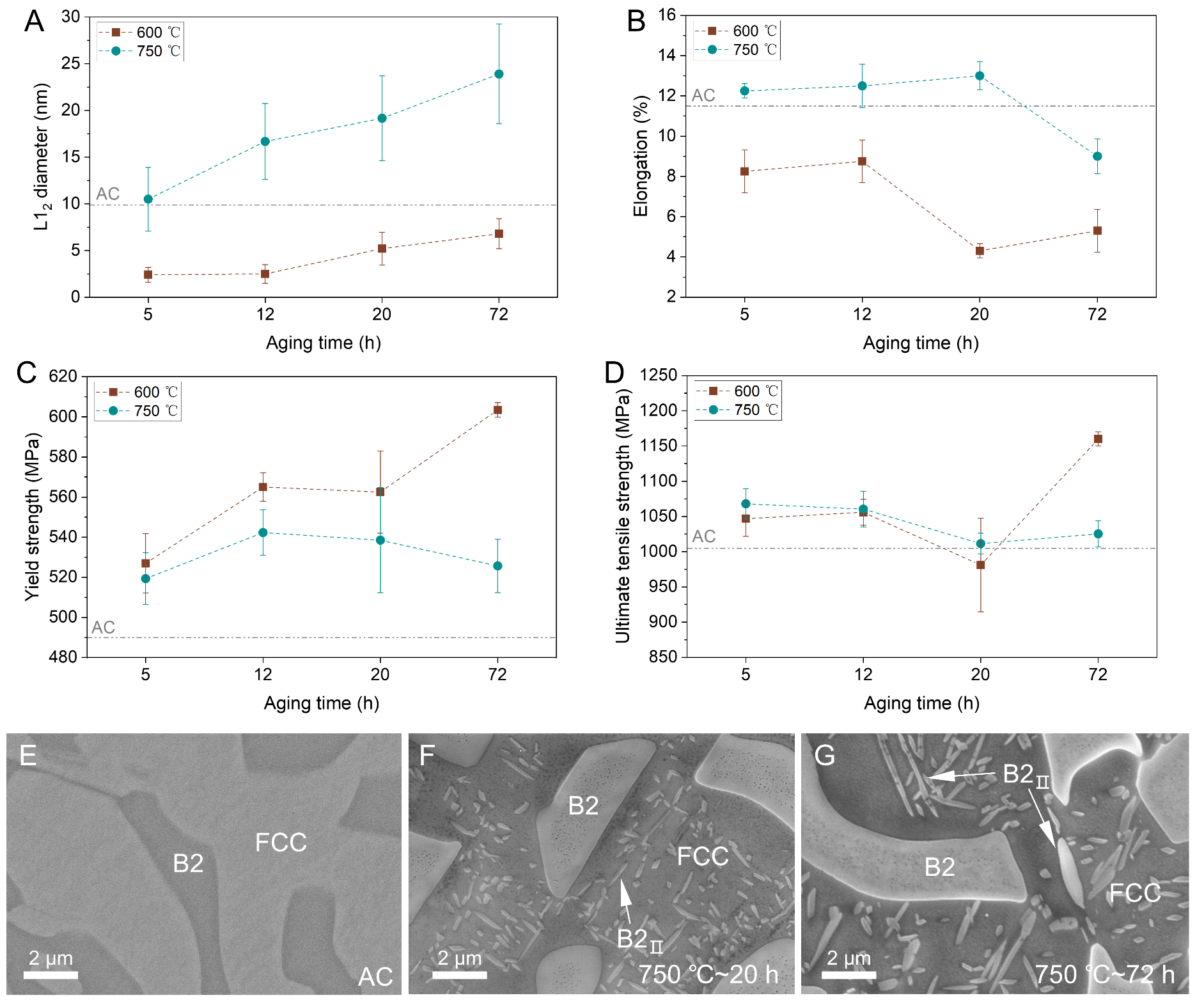


**Figure S20. Effect of aging time on the microstructure and mechanical properties of the alloy.** (**A**) Evolution of the average L1_2_ domain size as a function of aging time. (**B**−**D**) Corresponding changes in mechanical properties: (B) fracture elongation, (C) yield strength, and (D) ultimate tensile strength. (**E**) Microstructure of the as-cast (AC) alloy, compared with the microstructures after aging for (**F**) 20 h and (**G**) 72 h.

**Supplementary references：**

[1] Y. Guo, H. Su, H. Zhou, Z. Shen, Y. Liu, J. Zhang, L. Liu, H. Fu, *J. Mater. Sci. Technol.* **2022**, *111*, 298−306. https://doi.org/10.1016/j.jmst.2021.10.013.

[2] I. S. Wani, T. Bhattacharjee, S. Sheikh, P. P. Bhattacharjee, S. Guo, N. Tsuji, *Mater. Sci. Eng. : A*  **2016**, *675*, 99−109. https://doi.org/10.1016/j.msea.2016.08.048.

[3] J. Ren, Y. Zhang, D. Zhao, Y. Chen, S. Guan, Y. Liu, L. Liu, S. Peng, F. Kong, J. D. Poplawsky, G. Gao, T. Voisin, K. An, Y. M. Wang, K. Y. Xie, T. Zhu, W. Chen, *Nature* **2022**, *608*, 62−68. https://doi.org/10.1038/s41586-022-04914-8.

[4] L. Huang, Y. Sun, N. Chen, H. Luan, G. Le, X. Liu, Y. Ji, Y. Lu, P. K. Liaw, X. Yang, Y. Zhou, J. Li, *Mater. Sci. Eng. : A*  **2022**, *830*, 142327. https://doi.org/10.1016/j.msea.2021.142327.

[5] L. He, S. Wu, A. Dong, H. Tang, D. Du, G. Zhu, B. Sun, W. Yan, *J. Mater. Sci. Technol.* **2022**, *117*, 133−145. https://doi.org/10.1016/j.jmst.2021.11.049.

[6] Y. Guo, H. Su, P. Yang, Z. Shen, D. Zhao, Y. Zhao, Y. Liu, H. Zhou, *Addit. Manuf.* **2022**, *60*, 103257. https://doi.org/10.1016/j.addma.2022.103257.

[7] Z. Liang, Y. Zhang, Y. Liu, Z. Zhu, H. Zhang, *Mater. Lett.* **2022**, *317*, 132092. https://doi.org/10.1016/j.matlet.2022.132092.

[8] T. Yu, G. Zhou, Y. Cheng, F. Hu, T. Jiang, T. Sun, Y. Shen, Y. Zhou, J. Li, *Opt. Laser Technol.* **2023**, *163*, 109396. https://doi.org/10.1016/j.optlastec.2023.109396.

[9] T. Xiong, S. J. Zheng, J. Y. Pang, X. L. Ma, *Scr. Mater.* **2020**, *186*, 336−340. https://doi.org/10.1016/j.scriptamat.2020.04.035.

[10] X. Chen, J. Kong, J. Li, S. Feng, H. Li, Q. Wang, Y. Liang, K. Dong, Y. Yang, *Mater. Sci. Eng. : A*  **2022**, *854*, 143816. https://doi.org/10.1016/j.msea.2022.143816.

[11] Y. Zhu, S. Zhou, Z. Xiong, Y.-J. Liang, Y. Xue, L. Wang, *Addit. Manuf.* **2021**, *39*, 101901. https://doi.org/10.1016/j.addma.2021.101901.

[12] L. Lan, W. Wang, Z. Cui, X. Hao, *Mater. Lett.* **2023**, *330*, 133321. https://doi.org/10.1016/j.matlet.2022.133321.

[13] Y. Lu, X. Wu, Z. Fu, Q. Yang, Y. Zhang, Q. Liu, T. Li, Y. Tian, H. Tan, Z. Li, T. Wang, T. Li, *J. Mater. Sci. Technol.* **2022**, *126*, 15−21. https://doi.org/10.1016/j.jmst.2022.04.004.

[14] S. L. Sing, W. Y. Yeong, F. E. Wiria, *J. Alloy. Compd.* **2016**, *660*, 461−470. https://doi.org/10.1016/j.jallcom.2015.11.141.

[15] H. Suo, Z. Chen, J. Liu, S. Gong, J. Xiao, *Rare Metal Mater. Eng.* **2014**, *43*, 780−785. https://doi.org/10.1016/s1875-5372(14)60083-7.

[16] L. C. Zhang, D. Klemm, J. Eckert, Y. L. Hao, T. B. Sercombe, *Scr. Mater.* **2011**, *65*, 21−24. https://doi.org/10.1016/j.scriptamat.2011.03.024.

[17] B. Wysocki, P. Maj, R. Sitek, J. Buhagiar, K. Kurzydłowski, W. Święszkowski, *Appl. Sci.* **2017**, *7*, 657. https://doi.org/10.3390/app7070657.

[18] J. C. Wang, Y. J. Liu, S. X. Liang, Y. S. Zhang, L. Q. Wang, T. B. Sercombe, L. C. Zhang, *J. Mater. Sci. Technol.* **2022**, *105*, 1−16. https://doi.org/10.1016/j.jmst.2021.07.021.

[19] H. Y. Ma, J. C. Wang, P. Qin, Y. J. Liu, L. Y. Chen, L. Q. Wang, L. C. Zhang, *J. Mater. Sci. Technol.* **2024**, *183*, 32−62. https://doi.org/10.1016/j.jmst.2023.11.003.

[20] H. D. Nguyen, A. Pramanik, A. K. Basak, Y. Dong, C. Prakash, S. Debnath, S. Shankar, I. S. Jawahir, S. Dixit, D. Buddhi, *J. Mater. Res. Technol.* **2022**, *18*, 4641−4661. https://doi.org/10.1016/j.jmrt.2022.04.055.

[21] W. W. Sun, Y. X. Wu, S. C. Yang, C. R. Hutchinson, *Scr. Mater.* **2018**, *146*, 60−63. https://doi.org/10.1016/j.scriptamat.2017.11.007.

[22] R. Kuziak, R. Kawalla, S. Waengler, *Arch. Civ. Mech. Eng.* **2008**, *8*, 103−117. https://doi.org/10.1016/s1644-9665(12)60197-6.

[23] A. Kalhor, A. Karimi Taheri, H. Mirzadeh, V. Uthaisangsuk, *Mater. Sci. Techno.* **2021**, *37*, 561−591. https://doi.org/10.1080/02670836.2021.1944524.

[24] A. R. Marder, *Metall. Trans. A* **1982**, *13*, 85−92.

[25] C. C. Tasan, M. Diehl, D. Yan, M. Bechtold, F. Roters, L. Schemmann, C. Zheng, N. Peranio, D. Ponge, M. Koyama, K. Tsuzaki, D. Raabe, *Annu. Rev. Mater. Res.* **2015**, *45*, 391−431. https://doi.org/10.1146/annurev-matsci-070214-021103.

[26] T. Dursun, C. Soutis, *Mater. Des. (1980-2015)* **2014**, *56*, 862−871. https://doi.org/10.1016/j.matdes.2013.12.002.

[27] X. Zhang, Y. Chen, J. Hu, *Prog. Aerosp. Sci.* **2018**, *97*, 22−34. https://doi.org/10.1016/j.paerosci.2018.01.001.

[28] G. Appa Rao, M. Srinivas, D. S. Sarma, *Mater. Sci. Eng. : A*  **2004**, *383*, 201−212. https://doi.org/10.1016/j.msea.2004.05.062.

[29] Ö. Özgün, H. Özkan Gülsoy, R. Yilmaz, F. Findik, *J. Alloy. Compd.* **2013**, *546*, 192−207. https://doi.org/10.1016/j.jallcom.2012.08.069.

[30] C. Wagner, G. Laplanche, *Int. J. Plast.* **2023**, *166*, 103651. https://doi.org/10.1016/j.ijplas.2023.103651.

[31] G. Laplanche, A. Kostka, C. Reinhart, J. Hunfeld, G. Eggeler, E. P. George, *Acta Mater.* **2017**, *128*, 292−303. https://doi.org/10.1016/j.actamat.2017.02.036.

[32] C.-C. Juan, M.-H. Tsai, C.-W. Tsai, C.-M. Lin, W.-R. Wang, C.-C. Yang, S.-K. Chen, S.-J. Lin, J.-W. Yeh, *Intermetallics* **2015**, *62*, 76−83. https://doi.org/10.1016/j.intermet.2015.03.013.

[33] B. Schuh, B. Völker, J. Todt, N. Schell, L. Perrière, J. Li, J. P. Couzinié, A. Hohenwarter, *Acta Mater.* **2018**, *142*, 201−212. https://doi.org/10.1016/j.actamat.2017.09.035.

[34] B. Gwalani, V. Soni, M. Lee, S. A. Mantri, Y. Ren, R. Banerjee, *Mater. Des.* **2017**, *121*, 254−260. https://doi.org/10.1016/j.matdes.2017.02.072.

[35] J. Y. He, W. H. Liu, H. Wang, Y. Wu, X. J. Liu, T. G. Nieh, Z. P. Lu, *Acta Mater.* **2014**, *62*, 105−113. https://doi.org/10.1016/j.actamat.2013.09.037.

[36] Z. Li, K. G. Pradeep, Y. Deng, D. Raabe, C. C. Tasan, *Nature* **2016**, *534*, 227−30. https://doi.org/10.1038/nature17981.

[37] K. W. Kang, A. X. Li, J. S. Zhang, M. K. Xu, D. Huang, S. K. Liu, Y. T. Jiang, G. Li, *Mater. Sci. Eng. : A*  **2025**, *943*, 148755. https://doi.org/10.1016/j.msea.2025.148755.

[38] K. Ming, X. Bi, J. Wang, *Scr. Mater.* **2017**, *137*, 88−93. https://doi.org/10.1016/j.scriptamat.2017.05.019.

[39] P. Li, A. Wang, C. T. Liu, *J. Alloy. Compd.* **2017**, *694*, 55−60. https://doi.org/10.1016/j.jallcom.2016.09.186.

[40] R. Fan, E. Guo, L. Wang, L. Wang, S. Zhao, X. Li, X. Zhang, B. Cui, *Mater. Sci. Eng. : A*  **2023**, *882*, 145446. https://doi.org/10.1016/j.msea.2023.145446.

[41] V. Gerold, H. P. Karnthaler, *Acta Metall.* **1989**, *37(8),* 2177−2183. https://doi.org/10.1016/0001-6160(89)90143-0

[42] A. Roy, R. Koripelli, J. Pal, *Int. J. Hydrogen Energy* **2008**, 945−952. https://doi.org/10.1016/j.ijhydene.2007.11.022.

[43] R. R. Jensen, J. K. Tien, *Metall. Trans. A* **1985**, *16*, 1049−1068. https://doi.org/10.1007/BF02811675

[44] W. Kai, X. TingDong, W. YanQing, D. JinHui, *Philos. Mag. Lett.* **2009**, *89*, 725−733. https://doi.org/10.1080/09500830903277180.

[45] E. Shapiro, G. E. Dieter, *Metall. Trans.* **1970**, *1*, 1711−1719. https://doi.org/10.1007/BF02642021.

[46] K. Wang, T. Xu, S. Song, C. Shao, *Mater. Charact.* **2011**, *62*, 575−581. https://doi.org/10.1016/j.matchar.2011.03.008.

[47] G. A. Rao, M. Srinivas, D. S. Sarma, *Mater. Sci. Eng. : A*  **2006**, *435-436*, 84−99. https://doi.org/10.1016/j.msea.2006.07.053.

[48] G. S. L. Ben Mostefa, M.P. Solignac, J.P. Colin, *Acta Metall. Mater.* **1991**, *39*, 3111−3118. <https://doi.org/10.1016/0956-7151(91)90044-2>.

[49] L. Zheng, G. Schmitz, Y. Meng, R. Chellali, R. Schlesiger, *Crit. Rev. Solid State Mater. Sci.* **2012**, *37*, 181−214. https://doi.org/10.1080/10408436.2011.613492.

[50] T. Xu, L. Zheng, K. Wang, R. D. K. Misra, *Int.l Mater. Rev.* **2013**, *58*, 263−295. https://doi.org/10.1179/1743280412y.0000000014.

[51] V. V. Zabil'skii, *Met. Sci. Heat Treat.* **1987**, *29*, 32−42. https://doi.org/10.1007/BF00735489.

[52] Y. Fan, T. G. Liu, L. Xin, Y. M. Han, Y. H. Lu, T. Shoji, *J. Nuclear Mater.* **2021**, *544*, 152693. https://doi.org/10.1016/j.jnucmat.2020.152693.

[53] P. A. F Danoix, *Mater. Charact.* **2000**, *44*, 177−201. <https://doi.org/10.1016/S1044-5803(99)00048-0>.

[54] B. C. M. S.P Lynch, T Pasang, *Acta Mater.* **2001**, *49*, 2863−2874. <https://doi.org/10.1016/S1359-6454(01)00217-8>.

[55] Y. Lu, Y. Dong, S. Guo, L. Jiang, H. Kang, T. Wang, B. Wen, Z. Wang, J. Jie, Z. Cao, H. Ruan, T. Li, *Sci. Rep.* **2014**, *4*, 6200. https://doi.org/10.1038/srep06200.

[56] T. Bhattacharjee, I. S. Wani, S. Sheikh, I. T. Clark, T. Okawa, S. Guo, P. P. Bhattacharjee, N. Tsuji, *Sci. Rep.* **2018**, *8*, 3276. https://doi.org/10.1038/s41598-018-21385-y.

[57] X. Gao, Y. Lu, B. Zhang, N. Liang, G. Wu, G. Sha, J. Liu, Y. Zhao, *Acta Mater.* **2017**, *141*, 59−66. https://doi.org/10.1016/j.actamat.2017.07.041.

[58] Y. Lu, X. Gao, L. Jiang, Z. Chen, T. Wang, J. Jie, H. Kang, Y. Zhang, S. Guo, H. Ruan, Y. Zhao, Z. Cao, T. Li, *Acta Mater.* **2017**, *124*, 143−150. https://doi.org/10.1016/j.actamat.2016.11.016.

[59] I. S. Wani, T. Bhattacharjee, S. Sheikh, I. T. Clark, M. H. Park, T. Okawa, S. Guo, P. P. Bhattacharjee, N. Tsuji, *Intermetallics* **2017**, *84*, 42−51. https://doi.org/10.1016/j.intermet.2016.12.018.

[60] I. S. Wani, T. Bhattacharjee, S. Sheikh, Y. P. Lu, S. Chatterjee, P. P. Bhattacharjee, S. Guo, N. Tsuji, *Mater. Res. Lett.* **2016**, *4*, 174−179. https://doi.org/10.1080/21663831.2016.1160451.

[61] X. Jin, Y. Zhou, L. Zhang, X. Du, B. Li, *Mater. Des.* **2018**, *143*, 49−55. https://doi.org/10.1016/j.matdes.2018.01.057.

[62] Q. Liu, X. Liu, X. Fan, R. Li, X. Tong, P. Yu, G. Li, *J. Alloy. Compd.* **2022**, *904*, 163775. https://doi.org/10.1016/j.jallcom.2022.163775.

[63] I. Baker, F. Meng, M. Wu, A. Brandenberg, *J. Alloy. Compd.* **2016**, *656*, 458−464. https://doi.org/10.1016/j.jallcom.2015.09.264.

[64] J. W. Lili Ma, Peipeng Jin, *Mater. Res. Express* **2020**, *7*, 016566. <https://doi.org/10.1088/2053-1591/ab6580>.

[65] X. Jin, J. Bi, L. Zhang, Y. Zhou, X. Du, Y. Liang, B. Li, *J. Alloy. Compd.* **2019**, *770*, 655−661. https://doi.org/10.1016/j.jallcom.2018.08.176.

[66] Z. Geng, C. Chen, M. Song, J. Luo, J. Chen, R. Li, K. Zhou, *J. Mater. Sci. Technol.* **2024**, *187*, 141−155. https://doi.org/10.1016/j.jmst.2023.11.054.

[67] U. Sunkari, S. R. Reddy, K. S. Athira, S. Chatterjee, P. P. Bhattacharjee, *Mater. Sci. Eng. : A*  **2020**, *793*, 139897. https://doi.org/10.1016/j.msea.2020.139897.

[68] Y. Dong, Y. Lu, J. Kong, J. Zhang, T. Li, *J. Alloy. Compd.* **2013**, *573*, 96−101. https://doi.org/10.1016/j.jallcom.2013.03.253.

[69] Y. Dong, L. Jiang, H. Jiang, Y. Lu, T. Wang, T. Li, *Mater. Des.* **2015**, *82*, 91−97. https://doi.org/10.1016/j.matdes.2015.05.046.

[70] L. Jiang, Y. Lu, Y. Dong, T. Wang, Z. Cao, T. Li, *Appl. Phys. A* **2015**, *119*, 291−297. https://doi.org/10.1007/s00339-014-8964-4.

[71] L. Jiang, Y. Lu, W. Wu, Z. Cao, T. Li, *J. Mater. Sci. Technol.* **2016**, *32*, 245−250. https://doi.org/10.1016/j.jmst.2015.08.006.

[72] Y. Yin, Q. Tan, T. Wang, D. Kent, N. Mo, M. Bermingham, H. Li, M.-X. Zhang, *J. Mater. Sci.* **2020**, *55*, 14571−14587. https://doi.org/10.1007/s10853-020-05025-3.

[73] Y. Yin, D. Kent, Q. Tan, M. Bermingham, M.-X. Zhang, *J. Mater. Sci. Technol.* **2020**, *51*, 173−179. https://doi.org/10.1016/j.jmst.2020.01.066.

[74] H. Jiang, K. Han, D. Qiao, Y. Lu, Z. Cao, T. Li, *Mater. Chem. Phys.* **2018**, *210*, 43−48. https://doi.org/10.1016/j.matchemphys.2017.05.056.

[75] H. Jiang, H. Zhang, T. Huang, Y. Lu, T. Wang, T. Li, *Mater. Des.* **2016**, *109*, 539−546. https://doi.org/10.1016/j.matdes.2016.07.113.

[76] R. Li, J. Ren, G.-J. Zhang, J.-Y. He, Y.-P. Lu, T.-M. Wang, T.-J. Li, *Acta Metall. Sin. (Engl. Lett.)* **2020**, *33*, 1046−1056. https://doi.org/10.1007/s40195-020-01072-6.

[77] Y. Guo, L. Liu, Y. Zhang, J. Qi, B. Wang, Z. Zhao, J. Shang, J. Xiang, *J. Mater. Res.* **2018**, *33*, 3258-3265. https://doi.org/10.1557/jmr.2018.177.

[78] S. G. Ma, Y. Zhang, *Mater. Sci. Eng. : A*  **2012**, *532*, 480−486. https://doi.org/10.1016/j.msea.2011.10.110.

[79] J. M. Zhu, H. F. Zhang, H. M. Fu, A. M. Wang, H. Li, Z. Q. Hu, *J. Alloy. Compd.* **2010**, *497*, 52−56. https://doi.org/10.1016/j.jallcom.2010.03.074.

[80] S. Liu, M. C. Gao, P. K. Liaw, Y. Zhang, *J. Alloy. Compd.* **2015**, *619*, 610−615. https://doi.org/10.1016/j.jallcom.2014.09.073.

[81] P. Kelly, *Int. Metall. Rev* **1973**, *18*, 31–36.

[82] T. Gladman, *Mater. Sci. Technol* **1999**, *15*, 30–36.

[83] G. G. C. G. Rhodes, *Metall. Trans.* **1972**, *3*, 1861−1868. https://doi.org/10.1007/BF02642571.

[84] P. Wang, L. Deng, K. G. Prashanth, S. Pauly, J. Eckert, S. Scudino, *J. Alloy. Compd.* **2018**, *735*, 2263-2266. https://doi.org/10.1016/j.jallcom.2017.10.168.

[85] Hans Warlimont, Werner Martienssen, Springer Handbook of Materials Data (2nd Edition), Springer Cham, ISBN: 978-3-319-69743-7. https://doi.org/10.1007/978-3-319-69743-7

[86] J. Wang, J. Zhu, Y. Liu, H. Peng, X. Su, *J. Mater. Res.* **2018**, *33*, 1773−1781. https://doi.org/10.1557/jmr.2018.144.

[87] Z. Jiaqing, L. Ya, P. Haoping, W. Jianhua, S. Xuping, *Mater. Res. Express* **2017**, *4*, 106505. https://doi.org/10.1088/2053-1591/aa8d4d.

[88] T. S. L. C. M. Chuang, L. H. Chen, **2001**, *16*, 2644-2652. https://doi.org/10.1557/JMR.2001.0363.

[89] M. M. Hasan, A. Sharif, M. A. Gafur, *J. Mater. Sci. : Mater. Electron.* **2019**, *31*, 1691−1702. https://doi.org/10.1007/s10854-019-02687-x.

[90] A. P. Hekimoğlu, T. Savaşkan, *Int. J. Mater. Res.* **2014**, *105*, 1084−1088. <https://doi.org/10.3139/146.111116>.

[91] J. Z. He Wu, Dong Huang, Yafang Han, *Foundry* **2003**, *52*, 332−335.

[92] L. W. Wang Hongxia, Zhao Qi, Yin Yaopeng, Zhao Xingguo, *Rare Metal Mater. Eng.* **2008**, *37*, 2004−2007.

[93] T. Xiong, W. Yang, S. Zheng, Z. Liu, Y. Lu, R. Zhang, Y. Zhou, X. Shao, B. Zhang, J. Wang, F. Yin, P. K. Liaw, X. Ma, *J. Mater. Sci. Technol.* **2021**, *65*, 216−227. https://doi.org/10.1016/j.jmst.2020.04.073.

[94] J. l. Cahn, D. l. Hoffman, *Acta Metall.* **1974**, *22*, 1205−1214. <https://doi.org/10.1016/0001-6160(74)90134-5>.

[95] J. D. Robson, *Acta Mater.* **2013**, *61*, 7781−7790. https://doi.org/10.1016/j.actamat.2013.09.017.

[96] S. J. Zheng, J. Wang, J. S. Carpenter, W. M. Mook, P. O. Dickerson, N. A. Mara, I. J. Beyerlein, *Acta Mater.* **2014**, *79*, 282−291. https://doi.org/10.1016/j.actamat.2014.07.017.

[97] N. N. M. Enomoto, *Scr. Mater.* **1997**, *36*, 625−632. <https://doi.org/10.1016/S1359-6462(96)00432-0>.

[98] M. Perez, *Scr. Mater.* **2005**, *52*, 709−712. https://doi.org/10.1016/j.scriptamat.2004.12.026.

[99] Q. Zuo, F. Liu, L. Wang, C. F. Chen, Z. H. Zhang, *J. Mater. Sci.* **2014**, *49*, 3066−3079. https://doi.org/10.1007/s10853-013-8009-y.
